# Bioactivity of Novel Pyrazole-Thiazolines Scaffolds against *Trypanosoma cruzi*: Computational Approaches and 3D Spheroid Model on Drug Discovery for Chagas Disease

**DOI:** 10.3390/pharmaceutics14050995

**Published:** 2022-05-05

**Authors:** Leonardo da Silva Lara, Guilherme Curty Lechuga, Lorraine Martins Rocha Orlando, Byanca Silva Ferreira, Bernardo Araújo Souto, Maurício Silva dos Santos, Mirian Claudia de Souza Pereira

**Affiliations:** 1 Laboratório de Ultraestrutura Celular, Instituto Oswaldo Cruz, Fiocruz, Av. Brasil 4365, Manguinhos, Rio de Janeiro 21040-900, Brazil; leonardosilva.lara@hotmail.com (L.d.S.L.); guilherme.lechuga@yahoo.com.br (G.C.L.); lorrainemartins07@hotmail.com (L.M.R.O.); 2 Laboratório de Síntese de Sistemas Heterocíclicos (LaSSH), Instituto de Física e Química, Universidade Federal de Itajubá, Avenida BPS, 1303, Pinheirinho, Itajubá 37500-903, Brazil; byancaferreira@outlook.com (B.S.F.); araujoso@ualberta.ca (B.A.S.); mauriciosantos@unifei.edu.br (M.S.d.S.)

**Keywords:** *Trypanosoma cruzi*, pyrazole, thiazoline, chemotherapy, Chagas disease

## Abstract

Chagas disease, a century-old disease that mainly affects the impoverished population in Latin America, causes high morbidity and mortality in endemic countries. The available drugs, benznidazole (Bz) and nifurtimox, have limited effectiveness and intense side effects. Drug repurposing, and the development of new chemical entities with potent activity against *Trypanosoma cruzi*, are a potential source of therapeutic options. The present study describes the biological activity of two new series of pyrazole-thiazoline derivatives, based on optimization of a hit system 5-aminopyrazole-imidazoline previously identified, using structure–activity relationship exploration, and computational and phenotype-based strategies. Promising candidates, **2c**, **2e**, and **2i** derivatives, showed good oral bioavailability and ADMET properties, and low cytotoxicity (CC_50_ > 100 µM) besides potent activity against trypomastigotes (0.4–2.1 µM) compared to Bz (19.6 ± 2.3 µM). Among them, **2c** also stands out, with greater potency against intracellular amastigotes (pIC_50_ = 5.85). The selected pyrazole-thiazoline derivatives showed good permeability and effectiveness in the 3D spheroids system, but did not sustain parasite clearance in a washout assay. The compounds’ mechanism of action is still unknown, since the treatment neither increased reactive oxygen species, nor reduced cysteine protease activity. This new scaffold will be targeted to optimize in order to enhance its biological activity to identify new drug candidates for Chagas disease therapy.

## 1. Introduction

Chagas disease (CD) is a potentially fatal infection, caused by the flagellated protozoan *Trypanosoma cruzi*, that affects about 6–7 million people worldwide, leading to more than 10,000 annual deaths [1]. This pathology is considered a serious public health problem, representing one of the biggest causes of heart failure and sudden death in many endemic countries in Latin America [2].

With the increase in migratory mobility, the disease has been increasingly reported in non-endemic countries, such as Canada, Australia, Spain, Japan, and the United States of America, among others [3]. As a result of areas with active transmission, and the high number of people without diagnosis and treatment, it is estimated that 75 million people are at risk of infection [1].

Although vector-borne transmission, which occurs during the blood meal of triatomine bugs, has been mostly controlled in some endemic countries [4], *T. cruzi* oral infection, by ingestion of food contaminated with infected triatomines or their feces, in addition to congenital (mother-to-child) *T. cruzi* infection, emerged as potential transmission routes [5]. 

The acute phase of the disease, characterized by positive parasitemia, lasts approximately 60 days, usually with mild and nonspecific symptoms, which can make diagnosis and early treatment difficult [6,7]. About 30–40% of infected individuals will develop a chronic symptomatic phase, characterized by serious cardiac, digestive, neurological, or mixed clinical manifestations, which cause significant morbidity and mortality [1,8].

The available etiological treatment is restricted to two antiparasitic drugs: benznidazole (Bz) and nifurtimox (Nif), which were clinically introduced more than 50 years ago. Both drugs are partially effective in acute cases (60–80%), in congenital cases, and in reactivation by immunosuppression [9]. However, the occurrence of serious adverse effects and the long-term treatment period often lead to treatment discontinuity [10,11]. To date, clinical trials pointed to the low efficacy of Bz and Nif in the chronic phase of the disease [12]. The BENznidazole Evaluation for Interrupting Trypanosomiasis (BENEFIT) trial, a randomized placebo-controlled clinical trial with patients in the chronic phase of Chagas disease, revealed the failure of Bz to inhibit the chronic Chagas cardiomyopathy progression [13]. Therapeutic failure of the most promising azole candidates, ergosterol biosynthesis inhibitors, has been reported in phase II randomized clinical trials [14,15]. Monotherapy with posaconazole [14] and fosravuconazole (E1224) [15] did not show efficacy, but only a temporary suppression of the parasite burden. Furthermore, the combination therapy of Bz with posaconazole has shown no additional benefits compared to Bz monotherapy [16]. The data from the BEnznidazole New Doses Improved Treatment and Associations (BENDITA) trial brought a new perspective of treatment with Bz, showing efficacy with low doses and short treatment schedules [17]. Fexinidazole, a drug approved for human African trypanosomiasis [18], sustained trypanocidal activity, but the treatment was interrupted due to safety and tolerability issues. Thus, a new fexinidazole clinical study was conducted with a low-dose, short-term treatment regimen, which was expected to be completed in 2021 [19]. This scenario highlights the urgent need for new, safer, and more efficient drugs for the treatment of Chagas disease.

Pyrazoles and their derivatives stand out in the field of medicinal chemistry [20], as they represent a relevant class of compounds with broad biological activities already reported as antioxidants [21], antiviral [22], antimicrobial [23], anticancer [24], and anti-inflammatory [25], among others. Pyrazole derivatives have been highlighted for their effects on trypanosomatids [26]. Pyrazole-trifluoromethylated hybrids have potent activity against *Leishmania amazonensis* and *T. cruzi*, with the pyrazole 2-amino-1,3,4-thiadiazole hybrids considered as a relevant scaffold for optimization [27]. The in vitro biological activity of dialkyl pyrazole-3,5-dicarboxylates analogues has also been demonstrated against *T. cruzi*, *Leishmania infantum* and *Leishmania braziliensis*, showing a remarkable inhibition of iron superoxide dismutase (Fe-SOD) activity that is responsible for the antioxidant defense mechanism of the parasite. Two analogues showed efficacy in a murine model of acute and chronic Chagas disease, with suppressive activity on the parasitemia (95%) even after animal immunosuppression [28]. Additionally, potent trypanocidal activity was evidenced by *N*-ethylurea pyrazole derivatives against *T. brucei* and *T. cruzi*, drastically reducing the parasitic burden in experimental acute infection by *T. cruzi* [29]. Also, we have recently reported the trypanocidal activity of pyrazole-imidazoline derivatives, showing low mammalian cell toxicity, and a prediction of good oral bioavailability by in silico analysis of physicochemical and ADMET properties [30]. The promising analogues bind by hydrogen bond and hydrophobic interactions to the active site of cruzipain, and significantly inhibit cysteine protease activity. These derivatives have been used by our research group as a hit system to synthesize new compounds through rational optimization, including ring bioisosterism, functional group interconversion, and functional group removal [30,31]. 

In this study, we demonstrate the trypanocidal activity and computational data of two new series of pyrazole-thiazoline derivatives, **1(a-l)** and **2(a-l)**. A three-dimensional (3D) in vitro model and reversibility assay were also performed to evaluate the permeability and efficacy in microtissue and parasite recrudescence.

## 2. Materials and Methods

### 2.1. Chemistry

All reagents and solvents were used without further purification. The reactions were followed by thin layer chromatography on silica gel-precoated aluminum plates (type 60-F254). Melting points are uncorrected, and were determined on a Fisatom 430 (Fisatom, São Paulo, SP, Brazil) or Allerbest PF0100 (Allerbest, Curitiba, PR, Brazil) equipment. FT-IR spectra were acquired on a PerkinElmer Spectrum 100, ATR diamond-ZnSe apparatus (PerkinElmer, Waltham, MA, USA). NMR spectra were performed on a Bruker Avance (Bruker, Rheinstetten, Germany), 400 or 500 MHz, at 298 K, CDCl_3_ as solvent, and TMS as internal reference. Chemical shifts (δ) are expressed in parts per million (ppm), and coupling constants (*J*) in Hertz (Hz). HRMS spectra were recorded on a Micromass/Waters ZQ-4000 spectrometer (Waters, Milford, MA, USA) (Supplementary Figure S1).

The key intermediates, 5-amino-1-aryl-1*H*-pyrazole-4-carbonitriles **4(a-l)** and 1-aryl-1*H*-pyrazole-4-carbonitriles **5(a-l)**, were synthesized by our research group according to the methodologies previously published [32,33].

*General procedure for synthesis of 2-(5-amino-1-aryl-1H-pyrazole-4-yl)-4,5-dihydrothiazoles* **1(a-l)** *and 2-(1-aryl-1H-pyrazole-4-yl)-4,5-dihydrothiazoles* **2(a-l)**

**Figure 1:**
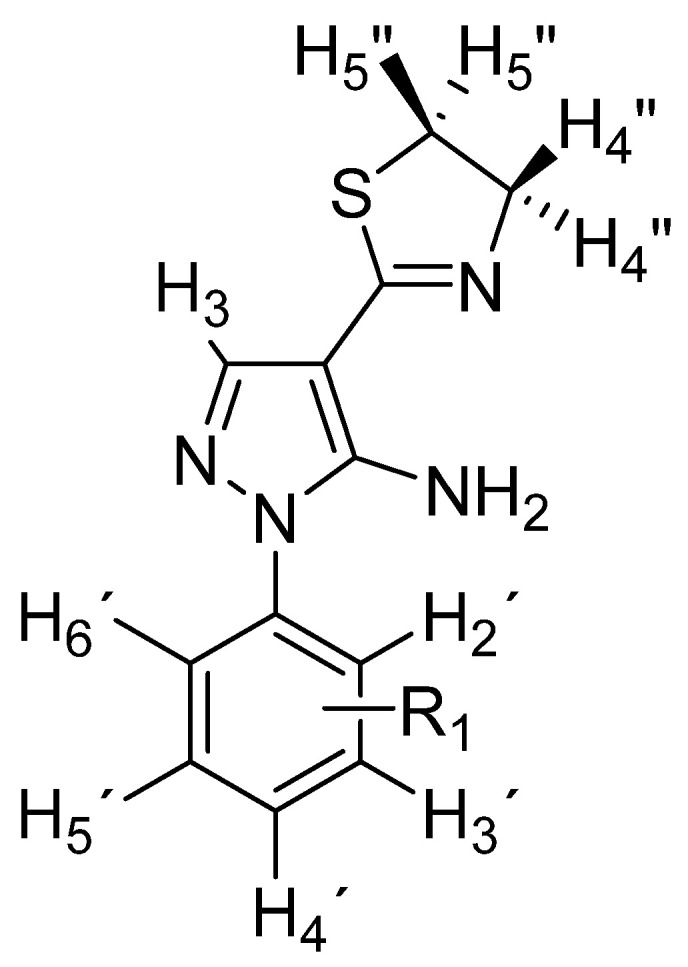
Molecular structure of series **1(a-l)** with hydrogen atoms highlighted.

**Figure 2:**
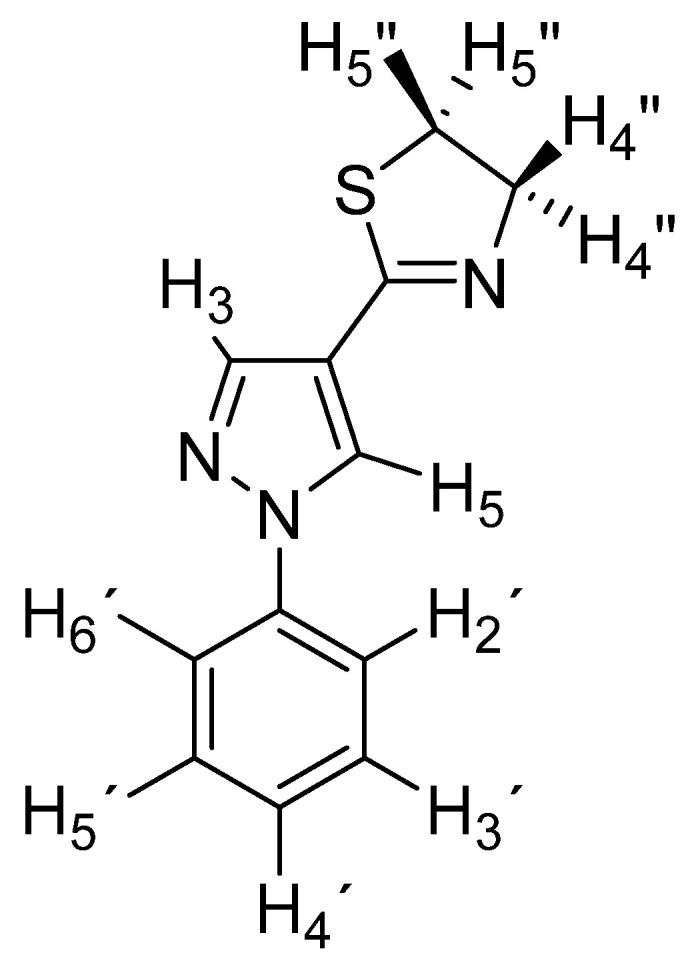
Molecular structure of series **2(a-l)** with hydrogen atoms highlighted.

A mixture of 2-aminoethanethiol hydrochloride (2 mmol), potassium carbonate (1 mmol), and methanol (5 mL) was stirred and refluxed for 30 min. Subsequently, the key intermediate, **4(a-l)** or **5(a-l)** (1 mmol), was added, and the reaction was accompanied by TLC. After 3–6 h, the mixture was poured into cold water. The product was filtered out and washed with cold water. In order to facilitate the assignment of signals in ^1^H NMR spectra, the hydrogen atoms of compounds **1(a-l)** and **2(a-l)** were highlighted (Figure 1 and Figure 2).

*2-(5-amino-1-phenyl-1H-pyrazol-4-yl)-4,5-dihydrothiazole* **(1a)**

Yield: 87%. m.p.: 176–178 °C. FT-IR ν (cm^−1^): 3342, 3249, 3184, 3052, 2937, 2855, 1600, 1546–1454. ^1^H NMR (400 MHz, CDCl_3_) δ 7.61 (s, H_3_), 7.58–7.49 and 7.41–7.37 (m, H_2_′-H_6_′), 5.67 (br, NH_2_), 4.33 (t, *J* = 8.0 Hz, H_4_″), 3.34 (t, *J* = 8.0 Hz, H_5_″). ^13^C NMR (100 MHz, CDCl_3_) δ 161.8, 145.8, 140.5, 137.9, 129.7, 127.9, 123.6, 99.2, 63.7, 33.0. HRMS (ESI) *m*/*z* [M+H]^+^ = 245.0856 (found), [M+H]^+^ = 245.0855 (calculated).

*2-(5-amino-1-(3-chlorophenyl)-1H-pyrazol-4-yl)-4,5-dihydrothiazole* **(1b)**

Yield: 90%. m.p.: 186–189 °C. FT-IR ν (cm^−1^): 3346, 3265, 3194, 3068, 2938, 2855,1596, 1544–1442. ^1^H NMR (400 MHz, CDCl_3_) δ 7.62 (t, *J* = 2.0 Hz, H_2_′), 7.60 (s, H_3_), 7.50–7.48 (m, H_6_′), 7.43 (t, *J* = 7.9 Hz, H_5_′), 7.37–7.34 (m, H_4_′), 5.72 (br, NH_2_), 4.33 (t, *J* = 8.1 Hz, H_4_″), 3.34 (t, *J* = 8.1 Hz, H_5_″). ^13^C NMR (100 MHz, CDCl_3_) δ 161.7, 145.9, 140.9, 139.1, 135.5, 130.7, 127.9, 123.7, 121.2, 99.5, 63.7, 33.1. HRMS (ESI) *m*/*z* [M+H]^+^ = 279.0460 (found), [M+H]^+^ = 279.0466 (calculated).

*2-(5-amino-1-(2,4-dichlorophenyl)-1H-pyrazol-4-yl)-4,5-dihydrothiazole* **(1c)**

Yield: 88%. m.p.: 209–212 °C. FT-IR ν (cm^−1^): 3323, 3240, 3184, 3135, 2941, 2855, 1603, 1548–1434. ^1^H NMR (400 MHz, CDCl_3_) δ 7.64 (s, H_3_), 7.59–7.58 (m, H_3_′), 7.41 (m, H_5_′, H_6_′), 5.49 (br, NH_2_), 4.32 (t, *J* = 8.0 Hz, H_4_″), 3.34 (t, *J* = 8.0 Hz, H_5_″). ^13^C NMR (100 MHz, CDCl_3_) δ 161.7, 147.1, 141.2, 136.3, 133.6, 133.0, 130.7, 130.6, 128.5, 98.5, 63.7, 33.1. HRMS (ESI) *m*/*z* [M+H]^+^ = 313.0072 (found), [M+H]^+^ = 313.0076 (calculated).

*2-(5-amino-1-(3,5-dichlorophenyl)-1H-pyrazol-4-yl)-4,5-dihydrothiazole* **(1d)**

Yield: 81%. m.p.: 232–234 °C. FT-IR ν (cm^−1^): 3410, 3293, 3087, 3070, 2929, 2850, 1584, 1522–1429. ^1^H NMR (400 MHz, CDCl_3_) δ 7.60 (s, H_3_), 7.55 (d, *J* = 1.8 Hz, H_2_′, H_6_′), 7.36 (t, *J* = 1.8 Hz, H_4_′), 5.74 (br, NH_2_), 4.33 (t, *J* = 8.1 Hz, H_4_″), 3.34 (t, *J* = 8.1 Hz, H_5_″). ^13^C NMR (100 MHz, CDCl_3_) δ 161.3, 146.0, 141.2, 139.9, 136.0, 127.6, 121.4, 99.9, 63.9, 33.1. HRMS (ESI) *m*/*z* [M+H]^+^ = 313.0067 (found), [M+H]^+^ = 313.0076 (calculated).

*2-(5-amino-1-(3,4-dichlorophenyl)-1H-pyrazol-4-yl)-4,5-dihydrothiazole* **(1e)**

Yield: 90%. m.p.: 279–282 °C. FT-IR ν (cm^−1^): 3315, 3248, 3088, 3069, 2946, 2860, 1593, 1543–1430. ^1^H NMR (400 MHz, DMSO-*d_6_*) δ 7.83 (d, *J* = 2.5 Hz, H_2_′), 7.77 (d, *J* = 8.7 Hz, H_5_′) 7.59 (s, H_3_), 7.61–7.59 (m, H_6_′), 6.69 (br, NH_2_), 4.26 (t, *J* = 8.0 Hz, H_4_″), 3.33 (t, *J* = 8.0 Hz, H_5_″). ^13^C NMR (100 MHz, DMSO-*d_6_*) δ 159.8, 146.8, 140.6, 137.8, 131.6, 131.1, 129.4, 124.6, 123.1, 98.3, 63.6, 32.2. HRMS (ESI) *m*/*z* [M+H]^+^ = 313.0074 (found), [M+H]^+^ = 313.0076 (calculated).

*2-(5-amino-1-(4-chlorophenyl)-1H-pyrazol-4-yl)-4,5-dihydrothiazole* **(1f)**

Yield: 82%. m.p.: 283–285 °C. FT-IR ν (cm^−1^): 3328, 3240, 3184, 3144, 3065, 2932, 2856, 1603, 1545–1432. ^1^H NMR (400 MHz, CDCl_3_) δ 7.60 (s, H_3_), 7.53 (d, *J* = 8.8 Hz, H_2_′, H_6_′), 7.48 (d, *J* = 8.8 Hz, H_3_′, H_5_′), 5.67 (br, NH_2_), 4.33 (t, *J* = 8.0 Hz, H_4_″), 3.34 (t, *J* = 8.0 Hz, H_5_″). ^13^C NMR (100 MHz, CDCl_3_) δ 161.8, 145.9, 140.7, 136.5, 133.6, 129.9, 124.8, 99.4, 63.6, 33.0. HRMS (ESI) *m*/*z* [M+H]^+^ = 279.0464 (found), [M+H]^+^ = 279.0466 (calculated).

*2-(5-amino-1-(4-fluorophenyl)-1H-pyrazol-4-yl)-4,5-dihydrothiazole* **(1g)**

Yield: 86%. m.p.: 244–246 °C. FT-IR ν (cm^−1^): 3342, 3254, 3194, 3141, 2947, 2858, 1599, 1547–1434. ^1^H NMR (400 MHz, CDCl_3_) δ 7.59 (s, H_3_), 7.56–7.52 (m, H_2_′, H_6_′), 7.20 (t, *J* = 8.6 Hz, H_3_′, H_5_′), 5.57 (br, NH_2_), 4.32 (t, *J* = 8.0 Hz, H_4_″), 3.33 (t, *J* = 8.0 Hz, H_5_″). ^13^C (100 MHz, CDCl_3_) δ 161.8 (d, *J* = 248.4 Hz), 161.5, 145.9, 140.4, 134.0 (d, *J* = 3.0 Hz), 125.7 (d, *J* = 8.7 Hz), 116.7 (d, *J* = 23.0 Hz), 99.4, 63.9, 33.1. HRMS (ESI) *m*/*z* [M+H]^+^ = 263.0758 (found), [M+H]^+^ = 263.0761 (calculated).

*2-(5-amino-1-(3-fluorophenyl)-1H-pyrazol-4-yl)-4,5-dihydrothiazole* **(1h)**

Yield: 94%. m.p.: 230–232 °C. FT-IR ν (cm^−1^): 3333, 3256, 3078, 2944, 2858, 1603, 1544–1438. ^1^H NMR (400 MHz, CDCl_3_) δ 7.60 (s, H_3_), 7.50–7.44 (m, H_6_′), 7.40–7.38 (m, H_5_′), 7.37–7.33 (m, H_4_′), 7.11–7.08 (m, H_2_′), 5.73 (br, NH_2_), 4.33 (t, *J* = 8.1 Hz, H_4_″), 3.34 (t, *J* = 8.1 Hz, H_5_″). ^13^C (100 MHz, CDCl_3_) δ 163.1 (d, *J* = 248.4 Hz), 161.6, 145.9, 140.8, 139.4 (d, *J* = 10.0 Hz), 131.0 (d, *J* = 9.1 Hz), 118.6 (d, *J* = 3.3 Hz), 114.7 (d, *J* = 21.1 Hz), 111.0 (d, *J* = 24.8 Hz), 99.5, 63.8, 33.1. HRMS (ESI) *m*/*z* [M+H]^+^ = 263.0765 (found), [M+H]^+^ = 263.0761 (calculated).

*2-(5-amino-1-(4-bromophenyl)-1H-pyrazol-4-yl)-4,5-dihydrothiazole* **(1i)**

Yield: 80%. m.p.: 282–284 °C. FT-IR ν (cm^−1^): 3339, 3260, 3210, 3066, 2939, 2853, 1604, 1579–1425. ^1^H NMR (400 MHz, CDCl_3_) δ 7.63 (d, *J* = 8.8 Hz, H_3_′, H_5_′), 7.60 (s, H_3_), 7.47 (d, *J* = 8.8 Hz, H_2_′, H_6_′), 5.68 (br, NH_2_), 4.33 (t, *J* = 8.0 Hz, H_4_″), 3.34 (t, *J* = 8.0 Hz, H_5_″).^13^C NMR (100 MHz, CDCl_3_) δ 161.7, 145.8, 140.8, 137.0, 132.9, 125.0, 121.5, 99.5, 63.6, 33.0. HRMS (ESI) *m*/*z* [M+H]^+^ = 322.9951 (found), [M+H]^+^ = 322.9961 (calculated).

*2-(5-amino-1-(3-bromophenyl)-1H-pyrazol-4-yl)-4,5-dihydrothiazole* **(1j)**

Yield: 93%. m.p.: 171–173 °C. FT-IR ν (cm^−1^): 3327, 3250, 3149, 3068, 2934, 2849, 1599, 1542–1440. ^1^H NMR (400 MHz, CDCl_3_) δ 7.78 (t, *J* = 1.9 Hz, H_2_′), 7.60 (s, H_3_), 7.55–7.50 (m, H_4_′, H_6_′), 7.37 (t, *J* = 8.0 Hz, H_5_′), 5.72 (br, NH_2_), 4.33 (t, *J* = 8.1 Hz, H_4_″), 3.34 (t, *J* = 8.1 Hz, H_5_″). ^13^C NMR (100 MHz, CDCl_3_) δ 161.7, 145.9, 140.9, 139.2, 130.9, 130.8, 126.6, 123.3, 121.7, 99.5, 63.7, 33.0. HRMS (ESI) *m*/*z* [M+H]^+^ = 322.9961 (found), [M+H]^+^ = 322.9961 (calculated).

*2-(5-amino-1-(4-methoxyphenyl)-1H-pyrazol-4-yl)-4,5-dihydrothiazole* **(1k)**

Yield: 76%. m.p.: 164–166 °C. FT-IR ν (cm^−1^): 3381, 3277, 3200, 3129, 3078, 2994, 2941, 2833, 1605, 1541–1434. ^1^H NMR (500 MHz, CDCl_3_) δ 7.58 (s, H_3_), 7.45 (d, *J* = 8.7 Hz, H_2_′, H_6_′), 7.01 (d, *J* = 8.7 Hz, H_3_′, H_5_′), 5.54 (br, NH_2_), 4.32 (t, *J* = 8.0 Hz, H_4_″), 3.85 (s, CH_3_), 3.33 (t, *J* = 8.0 Hz, H_5_″). ^13^C NMR (125 MHz, CDCl_3_) δ 162.0, 159.5, 146.1, 140.3, 130.9, 125.8, 115.1, 99.1, 63.8, 55.8, 33.2. HRMS (ESI) *m*/*z* [M+H]^+^ = 275.0963 (found), [M+H]^+^ = 275.0961 (calculated).

*2-(5-amino-1-(2,3-dichlorophenyl)-1H-pyrazol-4-yl)-4,5-dihydrothiazole* **(1l)**

Yield: 86%. m.p.: 188–190 °C. FT-IR ν (cm^−1^): 3330, 3242, 3136, 2940, 2844, 1604, 1582–1433. ^1^H NMR (400 MHz, CDCl_3_) δ 7.65 (s, H_3_), 7.62 (dd, *J* = 7.7, 2.0 Hz, H_4′_), 7.42–7.34 (m, H_5_′, H_6_′), 5.51 (br, NH_2_), 4.33 (t, *J* = 8.0 Hz, H_4_″), 3.35 (t, *J* = 8.0 Hz, H_5_″). ^13^C NMR (100 MHz, CDCl_3_) δ 161.8, 147.0, 141.1, 136.6, 134.6, 131.7, 131.4, 128.2, 128.1, 98.4, 63.6, 33.1. HRMS (ESI) *m*/*z* [M+H]^+^ = 313.0072 (found), [M+H]^+^ = 313.0076 (calculated).

*2-(1-phenyl-1H-pyrazol-4-yl)-4,5-dihydrothiazole* **(2a)**

Yield: 78%. m.p.: 112–114 °C. FT-IR ν (cm^−1^) 3135, 3060, 2955, 2851, 1618, 1595–1500. ^1^H NMR (400 MHz, CDCl_3_) δ 8.37 (s, H_5_), 8.05 (s, H_3_), 7.72 (dd, *J* = 8.0; 1.0 Hz, H_2_′, H_6_′), 7.48 (t, *J* = 8.0 Hz, H_3_′, H_5_′), 7.34 (t, *J* = 8.0 Hz, H_4_′), 4.40 (t, *J* = 8.2 Hz, H_4_″), 3.44 (t, *J* = 8.2 Hz, H_5_″). ^13^C NMR (100 MHz, CDCl_3_) δ 160.6, 140.6, 139.5, 129.6, 127.5, 127.3, 119.4, 118.7, 64.0, 33.7. HRMS (ESI) *m*/*z* [M+H]^+^ 230.0747 (found), [M+H]^+^ 230.0746 (calculated).

*2-(1-(3-chlorophenyl)-1H-pyrazol-4-yl)-4,5-dihydrothiazole* **(2b)**

Yield: 91%. m.p.: 88–90 °C. FT-IR ν (cm^−1^): 3136, 3062, 2958, 2847, 1616, 1589–1463. ^1^H NMR (400 MHz, CDCl_3_) δ 8.36 (s, H_5_), 8.05 (s, H_3_), 7.78 (t, *J* = 2.0 Hz, H_2_′), 7.60 (ddd, *J* = 8.1; 2.0; 1.0 Hz, H_6_′), 7.41 (t, *J* = 8.1 Hz, H_5_′), 7.31 (ddd, *J* = 8.1; 2.0; 1.0 Hz, H_4_′), 4.40 (t, *J* = 8.3 Hz, H_4_″), 3.45 (t, *J* = 8.3 Hz, H_5_″). ^13^C NMR (100 MHz, CDCl_3_) δ 160.4, 140.9, 140.3, 135.5, 130.6, 127.5, 127.3, 119.7, 119.2, 117.2, 64.0, 33.8. HRMS (ESI) *m*/*z* [M+H]^+^ = 264.0348 (found), [M+H]^+^ = 264.0357 (calculated).

*2-(1-(2,4-dichlorophenyl)-1H-pyrazol-4-yl)-4,5-dihydrothiazole* **(2c)**

Yield: 73%. m.p.: 96–98 °C. FT-IR ν (cm^−1^): 3135, 3044, 2952, 2849, 1624, 1545–1428. ^1^H NMR (400 MHz, CDCl_3_) δ 8.24 (s, H_5_), 8.07 (s, H_3_), 7.56–7.54 (m, H_3_′, H_6_′), 7.38 (dd, *J* = 8.6; 2.3 Hz, H_5_′), 4.39 (t, *J* = 8.2 Hz, H_4_″), 3.43 (t, *J* = 8.2 Hz, H_5_″). ^13^C NMR (100 MHz, CDCl_3_) δ 159.9, 140.6, 136.2, 134.9, 131.9, 130.5, 129.0, 128.4, 128.1, 118.6, 64.4, 33.8. HRMS (ESI): *m*/*z* [M+H]^+^ = 297.9966 (found), [M+H]^+^ = 297.9967(calculated).

*2-(1-(3,5-dichlorophenyl)-1H-pyrazol-4-yl)-4,5-dihydrothiazole* **(2d)**

Yield: 83%. m.p.: 134–136 °C. FT-IR ν (cm^−1^): 3172, 3071, 2945, 2853, 1625, 1583–1441. ^1^H NMR (500 MHz, CDCl_3_) δ 8.34 (s, H_5_), 8.06 (s, H_3_), 7.66 (s, H_2_′, H_6_′), 7.32 (s, H_4_′), 4.40 (t, *J* = 8.3 Hz, H_4_″), 3.46 (t, *J* = 8.3 Hz, H_5_″). ^13^C NMR (125 MHz, CDCl_3_) δ 160.4, 141.5, 141.0, 136.3, 127.8, 127.3, 119.8, 117.9, 64.3, 34.0. HRMS (ESI) *m*/*z* [M+H]^+^ = 297.9962 (found), [M+H]^+^ = 297.9967 (calculated).

*2-(1-(3,4-dichlorophenyl)-1H-pyrazol-4-yl)-4,5-dihydrothiazole* **(2e)**

Yield: 73%. m.p.: 142–144 °C. FT-IR ν (cm^−1^): 3132, 3065, 2939, 2853, 1615, 1596–1433. ^1^H NMR (400 MHz, CDCl_3_) δ 8.32 (s, H_5_), 8.05 (s, H_3_), 7.89 (dd, *J* = 2.1; 0.5 Hz, H_2_′), 7.56–7.55 (m, H_5_′, H_6_′), 4.40 (t, *J* = 8.3 Hz, H_4_″), 3.45 (t, *J* = 8.3 Hz, H_5_″). ^13^C NMR (100 MHz, CDCl_3_) δ 155.3, 136.3, 133.8, 129.0, 126.5, 126.4, 122.6, 116.5, 114.7, 113.4, 59.4, 29.1. HRMS (ESI) *m*/*z* [M+H]^+^ = 297.9967 (found), [M+H]^+^ = 297.9967 (calculated).

*2-(1-(4-chlorophenyl)-1H-pyrazol-4-yl)-4,5-dihydrothiazole* **(2f)**

Yield: 85%. m.p.: 130–133 °C. FT-IR ν (cm^−1^): 3130, 3061, 2936, 2854, 1623, 1545–1500. ^1^H NMR (500 MHz, CDCl_3_) δ 8.32 (s, H_5_), 8.04 (s, H_3_), 7.66 (d, *J* = 8.8 Hz, H_2_′, H_6_′), 7.45 (d, *J* = 8.8 Hz, H_3_′, H_5_′), 4.39 (t, *J* = 8.2 Hz, H_4_″), 3.44 (t, *J* = 8.2 Hz, H_5_″). ^13^C NMR (125 MHz, CDCl_3_) δ 160.4, 141.0, 138.2, 133.1, 129.9, 127.6, 120.7, 119.4, 64.4, 34.0. HRMS (ESI) *m*/*z* [M+H]^+^ = 264.0349 (found), [M+H]^+^ = 264.0357 (calculated).

*2-(1-(4-fluorophenyl)-1H-pyrazol-4-yl)-4,5-dihydrothiazole* **(2g)**

Yield: 71%. m.p.: 131–133 °C. FT-IR ν (cm^−1^): 3130, 3059, 2995, 2850, 1618, 1543–1511. ^1^H NMR (500 MHz, CDCl_3_) δ 8.28 (s, H_5_), 8.03 (s, H_3_), 7.68 (dd, *J* = 8.4; 4.5 Hz, H_2_′, H_6_′), 7.17 (t, *J* = 8.4 Hz, H_3_′, H_5_′), 4.39 (t, *J* = 8.2 Hz, H_4_″), 3.43 (t, *J* = 8.2 Hz, H_5_″). ^13^C NMR (125 MHz, CDCl_3_) δ 161.8 (d, *J* = 247.3 Hz), 160.4, 140.8, 136.1, 127.7, 121.5 (d, *J* = 8.4 Hz), 119.2, 116.7 (d, *J* = 23.1 Hz), 64.5, 34.0. HRMS (ESI) *m*/*z* [M+H]^+^ = 248.0642 (found); [M+H]^+^ = 248.0652 (calculated).

*2-(1-(3-fluorophenyl)-1H-pyrazol-4-yl)-4,5-dihydrothiazole* **(2h)**

Yield: 91%. m.p.: 92–94 °C. FT-IR ν (cm^−1^): 3136, 3062, 2952, 2850, 1610, 1600–1479. ^1^H NMR (500 MHz, CDCl_3_) δ 8.35 (s, H_5_), 8.05 (s, H_3_), 7.51–7.48 (m, H_5_′, H_6_′), 7.45–7.41 (m, H_2_′), 7.04 (t, *J* = 7.5 Hz, H_4′_), 4.40 (t, *J* = 8.2 Hz, H_4_″), 3.44 (t, *J* = 8.2 Hz, H_5_″). ^13^C NMR (125 MHz, CDCl_3_) δ 163.4 (d, *J* = 246.0 Hz), 160.5, 141.1, 141.0 (d, *J* = 10.2 Hz), 131.1 (d, *J* = 9.0 Hz), 127.7, 119.4, 114.7 (d, *J* = 3.0 Hz), 114.3 (d, *J* = 21.3 Hz), 107.4 (d, *J* = 26.5 Hz), 64.4, 34.0. HRMS (ESI) *m*/*z* [M+H]^+^ 248.0653 (found), [M+H]^+^ = 248.0652 (calculated).

*2-(1-(4-bromophenyl)-1H-pyrazol-4-yl)-4,5-dihydrothiazole* **(2i)**

Yield: 78%. m.p.: 124–126 °C. FT-IR ν (cm^−1^): 3132, 3060, 2990, 2844, 1614, 1545–1497. ^1^H NMR (400 MHz, CDCl_3_) δ 8.25 (s, H_5_), 8.04 (s, H_3_), 7.60 (s, H_2_′, H_3_′, H_5_′, H_6_′), 4.39 (t, *J* = 8.2 Hz, H_4_″), 3.43 (t, *J* = 8.2 Hz, H_5_″). ^13^C NMR (100 MHz, CDCl_3_) δ 159.6, 140.8, 138.5, 132.6, 127.1, 120.8, 120.6, 119.5, 64.6, 33.9. HRMS (ESI) *m*/*z* [M+H]^+^ = 307.9848 (found), [M+H]^+^ = 307.9852 (calculated).

*2-(1-(3-bromophenyl)-1H-pyrazol-4-yl)-4,5-dihydrothiazole* **(2j)**

Yield: 78%. m.p.: 104–106 °C. FT-IR ν (cm^−1^): 3136, 3062, 2936, 2845, 1616, 1582–1459. ^1^H NMR (400 MHz, CDCl_3_) δ 8.26 (s, H_5_), 8.05 (s, H_3_), 7.93 (t, *J* = 2.0 Hz, H_2_′), 7.64 (ddd, *J* = 8.0; 2.0; 1.0 Hz, H_6_′), 7.46 (ddd, *J* = 8.0; 2.0; 1.0 Hz, H_4_′), 7.34 (t, *J* = 8.0 Hz, H_5_′), 4.39 (t, *J* = 8.2 Hz, H_4_″), 3.43 (t, *J* = 8.2 Hz, H_5_″). ^13^C NMR (100 MHz, CDCl_3_) δ 159.5, 140.8, 140.5, 130.9, 130.2, 127.3, 123.3, 122.6, 119.5, 117.6, 64.6, 33.9. HRMS (ESI) *m*/*z* [M+H]^+^ = 307.9842 (found), [M+H]^+^ = 307.9852 (calculated).

*2-(1-(4-methoxyphenyl)-1H-pyrazol-4-yl)-4,5-dihydrothiazole* **(2k)**

Yield: 90%. m.p.: 114–116 °C. FT-IR ν (cm^−1^): 3128, 3060, 2957, 2840, 1617, 1544–1458. ^1^H NMR (400 MHz, CDCl_3_) δ 8.19 (s, H_5_), 8.01 (s, H_3_), 7.60 (d, *J* = 9.0 Hz, H_2_′, H_6_′), 6.98 (d, *J* = 9.0 Hz, H_3_′, H_5_′), 4.38 (t, *J* = 8.2 Hz, H_4_″), 3.85 (s, CH_3_), 3.41 (t, *J* = 8.2 Hz, H_5_″). ^13^C NMR (100 MHz, CDCl_3_) δ 159.9, 158.8, 140.1, 133.2, 127.2, 121.1, 118.7, 114.6, 64.6, 55.6, 33.8. HRMS (ESI) *m*/*z* [M+H]^+^ = 260.0856 (found), [M+H]^+^ = 260.0852 (calculated).

*2-(1-(2,3-dichlorophenyl)-1H-pyrazol-4-yl)-4,5-dihydrothiazole* **(2l)**

Yield: 82%. m.p.: 112–114 °C. FT-IR ν (cm^−1^): 3098, 3068, 2949, 2856, 1627, 1583–1428. ^1^H NMR (400 MHz, CDCl_3_) δ 8.20 (s, H_5_), 8.07 (s, H_3_), 7.56 (dd, *J* = 8.0; 1.3 Hz, H_4_′), 7.50 (dd, *J* = 8.0; 1.3 Hz, H_6_′), 7.34 (t, *J* = 8.0 Hz, H_5_′), 4.39 (t, *J* = 8.2 Hz, H_4_″), 3.43 (t, *J* = 8.2 Hz, H_5_″). ^13^C NMR (100 MHz, CDCl_3_) δ 159.8, 140.7, 139.4, 134.7, 132.1, 130.8, 128.2, 127.9, 126.3, 118.9, 64.9, 34.1. HRMS (ESI) *m*/*z* [M+H]^+^ = 297.9964 (found), [M+H]^+^ = 297.9967 (calculated).

### 2.2. Cell Culture

Vero cells, obtained from the Rio de Janeiro Cell Bank (BCRJ code 0245), were dissociated with trypsin–EDTA solution (0.025%), and maintained in RPMI 1640 medium supplemented with 10% fetal bovine serum (FBS) at 37 °C in a humidified atmosphere of 5% CO_2_.

Tridimensional Vero cell cultures were obtained as previously described [31]. Briefly, the isolated cells (2 × 10^4^/well) were cultured in 96-well U-bottom plates previously coated with 1% agarose. The spheroids were kept for 5 days at 37 °C in RPMI 1640 medium supplemented with 10% FBS for complete three-dimensional organization. Both 2D and 3D culture models were used for trypanocidal activity assays.

### 2.3. Parasite

The screening assays were performed with *T. cruzi* clone Dm28c (TcI) genetically modified to express luciferase gene (Dm28c-Luc), kindly provided by Dr. Cristina Henriques [34,35]. Trypomastigotes were isolated from *T. cruzi*-infected Vero cell monolayers (10:1 parasites/host cell ratio). On the 4th day post-infection (dpi), free trypomastigotes in the culture supernatant were harvested, and the number of parasites/mL was quantified in a Neubauer chamber [36].

### 2.4. Cytotoxicity In Vitro Assay

The compound toxicity was evaluated in Vero cell cultures. Briefly, Vero cells were seeded in 96-well white culture plates at a density of 1.5 × 10^4^ cells/well for 24 h at 37 °C and 5% CO_2_. Thereafter, the cell monolayers were washed with phosphate-buffered saline (PBS) and 200 μL of fresh medium with a serial dilution of the pyrazole-thiazoline derivatives and Bz (1.9–500 µM) added. The cell viability was determined by luminescent assay using the CellTiter-Glo Kit (Promega Corporation, Madison, WI, USA) based on the ATP level [31,36]. After 72 h of treatment, 20 μL of CellTiter-Glo solution was added per well, and cultures were kept for 2 min under agitation. The luminescent signal was read on the GloMax reader (Promega Corporation, Madison, WI, USA). The concentration of compounds that reduced 50% of cell viability (CC_50_) was calculated by linear regression. At least three independent assays were performed in duplicate.

All compound stock solutions were prepared at 100 mM in dimethyl sulfoxide (DMSO). The final DMSO concentration for the controls and drug treatment did not exceed 1%.

### 2.5. Antiparasitic Assay

The trypanocidal activity of the pyrazole-thiazoline derivatives was evaluated against trypomastigote and intracellular amastigote forms of *T. cruzi* (Dm28c-Luc). Tissue-derived trypomastigotes (1 × 10^6^ parasites/well) were incubated with pyrazole-thiazoline derivatives and Bz in different concentrations (0.41–100 µM) for 24 h at 37 °C in a humidified atmosphere and 5% CO_2_. The parasite viability was determined by the addition of luciferin (300 µg/mL), followed by luminescence detection on the GloMax microplate reader (Promega Corporation, Madison, WI, USA) [31,36].

The susceptibility of intracellular amastigotes to pyrazole-thiazoline derivatives (series 1 and 2) was evaluated using 24 h *T. cruzi*-infected Vero cell monolayers (10:1 parasite/host cell ratio). Then, the cultures were washed with PBS to remove non-internalized parasites, and 200 μL of fresh medium with a serial dilution of pyrazole-thiazoline derivatives and Bz (0.41–100 µM) was added for 72 h at 37 °C.

After 72 h of treatment, luciferin (300 µg/mL) was added to the culture, and the viability of the parasites was assessed by reading the luminescent signal on the GloMax reader (Promega Corporation, Madison, WI, USA). Controls were also performed in non-toxic concentrations of DMSO (≤ 1%). The concentration that reduces the number of viable parasites by 50% (IC_50_) or 90% (IC_90_) was calculated by linear regression. The selectivity index (SI) was calculated as CC_50_/IC_50_ for each parasite form.

### 2.6. Reversibility Assays 

Vero cell cultures infected with *T. cruzi* Dm28c-Luc, as previously described, were incubated for 72 h at 37 °C, with the promising derivatives at concentrations higher than IC_90_ [31]. Then, the cultures were washed with PBS, and maintained for another 72 h at 37 °C in a drug-free medium. Trypomastigotes released in the culture supernatant were detected by transferring the culture medium to 96-well plates, followed by luciferin (300 µg/mL) addition. Viable intracellular amastigotes were also accessed by adding luciferin solution to the cell monolayers. The luminescence signal, revealing viable parasites, was read on the GloMax microplate reader (Promega Corporation, Madison, WI, USA).

### 2.7. 3D Culture Model

Vero cell spheroids were infected for 24 h with 2 × 10^5^ trypomastigotes (Dm28c-Luc). After washing, to remove the non-internalized parasites, the 3D cultures were treated for 72 h at 37 °C, with the promising derivatives in concentrations higher than IC_90_ [31]. Parasite load was assessed by the luminescence reaction after adding the luciferase substrate (luciferin 300 µg/mL). In addition, fluorescence microscopy images of untreated and treated *T. cruzi*-infected spheroids were acquired after fixing the cultures with 4% paraformaldehyde in PBS and 4′,6-diamidino-2-phenylindole (DAPI) staining, a DNA dye. The samples were observed under the Zeiss Axio Imager M2 fluorescence microscope (Carl Zeiss, Baden-Württemberg, Germany).

### 2.8. In Vitro Combination Therapy

In vitro combination treatment between promising candidates and Bz was performed against intracellular amastigotes (Dm28c-Luc) using a modified fixed-ratio isobologram method [37]. The highest concentrations of the compounds were determined by ensuring that the IC_50_ concentrations of the compounds in monotherapy were close to half of a series of eight serial dilutions (1:3). The first concentrations were prepared in the following ratios: 5:0, 5:1, 2:1, 1:2, 1:5, and 0:5 of Bz and promising candidates, respectively. Then, serial dilutions (1:3) of the proportions were made at eight points of dilution. Vero cell cultures infected with *T. cruzi* Dm28c-Luc were incubated with the compound combinations for 72 h. Two independent experiments in triplicate were performed. In each ratio, the IC_50_ of each compound was determined. The FICI (fractional inhibitory concentration index) of each IC_50_ was calculated as follows: IC_50_ when in combination/IC_50_ of compound alone. The FICI sum (ΣFICI) was determined as: FICI drug A + FICI drug B. xΣFICI is the mean of the FICI sums (FICI). The isobologram graph was built plotting the FICI for each proportion of the compounds. The xΣFICI was used to classify the interaction as synergistic xΣFICI ≤ 0.5, no interaction xΣFICI > 0.5–4, and antagonistic for xΣFICI > 4 [38,39].

### 2.9. Measurement of Reactive Oxygen Species (ROS) Levels

The production of intracellular ROS was evaluated in trypomastigotes (7 × 10^6^ parasites/well) treated for 1 h, 3 h, and 24 h at 37 °C, with promising derivatives in IC_50_ concentration. Then, the parasites were washed in PBS, and incubated for 30 min in the dark with 20 µM of 2′,7′-dichlorodihydrofluorescein diacetate (H_2_DCFDA). The fluorescence signal, generated by the oxidation of ROS sensitive probes, was measured using 485/535 nm excitation/emission wavelengths with a SpectraMax M2 microplate reader (Molecular Devices, San Jose, CA, USA). The fluorescence intensity of the blank was subtracted from all samples evaluated. Parasites incubated with 0.5% DMSO and 20 µM H_2_O_2_ were used as negative and positive controls, respectively [40].

### 2.10. In Silico Analysis

Physicochemical properties of promising candidates were calculated using OSIRIS DataWarrior software version 5.5.0 [41] and FAFDrugs4 [42]. The maps of molecular lipophilicity potential (MLP) were generated using Molinspiration Galaxy 3D Structure Generator v2021.01 beta [43]. DataWarrior was also used for the target search. First, molecules were retrieved within the ChEMBL database, annotated to target the *T. cruzi* parasite and proteins. Duplicity was removed, and then, scaffolds of pyrazole-thiazoline were queried against the small library of compounds. ADMET parameters (adsorption, distribution, metabolism, elimination, and toxicity) were acquired by inserting compounds’ molecular structures in the ADMETsar platform [44].

### 2.11. Proteinase Activity in Solution

Trypomastigote protein extracts (Dm28c-Luc; 10^8^ parasites/mL) were obtained as previously described [30]. The cysteine protease activity of the total protein extracts (5 µg) was measured using the reaction buffer (CH_3_COONa 10 mM, pH 5.0 containing 1 mM DTT), in the presence of the fluorogenic peptide substrate (60 μM of 7-Amino-4-Methylcoumarin hydrochloride), CBZ-L-Phenylalanyl-l-Arginine amide (Z -FR-AMC), and serial dilutions of pyrazole-thiazolines derivatives (100–6.25 µM). The samples were incubated for 45 min at room temperature. The variation in arbitrary fluorescence units (AFU), corresponding to the enzymatic cleavage of the fluorogenic substrate, was monitored in a SpectraMax M2e reader (Molecular Devices, Sunnyvale, CA, USA) for 1 h, with excitation values at 370 nm and emission values at 460 nm. Extracts incubated with 0.5% DMSO or with cysteine protease inhibitor E-64 were used as positive and negative controls, respectively. Substrate self-degradation was monitored, and did not produce significant effects. The inhibitory activity was expressed as percentage inhibition [31].

## 3. Results and Discussion

### 3.1. Chemistry

**Scheme 1:**
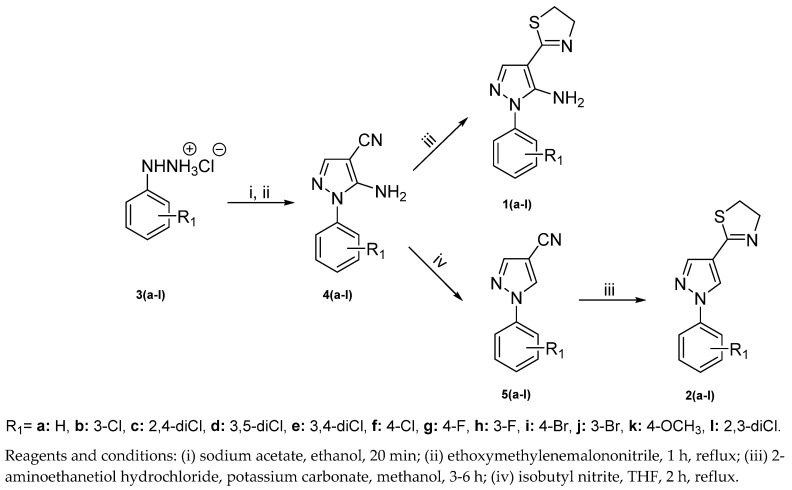
Synthetic routes to obtain **1(a-l)** and **2(a-l)**.

The synthetic routes to obtain the derivatives 2-(5-amino-1-aryl-1*H*-pyrazole-4-yl)-4,5-dihydrothiazoles **1(a-l)** and 2-(1-aryl-1*H*-pyrazole-4-yl)-4,5-dihydrothiazoles **2(a-l)** are summarized in Scheme 1. Firstly, commercial arylhydrazine hydrochlorides **3(a-l)** reacted with sodium acetate, through an acid–base reaction, in ethanol. Right after that, ethoxymethylenemalononitrile was added to afford the key intermediates 5-amino-1-aryl-1*H*-pyrazole-4-carbonitriles **4(a-l)** in a 69–98% yield. Subsequently, **4(a-l)** were submitted to an aprotic deamination reaction with isobutyl nitrite and tetrahydrofuran to obtain the desired 1-aryl-1*H*-pyrazole-4-carbonitriles **5(a-l)** in excellent yields (80–98%). Finally, the targets **1(a-l)** and **2(a-l)** were synthesized from **4(a-l)** and **5(a-l)**, respectively, through the reaction with 2-aminoethanethiol hydrochloride, potassium carbonate, and methanol as solvent. The compounds **1(a-l)** and **2(a-l)** were isolated in 76–94% and 71–91% yields, respectively. The structures of all of the newly synthesized derivatives, **1(a-l)** and **2(a-l)**, were confirmed by Fourier Transform Infrared (FTIR), ^1^H, and ^13^C Nuclear Magnetic Resonance (NMR), and High-Resolution Mass Spectrometry (HRMS) spectral analysis.

### 3.2. Toxicity and Phenotypic Screening of Pyrazole-Thiazoline Derivatives

The trypanocidal effect of the two new pyrazole-thiazoline series, **1(a-l)** and **2(a-l)**, was evaluated against clinically relevant forms of *T. cruzi* (intracellular amastigotes and trypomastigotes) using a luminescent viability assay (Table 1). Most series 1 derivatives (amino-pyrazole-thiazoline) have low activity against trypomastigotes, showing IC_50_ values ≥ 49.3 μM. From this series, only **1k** (IC_50_ = 12.0 ± 3.5 μM) is highlighted for exhibiting higher trypanocidal activity than Bz (IC_50_ = 19.6 ± 2.3 μM) (Table 1). However, **1k** showed a low effect against intracellular amastigotes, with an IC_50_ value of 79.1 ± 4.4 μM. Derivatives from series 1, **1(a-l)**, were not cytotoxic, with most analogues having a CC_50_ > 500 µM, but all compounds were less active than Bz (IC_50_ = 3.3 ± 1.1 µM) for intracellular amastigotes (Table 1).

Interestingly, the removal of the amino group (-NH_2_), giving rise to series 2, **2(a-l)**, increased the biological effect of the new derivatives, with some of them (**2b**, **2c**, and **2f**) exhibiting activity (IC_50_) in the nM concentrations range. In general, the series 2 activity against trypomastigotes had IC_50_ values < 10 µM, except for the **2d** (IC_50_ > 100) and **2l** (IC_50_ = 25.8 ± 1.9 µM) (Table 1). We highlight **2a** (IC_50_ = 1.1 ± 0.3 µM), **2b** (IC_50_ = 0.8 ± 0.3 µM), **2c** (IC_50_ = 0.4 ± 0.02 µM), **2e** (IC_50_ = 1.9 ± 0.4 µM), **2f** (IC_50_ = 0.6 ± 0.3 µM), **2i** (IC_50_ = 2.1 ± 0.9 µM), and **2k** (IC_50_ = 3.1 ± 0.3 µM) with trypanocidal activity 6.3 to 49-fold greater than Bz (IC_50_ = 19.6 ± 2.3 µM). For intracellular amastigotes, only **2c** (IC_50_ = 1.4 ± 0.4 µM) had better trypanocidal activity than Bz (IC_50_ = 3.3 ± 1.1 µM), reaching a selectivity index (SI) of 76.2 (Table 1). Some compounds, **2b**, **2c**, **2e**, **2f, 2h**, **2i**, and **2j**, reached IC_50_ < 10 µM, but had different SI values. None of the derivatives (series 1 and 2) showed an IC_90_ value lower than Bz (IC_90_ = 13.7 ± 4.2 µM) for intracellular amastigotes. Regarding cytotoxicity in Vero cells, series 2 has CC_50_ values ranging from 37.9 to 217.2 μM, except **2d** and **2e** (CC_50_ > 500 μM). Compounds **2c**, **2e**, and **2i** stand out for their high SI (>30) for both trypomastigote and amastigote forms of *T. cruzi* (Table 1).

### 3.3. Structure–Activity Relationship Analysis

In an attempt to potentiate the activity of the 5-aminopyrazole-imidazoline compound (hit compound), the imidazoline ring was replaced by thiazoline, and different substituents were inserted into the phenyl ring. Also, the amine group (-NH_2_) was introduced in series 1. In general, series 1 analogues showed lower potency (pIC_50_ < 4.95) than series 2, demonstrating that the polar group in the pyrazole ring of this new molecule had a negative effect on biological activity (Table 2). In series 1, the introduction of the substituent 2,3-diCl (**1l**) on the phenyl ring increased the potency (pIC_50_ = 4.95), whereas 2,4-diCl (**1c**), 3,4-diCl (**1e**), 4-Cl (**1f**), 3-F (**1h**), and 4-Br (**1i**) induced a drastic drop in potency (pIC50 < 4). 

Most series 2 derivatives have low micromolar activity. The insertion of chorine (**2b**, **2e**, and **2f**), fluorine (**2h**), or bromine (**2i** and **2j**) substituents resulted in compounds with similar pIC_50_ values, around 5.0 (Table 2). An attempt to move 4-F and 4-Br to the 3- position was not beneficial. Also, fluorine (**2g** and **2h**) and bromine (**2j**) substitutions increased toxicity to Vero cells. The addition of 4-F (**2g**), 4-OCH_3_ (**2k**), and 2,3-diCl (**2l**) decreased the potency (Table 2). The introduction of 3,5-diCl (**2d**) resulted in an even further decrease in potency (pIC_50_ < 4). The 2,4-diCl substituted analogue (**2c**) achieved the best potency (pIC_50_ = 5.85), showing a high selective index (SI = 76.2).

### 3.4. Physicochemical and ADMET Properties

**Figure 3:**
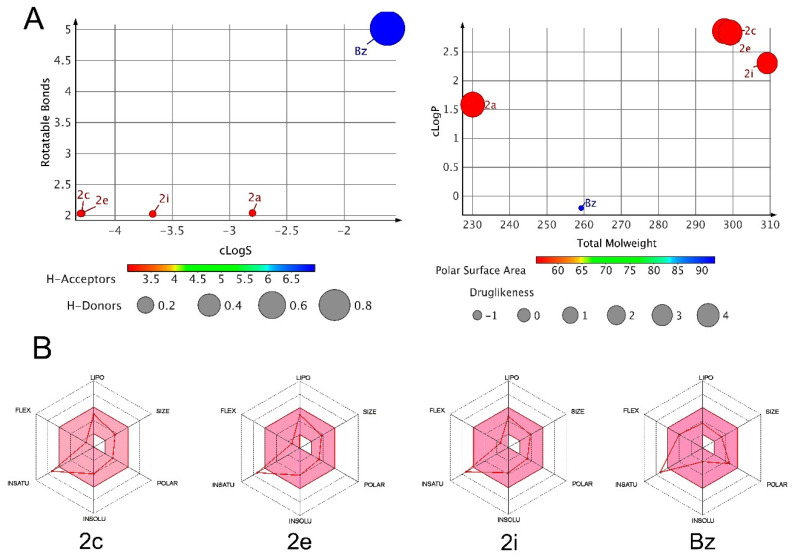
Physicochemical properties of promising pyrazole-thiazoline derivatives (**2c**, **2e**, **2i**) and Bz. (**A**) DataWarrior software was used to calculate and plot in 2D graphs the following physicochemical parameters: rotatable bonds, aqueous solubility (cLogS), hydrogen bond donors (HBD) and acceptors (HBA), lipophilicity (cLogP), molecular weight (MW), topological polar surface area (tPSA), and drug-likeness. (**B**) Radar plot representation of six physicochemical parameters (lipophilicity, size, polarity, solubility, flexibility, and saturation), calculated using SwissADME. In red, the threshold values to achieve the drug-likeness criteria.

The identification of three derivatives of series 2 (**2c**, **2e**, and **2i**) with potential trypanocidal activity, based on the IC_50_ < 10 µM and SI > 20 criteria, led us to assess whether the selected candidates had a good oral bioavailability prediction. The in silico analysis showed no violation of Lipinski’s rule of five (RO5), with molecular weight (MW) ranging from 308.20 to 298.19; a maximum lipophilicity (cLogP) value of 2.83; no more than 5 and 10 hydrogen bond donors (HBD) and acceptors, respectively; a reduced number of rotatable bonds (RB=2); and a topological polar surface area (TPSA) of 55.48 Å2 (Figure 3). Distinct physicochemical parameters were noticed between promising pyrazole-thiazoline derivatives and Bz (Figure 3). Bz revealed low lipophilicity, and a high number of rotatable bonds (RB = 5), and did not satisfy the drug-likeness criteria. The radar chat showed that all derivatives fit the drug-likeness criteria, except for the saturation values (sp3 carbon fraction <0.25), suggestive of good oral bioavailability (Figure 3).

**Figure 4:**
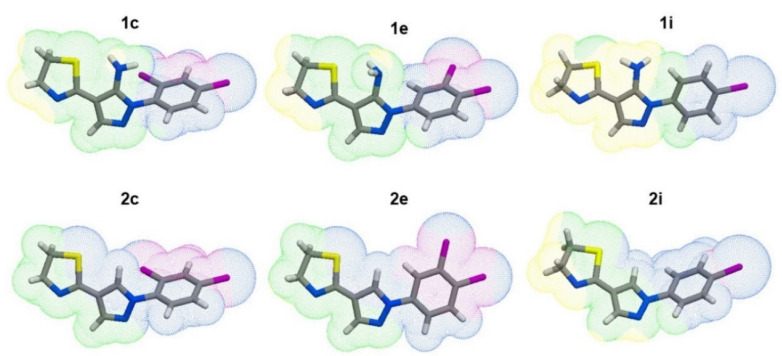
The molecular lipophilicity potential (MLP) of the promising pyrazole-thiazoline derivatives. Blue-violet color indicates the hydrophobic surface, intermediate lipophilic surfaces are coded by green, and yellow-orange colors represent the hydrophilic surface. Three-dimensional images were generated by Molinspiration Galaxy 3D Structure Generator v2018.01 beta (www.molinspiration.com, accessed on 10 December 2021).

Differences in the molecular lipophilicity potential (MLP) were observed between the pyrazole-thiazoline derivatives. Lipophilic regions coded with blue and violet color were mostly visualized in **2c** and **2e,** showing a prevalence of domains with a prediction of hydrophobic interactions which can favor the ligand–target binding (Figure 4). Interestingly, the polarity and lipophilicity are distinct between selected highly active compounds (**2c**, **2e**, and **2i**) and their lower active analogues (**1c**, **1e**, and **1i**), suggesting that, similar to cLogP, the distribution of hydrophilic and lipophilic domains in the molecular surface also interferes with the biological activity.

The in silico pharmacokinetic analysis of drug absorption revealed a prediction of diffusion across the blood–brain barrier (BBB), and good Caco-2 cell permeability and human intestinal (HIA) absorption (Supplementary Table S1). Pyrazole-thiazoline derivatives show a high probability to inhibit cytochrome P450 (CYP450) enzymes, such as CYP1A2, CYP2C19, and CYP2D6, with a prediction of low clearance. Most compounds showed a likelihood of hepatoxicity, but only **2e** exhibited mutagenicity prediction. None of the compounds are predictive of human ether-à-go-go-related gene (hERG) cardiac potassium channel inhibition. Thus, the favorable prediction of physicochemical and ADMET parameters motivated us to validate the trypanocidal effect using more complex biological systems.

### 3.5. Drug Efficacy on 3D Culture Model

**Figure 5:**
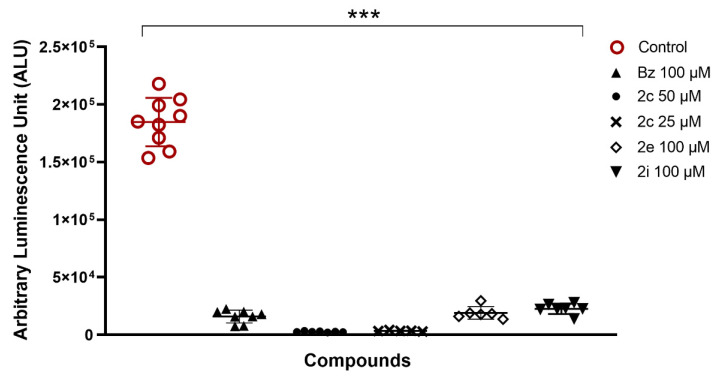
Effect of promising candidates, **2c**, **2e**, and **2i**, in *T. cruzi*-infected microtissue. Trypanocidal activity, represented as arbitrary luminescence unit (ALU), correlates to the luminescent signal proportional to the number of viable parasites (Dm28c-Luc). All compounds significantly decreased the parasite load in microtissue compared to control, showing a similar efficacy to Bz treatment. Statistical significance, in relation to the untreated group, was determined using the One-Way ANOVA test, with *p* < 0.001 (***).

The low success rate in the translation from in vitro biological activity into in vivo model efficacy led us to introduce the 3D culture model in our drug screening platform. Then, a 3D spheroids culture, which more accurately reflected human tissue complexity and the microenvironment, was employed to evaluate the efficacy of promising compound candidates. *T. cruzi*-infected 3D Vero cells were treated for 72 h with **2c**, **2e**, and **2i**, and parasite load was evaluated after luciferin addition. Parasite viability, assessed by luciferase activity, was determined by luminescence signal, represented as an arbitrary luminescence unit (ALU). A high parasite burden was observed in untreated spheroids (ALU = 166,678 ± 22,824), which was significantly reduced after treatment with all promising candidates (Figure 5). Potent activity was demonstrated by **2c** at concentrations of 25 (ALU = 3329 ± 250) and 50 µM (ALU = 1648 ± 343), resulting in approximately 98% inhibition compared to untreated *T. cruzi*-infected microtissue. Also, **2c** showed better efficacy even at lower concentrations (1.5–3 times IC_90_). Compounds **2e** (ALU = 15,110 ± 1990) and **2i** (ALU = 25,788 ± 2776) also demonstrated effectiveness in reducing parasite viability in *T. cruzi*-infected microtissue, similarly to Bz-treated spheroids (ALU = 20,024 ± 2796) (Figure 5).

**Figure 6:**
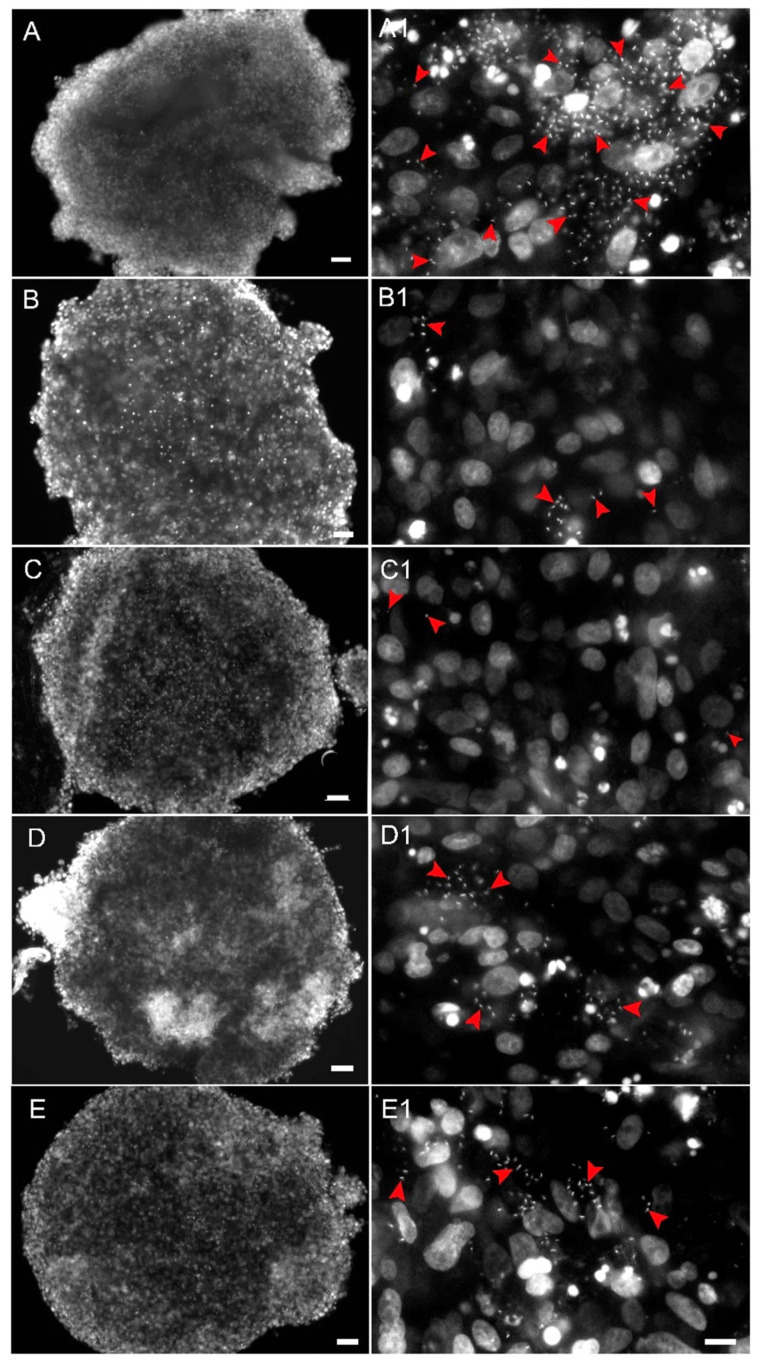
Analysis of **2c**, **2e**, and **2i** efficacy on *T. cruzi*-infected microtissues. (**A**–**E**) DAPI staining images of the whole infected spheroids untreated (**A**) and treated with Bz (**B**), **2c** (**C**), **2e** (**D**), and **2i** (**E**). (**A1**–**E1**) Representative areas of infected microtissues, highlighting the nucleus of the host cells, and the nucleus and kinetoplast of intracellular amastigotes (red arrowhead). (**A1**) Large amounts of intracellular amastigotes (red arrowhead) are visualized in *T. cruzi*-infected microtissue without treatment (control). (**B1**) Bz treatment (100 μM) showed a drastic reduction in the infection profile, with few intracellular parasites (red arrowhead). (**C1**) Treatment with **2c** (50 μM) showed a similar effect to Bz with rare parasites. Treatment with **2e** (**D1**) and **2i** (**E1**) at 100 μM concentration also revealed a reduction in parasite load. Bars: 50 μm (**A**–**E**) and 10 μm (**A1**–**E1**).

The ability of promising compounds to eliminate intracellular parasites was also analyzed by fluorescence microscopy. DAPI staining images revealed a high parasite load in infected and untreated microtissues (Figure 6). A large number of intracellular amastigotes was clearly visualized throughout the untreated spheroid, even in the innermost layers. In contrast, Bz-treated microtissues demonstrated a drastic reduction in the infection profile, with few intracellular parasites visualized in the spheroids (Figure 6). Treatment with **2c**, **2e**, and **2i** also impacted the parasite clearance. Few parasites were visualized in the spheroid treated with **2c**, indicative of good permeability and activity of the compound in the microtissue. All pyrazole-thiazoline derivatives markedly reduced the parasite load in the microtissue (Figure 6), as revealed by arbitrary luminescent data (Figure 5).

### 3.6. Potential of Pyrazole-Thiazoline Derivatives to Prevent Parasite Resurgence and the Effect in Combination Therapy

**Figure 7:**
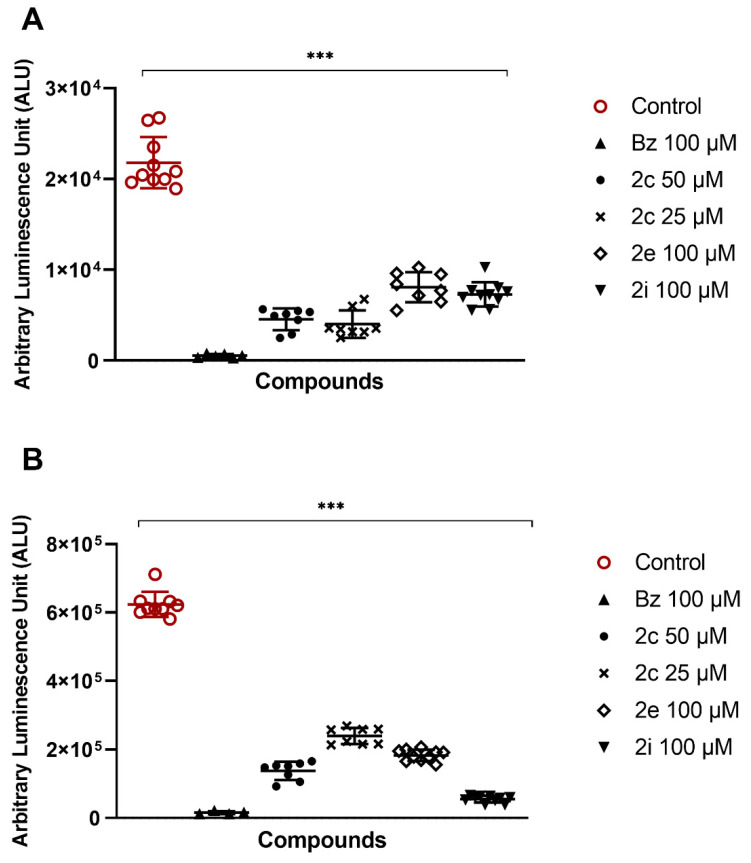
Analysis of **2c**, **2e**, and **2i** treatment on parasite recrudescence in vitro. After 72 h treatment, the compounds were removed, and after culture washing, drug-free medium was added for an additional 72 h culture. The luminescent signal, corresponding to parasite viability, was performed on both supernatant (**A**) and *T. cruzi*-infected Vero cell monolayers (**B**). Statistical significance, in relation to the untreated group, was determined using the One-Way ANOVA test, with *p* < 0.001 (***).

An important issue was to assess the compound activity reversibility. Then, the selected candidates (**2c**, **2e**, and **2i**) were evaluated for a prolonged period of time in order to assess their efficacy under drug-free conditions. Drug treatment was performed for 72 h at concentrations ranging from 1.5 to 7.2-fold the IC_90_ value, respecting their toxicity levels in mammalian cells (CC_50_). Most compounds were tested at 100 µM, except for **2c**, whose maximum concentration reached 50 µM. The trypanocidal effect was analyzed after drug washing and 3 days exposure in the absence of compounds. None of the pyrazole-thiazoline derivatives led to the suppression of parasitism, but all selected compounds significantly reduced the parasite load (Figure 7).

Bz treatment (100 µM) sustained the parasite clearance in both supernatant and cell monolayers. Compounds **2c** (50 µM) and **2i** (100 µM) were the most effective in controlling the intracellular amastigotes proliferation, inhibiting 78% and 89% of cell monolayer infection, respectively, compared to **2e** (69% inhibition) (Figure 7). Among the pyrazole-thiazoline derivatives, **2c** stands out for reducing by 85% the release of trypomastigotes in the culture supernatant (Figure 7). It is possible that prolonged treatment with the drug-containing medium replacement may improve the trypanocidal activity of promising candidates. It was shown that Bz treatment for 16 days of *T. cruzi*-infected Vero cell monolayers (strain Silvio X10/7) resulted in the prevention of spontaneous parasite relapse for up to 60 days, whereas posaconazole was unable to achieve parasite clearance [45]. 

**Figure 8:**
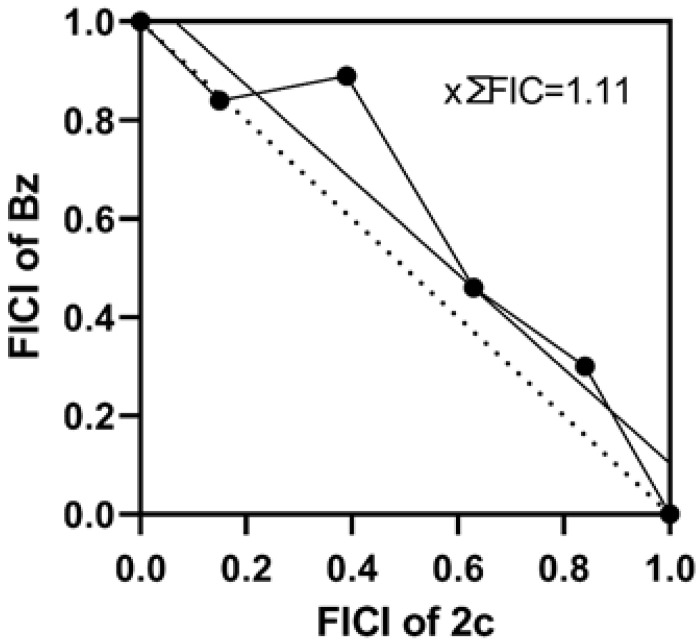
In vitro combinatorial activity of **2c** and Bz against the amastigote forms of *T. cruzi*. FICI values were calculated based on IC_50_ values of combinations. Isobologram graph was plotted with FICI values.

The **2c** showing better activity against trypomastigotes (IC_50_ = 0.4 µM) and intracellular amastigotes (IC_50_ = 1.4 µM), and even better potency (pIC_50_ = 5.85) than Bz (pIC_50_ = 5.48), motivated us to investigate whether the combination of **2c** and Bz could potentiate the trypanocidal activity. The in vitro effect of the Bz and **2c** combined treatment against intracellular amastigotes was analyzed by a luminescent assay, using *T. cruzi* Dm28c-Luc. The drug pairs were combined at four different ratios in accordance with the previously determined IC_50_ and FICI, ∑FICI, and x∑FICI values calculated. The x∑FICI value of 1.11 indicates no interaction between compounds **2c** and Bz in combined treatment (Figure 8). The isobologram, with ∑FICI values greater than 0.99 in all proportions, depicts the absence of an interaction in all proportions evaluated (Figure 8).

### 3.7. Drug Target and Mechanism of Action

**Figure 9:**
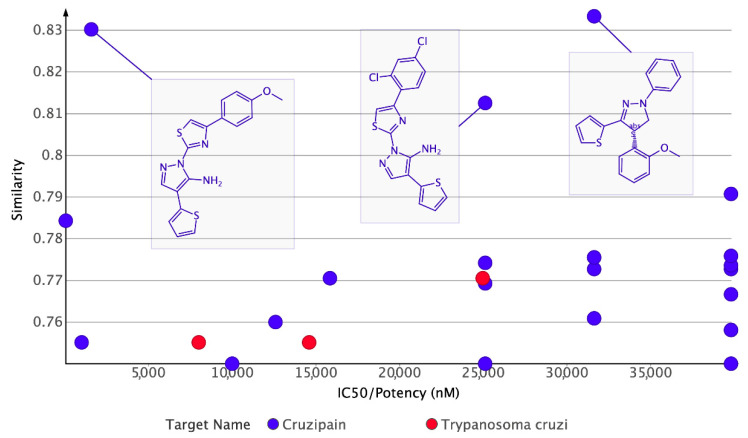
In silico analysis of pyrazole-thiazoline scaffolds using fragment-based search of molecular targets. The image shows matched scaffolds of pyrazole-thiazoline to trypanocidal compounds with different IC_50_ (nM) against annotated targets in the ChEMBL database. The analysis was performed using DataWarrior software, and three inhibitors of cruzipain were retrieved with similarity to pyrazole-thiazoline derivatives.

To evaluate if the insertion of the thiazoline group could change the pyrazole derivatives target that was previously identified as cruzipain, the compound scaffolds were queried in the ChEMBL database for similarity with different compounds with curated *T. cruzi* targets. One hit compound (IC_50_ < 10 µM) was similar (>0.8) to compound **2a**, and was annotated to target cruzipain (Figure 9). Despite similarity with **2a**, two other compounds had low activity against *T. cruzi*. 

**Figure 10:**
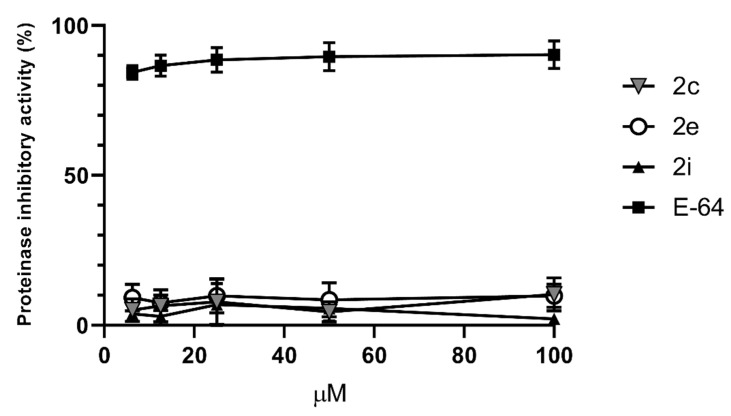
Analysis of the inhibitory activity of promising compounds on *T. cruzi* cysteine proteases. Protein extracts (5 µg) of trypomastigote forms of *T. cruzi* were incubated with a series of dilutions (100–6.25 µM) of pyrazole-thiazoline derivatives (**2c**, **2e**, and **2i**) and E-64, a specific inhibitor of cysteine proteases. The fluorescent signal resulting from the degradation of the fluorogenic substrate Z-FR-AMC (60 µM) was quantified to determine the enzymatic activity. Results are presented as mean and standard deviation (±) of percent (%) enzyme activity from at least three independent experiments.

The prediction of cruzipain as a potential target of promising pyrazole-thiazoline derivatives motivated us to investigate whether the ligand–target interaction would promote inhibition of cysteine protease activity. Thus, the enzymatic activity was measured in the total protein extract of trypomastigotes, using Z-FR-AMC as fluorogenic substrate. Incubation with promising compounds did not induce significant changes in enzymatic activity. Derivatives **2c**, **2e**, and **2i** showed a maximum inhibition profile of 10% of cysteine protease activity, even at high concentrations of the compounds (100 µM) (Figure 10). In contrast, E-64, a selective inhibitor of cysteine protease, inhibited the enzyme activity by more than 80% from the lowest concentration analyzed (6.25 µM). Thus, the replacement of imidazoline by thiazoline in 5-amino-1-aryl-4-(4,5-dihydro-1H-imidazol-2-yl)-1*H*-pyrazole (hit compound) did not favor binding with the active site of cruzipain, despite the prediction of an extensive hydrophobic domain associated with the phenyl ring containing 2,4- and 3,4-diCl, **2c** and **2e**, respectively (Figure 4), as previously evidenced in the hit compound [30].

**Figure 11:**
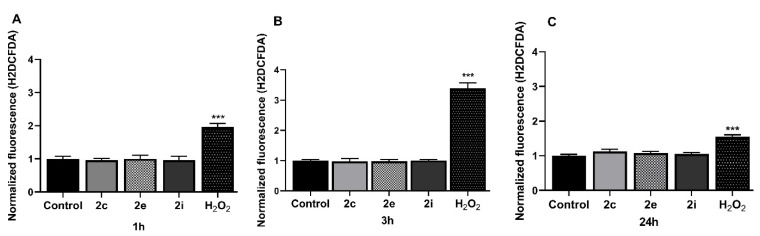
Analysis of reactive oxygen species (ROS) production in trypomastigote forms of *T. cruzi* treated with **2c**, **2e**, and **2i** for 1 h (**A**), 3 h (**B**), and 24 h (**C**) at IC_50_ concentrations of each compound. The H_2_DCFDA fluorescent probe was measured spectrofluorimetrically at 535 nm after excitation at 485 nm. Untreated and H_2_O_2_(20 µM)-treated parasites were used as negative and positive controls, respectively. Data are expressed as normalized values for the negative (untreated) control. Statistical significance, in relation to the untreated group, was determined using the One-Way ANOVA test, with *p* < 0.001 (***).

The failure to inhibit cysteine protease activity led us to assess whether the mechanism of action could be related to the induction of oxidative stress, since pyrazole derivatives have already been reported as iron-superoxide dismutase inhibitors [28,46]. Thus, the levels of reactive oxygen species (ROS) were evaluated using the 2, 7-dichlorofluorescein diacetate (H_2_DCFDA) probe. The intracellular ROS level in trypomastigotes was measured after 1, 3, and 24 h of compound treatment. None of the derivatives were able to induce redox imbalance in the IC_50_ concentration (Figure 11), indicating that another mechanism of action, not yet identified, is responsible for parasite death. Similar to Bz, which undergoes reductive metabolism to generate toxic intermediates that can act on redox metabolism, causes damage to DNA and RNA, and inhibits protein synthesis [47,48,49], the pyrazole-thiazoline derivatives or their metabolites may also act on multiple targets by interacting with proteins, or even forming adducts with nucleotides.

The induction of apoptosis through ROS production and the activation of caspase 3 by pyrazole derivatives has been reported in breast cancer cells, surpassing the activity of the first-line drug, paclitaxel [24]. However, a potent antioxidant activity has also been highlighted for pyrazole hybrids through increased antioxidant enzymes, and reduced lipid peroxidation [50].

## 4. Conclusions

A new series of twenty-four pyrazole-thiazoline derivatives were synthetized and analyzed against *Trypanosoma cruzi*. Three analogues, **2c**, **2e**, and **2i**, showed potent activity against trypomastigotes (IC_50_ ≤ 2.1 µM), and greater potency against intracellular amastigotes (pIC_50_ > 5.16). The promising candidates showed good pharmacokinetic prediction and drug-likeness properties. The SAR analysis highlights the improvement of trypanocidal activity after removal of the amino group, and the inclusion of halogen substituents, such as chlorine and bromine, in the *para* position of the phenyl ring. Despite the inability to sterilize infected cultures in the washout assay, the selected derivatives, **2c**, **2e**, and **2i**, showed improved potency and marked trypanocidal activity in the microtissue, encouraging the design of novel pyrazole-thiazoline scaffolds to advance in the drug discovery pipeline for Chagas disease.

## Figures and Tables

**Table 1:** Cytotoxicity and trypanocidal effect of pyrazole-thiazoline derivatives.

Compounds		Trypanocidal Activity (Mean ± SD μM)					Cytotoxicity (Mean ± SD μM)
	Trypomastigotes			Intracellular Amastigotes			Vero Cells
	IC_50_	IC_90_	SI	IC_50_	IC_90_	SI	CC_50_
**1a**	66.4 ± 2.7	>100	3.7	80.7 ± 3.9	>100	3.1	248.4 ± 22.0
**1b**	96.9 ± 3.1	>100	>5.2	95.7 ± 4.8	>100	5.2	>500
**1c**	>100	>100	<5	>100	>100	<5	>50
**1d**	>100	>100	>4.1	63.2 ± 5.4	>100	6.6	415.5 ± 34.6
**1e**	>100	>100	<5	>100	>100	<5	>500
**1f**	88.3 ± 7.1	>100	<5	>100	>100	<5	>500
**1g**	>100	>100	<5	96.3 ± 2.7	>100	5.6	483.0 ± 16.6
**1h**	>100	>100	<5	>100	>100	<5	>500
**1i**	49.3 ± 4.7	>100	>10.1	>100	>100	<5	>500
**1j**	52.3 ±3.4	>100	>9.5	26.6 ±3.1	>100	>18.7	>500
**1k**	12.5 ± 3.5	93.4 ± 5.5	>39.9	79.1 ± 4.4	97.3 ± 4.7	>6.3	>500
**1l**	>100	>100	<5	11.1 ± 2.0	>100	>44.9	>500
2a	1.1 ± 0.3	10.4 ± 1.1	166.8	10.7 ± 2.2	89.0 ± 2.6	17.4	187.3 ± 12.7
**2b**	0.8 ± 0.3	8.8 ± 1.3	100.2	8.3 ± 1.9	31.9 ± 1.3	9.4	78.3 ± 7.7
**2c**	0.4 ± 0.02	2.8 ± 0.6	269.9	1.4 ± 0.4	16.9 ± 2.5	76.2	106.7 ± 11.4
**2d**	>100	>100	<5	>100	>100	<5	>500
**2e**	1.9 ± 0.4	6.9 ± 2.0	>254	7.84 ± 1.0	30.4 ± 3.7	>62.9	>500
**2f**	0.6 ± 0.3	13.3 ± 2.3	143.6	7.6 ± 2.5	33.5 ± 4.4	12.0	91.7 ± 13.1
**2g**	7.9 ± 1.0	30.1 ± 2.8	7.3	14.0 ± 1.8	98.0 ± 3.4	4.18	58.6 ± 8.11
**2h**	9.8 ± 1.5	36.3 ± 3.2	3.8	9.1 ± 1.7	76.3 ± 6.1	4.19	37.9 ± 4.8
**2i**	2.1 ± 0.9	13.3 ± 2.8	101.2	6.8 ± 1.1	27.1 ± 3.6	31.8	217.2 ± 15.2
**2j**	9.1 ± 0.9	48.2 ± 4.1	7.11	8.1 ± 1.3	31.1 ± 3.1	7.9	64.5 ± 7.3
**2k**	3.1 ± 0.3	36.1 ± 2.6	36.1	22.4 ± 2.8	53.0 ± 9.8	4.9	110.1 ± 9.4
**2l**	25.8 ± 1.9	>100	3.44	25.3 ± 2.4	75.5 ± 8.3	3.5	88.7 ± 9.3
**Bz**	19.6 ± 2.3	95.9 ± 4.7	>18.9	3.3 ± 1.1	13.7 ± 4.2	>216	>500

Mean values of IC_50_ and IC_90_ from three independent experiments ± standard deviation (SD); IC_50_: concentration that inhibits parasite proliferation by 50%; CC_50_: concentration that reduces the viability of Vero cells by 50%; Nd = not determined; selectivity index (SI) = CC_50_ of Vero cells/IC_50_ of trypomastigote and intracellular amastigote forms of *T. cruzi.*

**Table 2:** Structure–activity relationship (SAR) analysis of pyrazole derivatives.

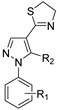 General Structure			
Compounds	R_1_	R_2_	Intracellular Amastigotes pIC_50_
**1a**	H	NH_2_	4.09
**1b**	3-Cl	NH_2_	4.01
**1c**	2,4-diCl	NH_2_	<4
**1d**	3,5-diCl	NH_2_	4.19
**1e**	3,4-diCl	NH_2_	<4
**1f**	4-Cl	NH_2_	<4
**1g**	4-F	NH_2_	4.01
**1h**	3-F	NH_2_	<4
**1i**	4-Br	NH_2_	<4
**1j**	3-Br	NH_2_	4.57
**1k**	4-OCH_3_	NH_2_	4.10
1l	2,3-diCl	NH_2_	4.95
**2a**	H	H	4.97
**2b**	3-Cl	H	5.08
**2c**	2,4-diCl	H	5.85
**2d**	3,5-diCl	H	<4
**2e**	3,4-diCl	H	5.10
**2f**	4-Cl	H	5.11
**2g**	4-F	H	4.85
**2h**	3-F	H	5.04
**2i**	4-Br	H	5.16
**2j**	3-Br	H	5.09
**2k**	4-OCH_3_	H	4.64
**2l**	2,3-diCl	H	4.59
**Bz**	-	-	5.48

The effects of modifications in the R-groups of pyrazole-thiazoline derivatives are expressed as inhibitory potency values (pIC_50_ = −log IC_50_) against amastigote forms of *T. cruzi* (Dm28c-Luc clone). Higher values of pIC_50_ indicate exponentially more potent inhibitors.

## Data Availability

Not applicable.

## Supplementary Materials

The following supporting information can be downloaded at: https://www.mdpi.com/article/10.3390/pharmaceutics14050995/s1, Figure S1: NMR spectra of the compounds; Table S1: ADMET properties.
