# Supplementary material for: Bioactivity of Novel Pyrazole-Thiazolines Scaffolds against Trypanosoma cruzi: Computational Approaches and 3D Spheroid Model on Drug Discovery for Chagas Disease
Source: Pharmaceutics. 2022 May 5;14(5):995. doi: 10.3390/pharmaceutics14050995 (PMC9146228; doi:10.3390/pharmaceutics14050995)

^{1}H NMR of compound **1a**

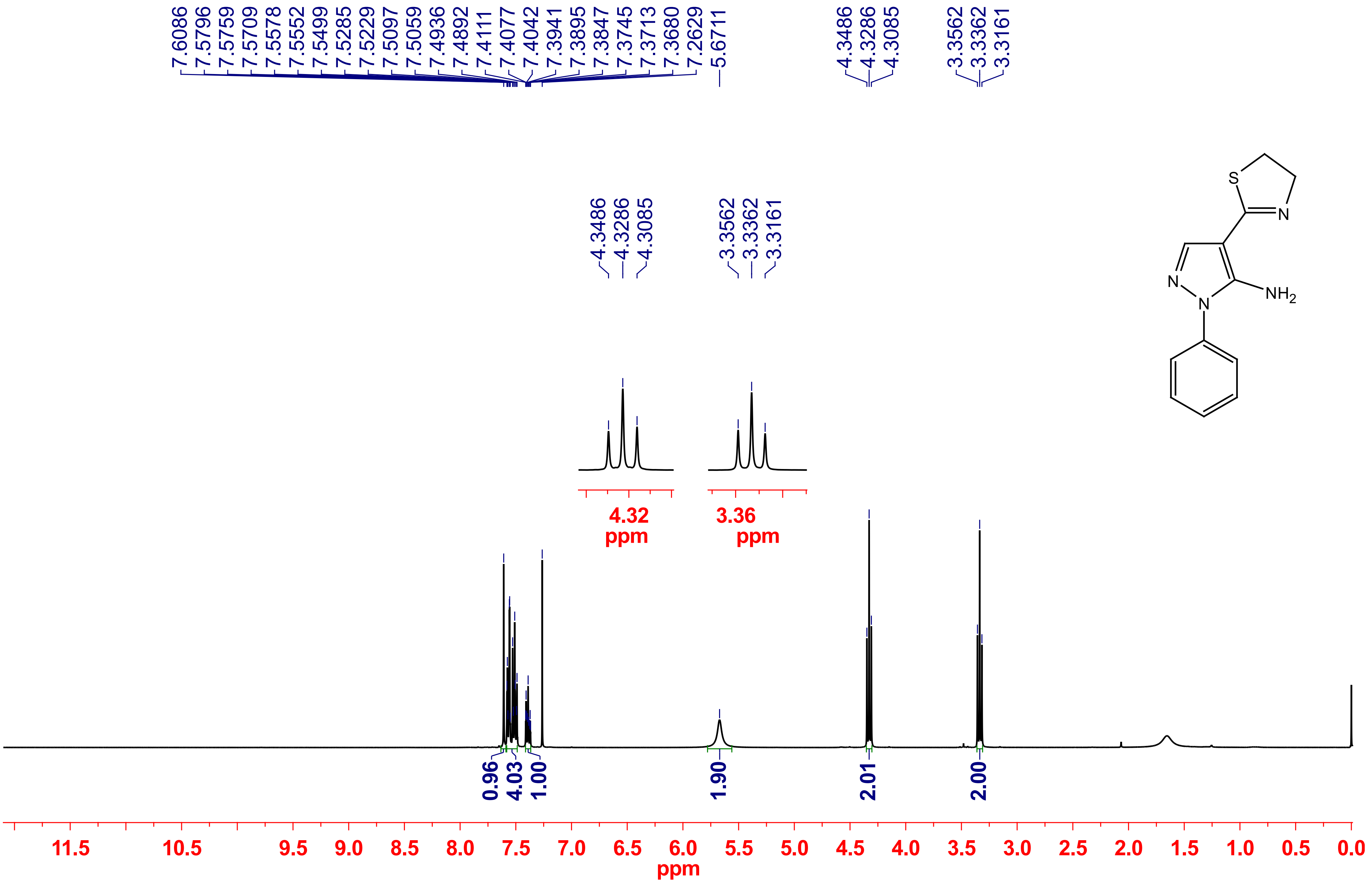

^{13}C NMR of compound **1a**

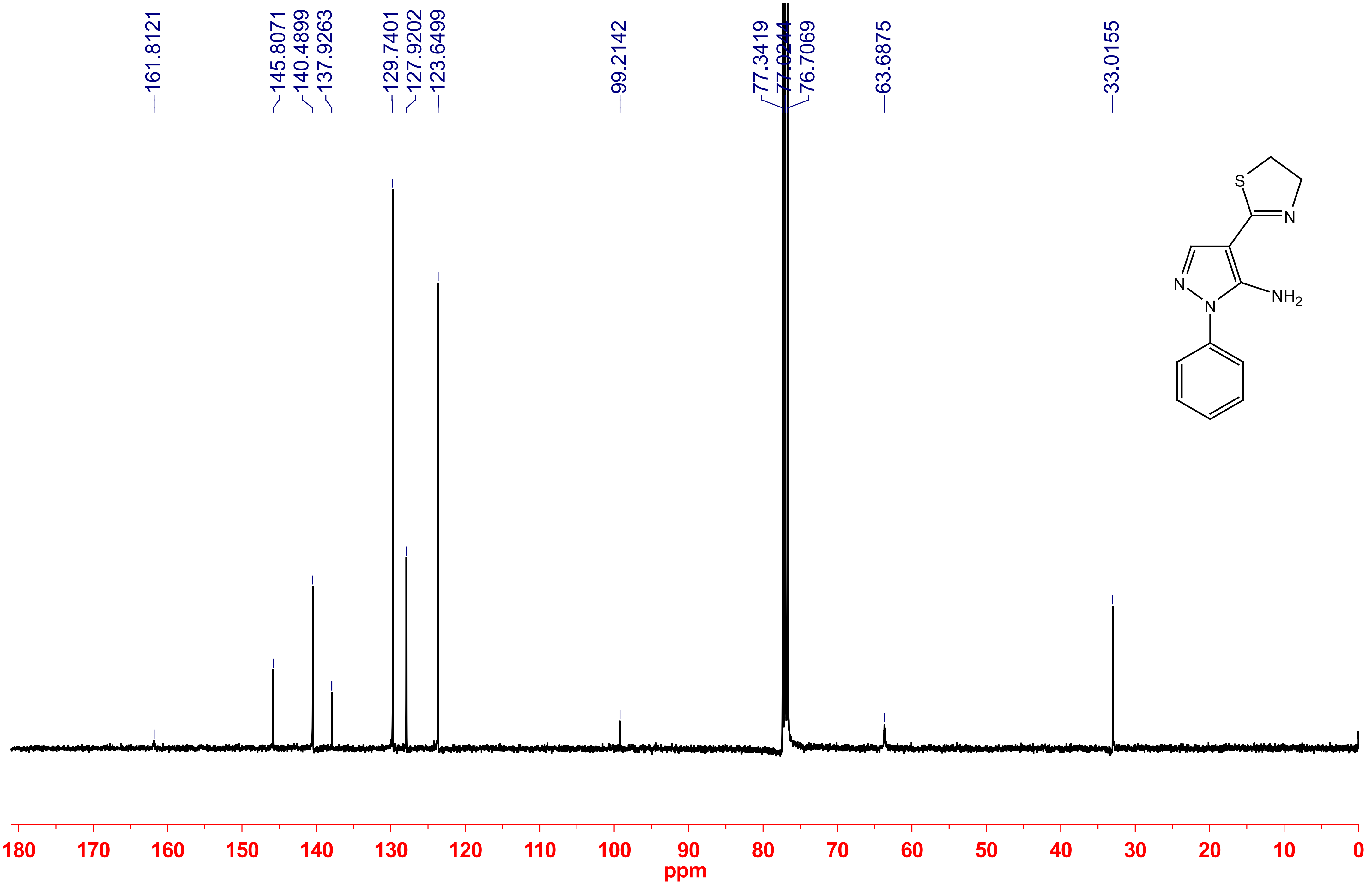

^{1}H NMR of compound **1b**

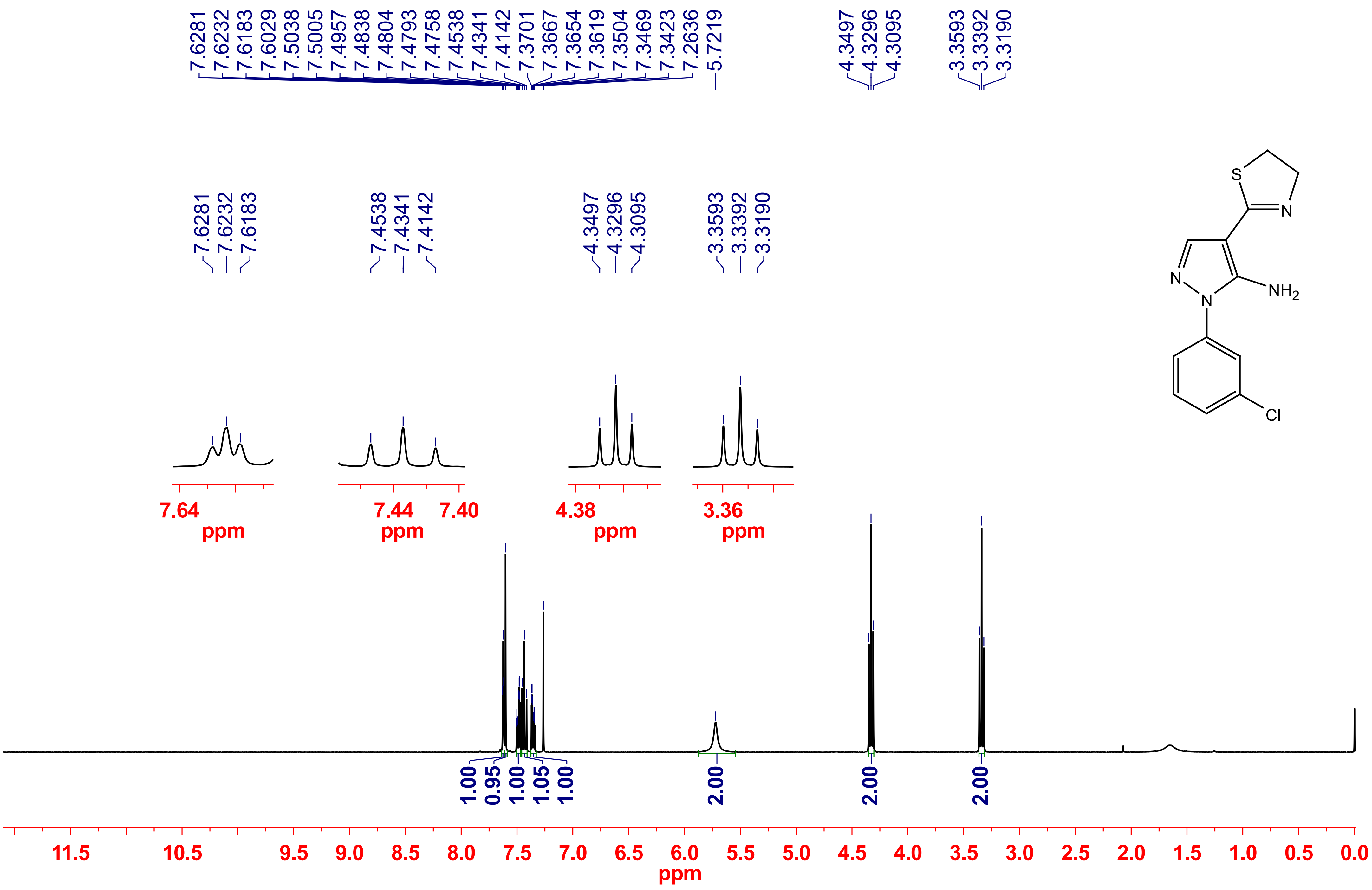

^{13}C NMR of compound **1b**

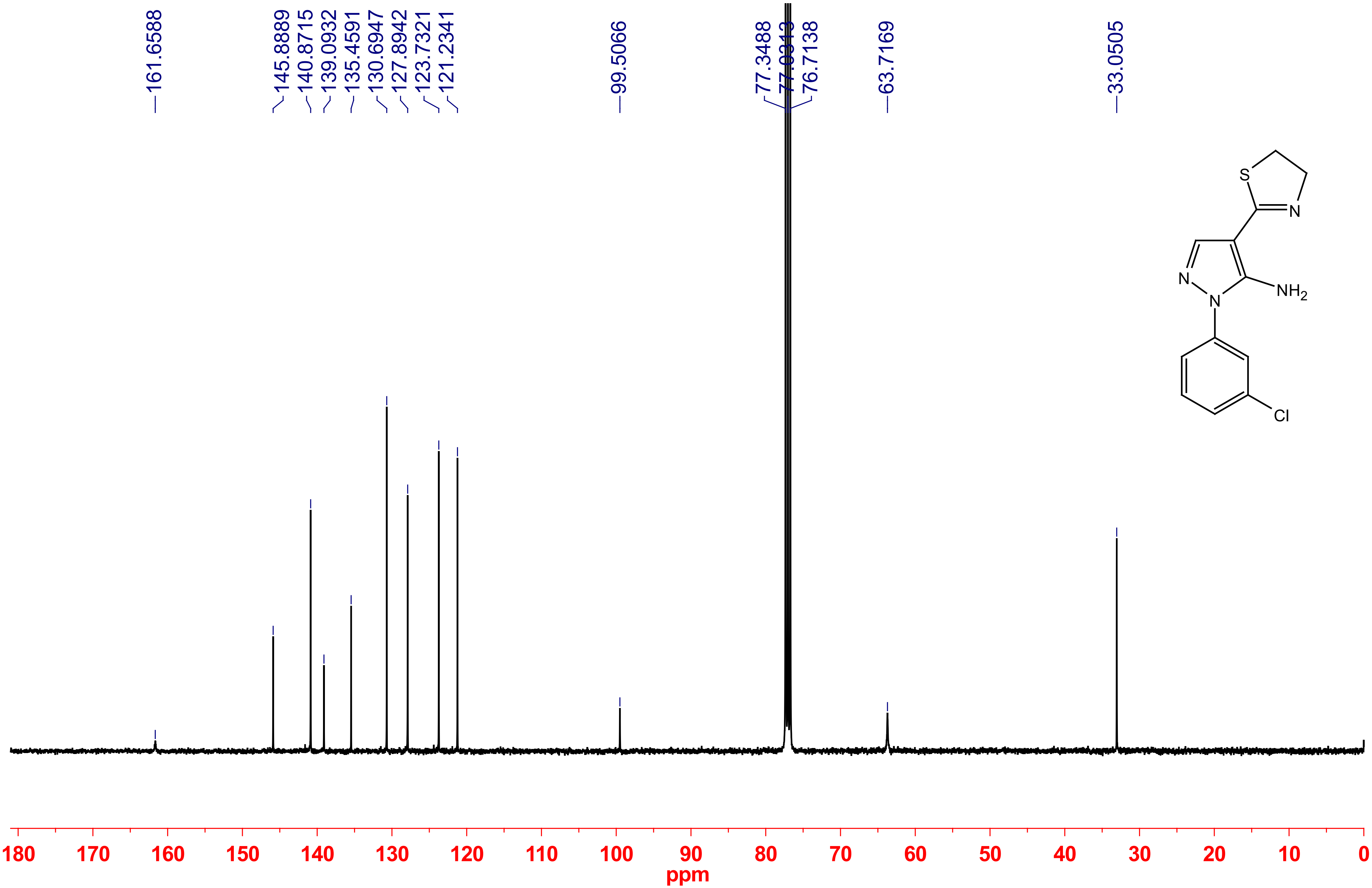

^{1}H NMR of compound **1c**

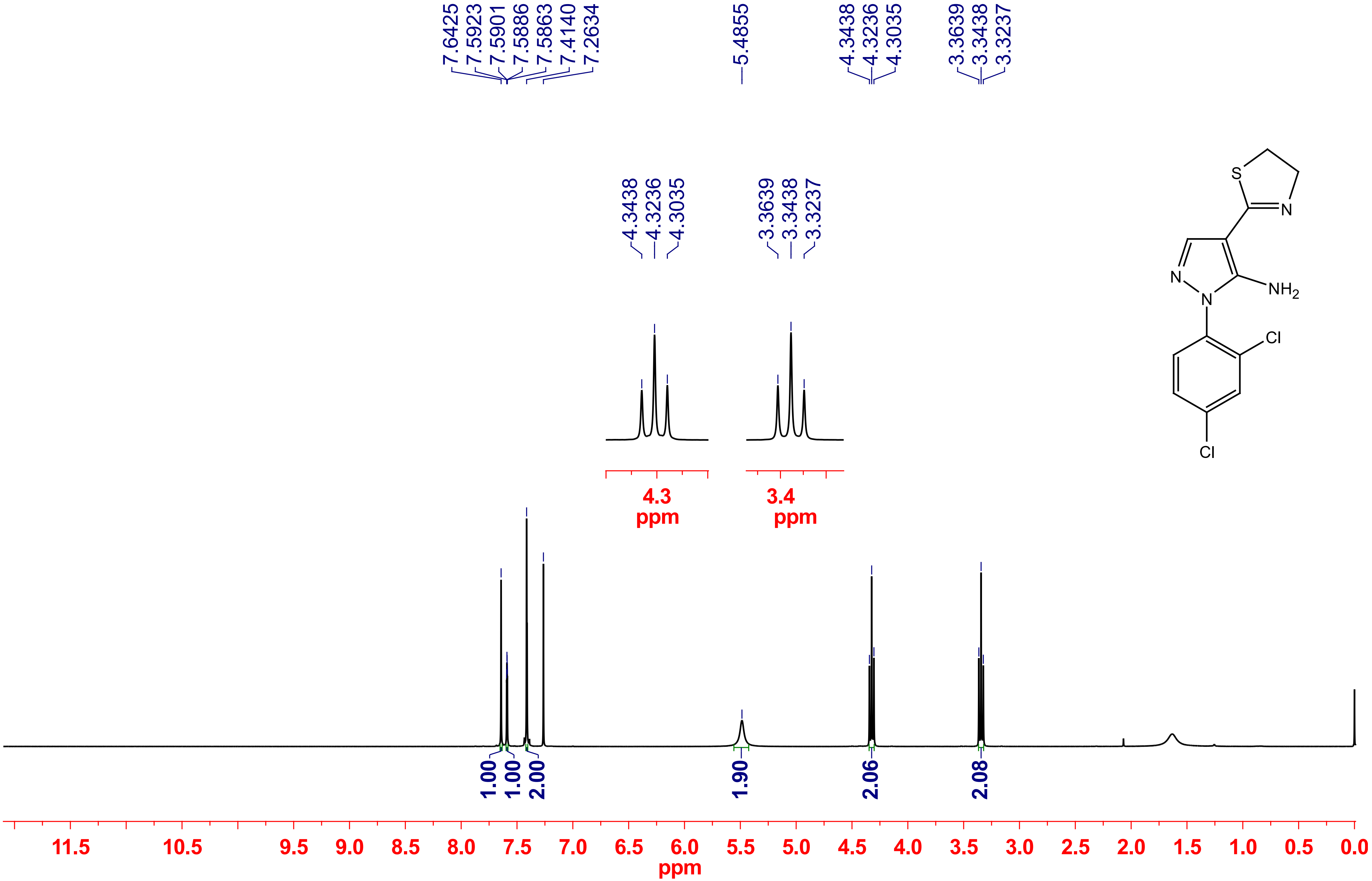

^{13}C NMR of compound **1c**

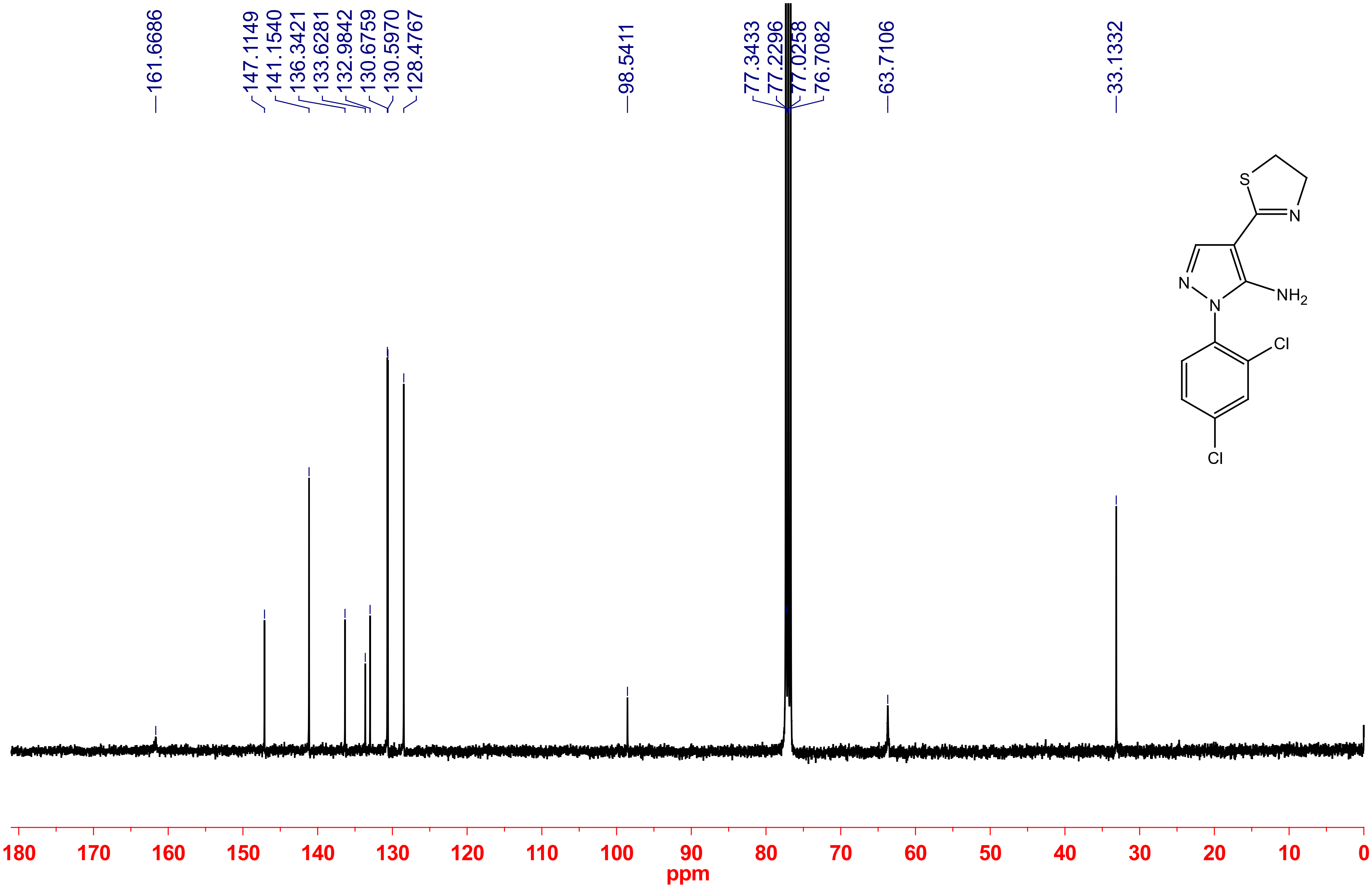

^{1}H NMR of compound **1d**

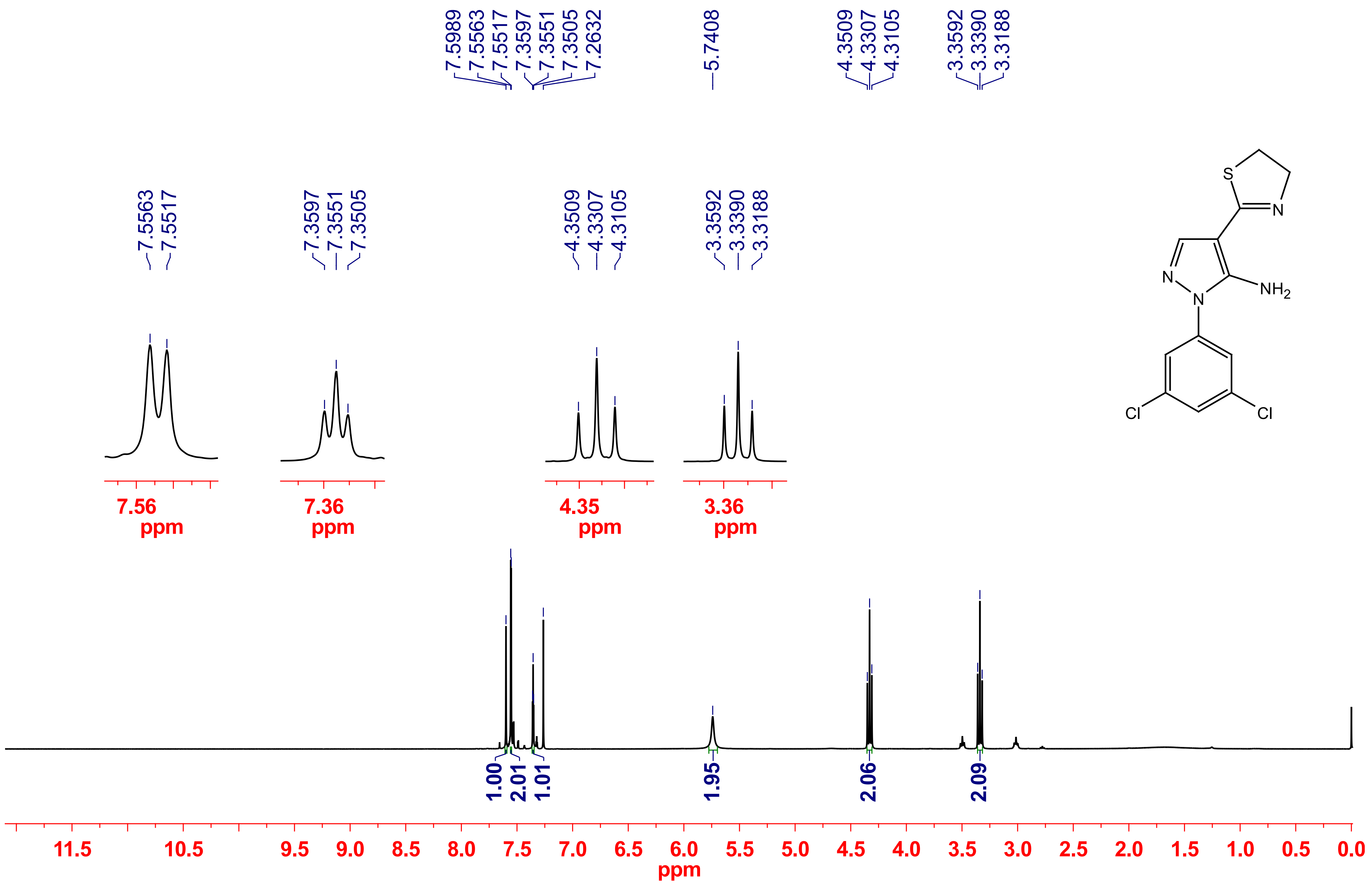

^{13}C NMR of compound **1d**

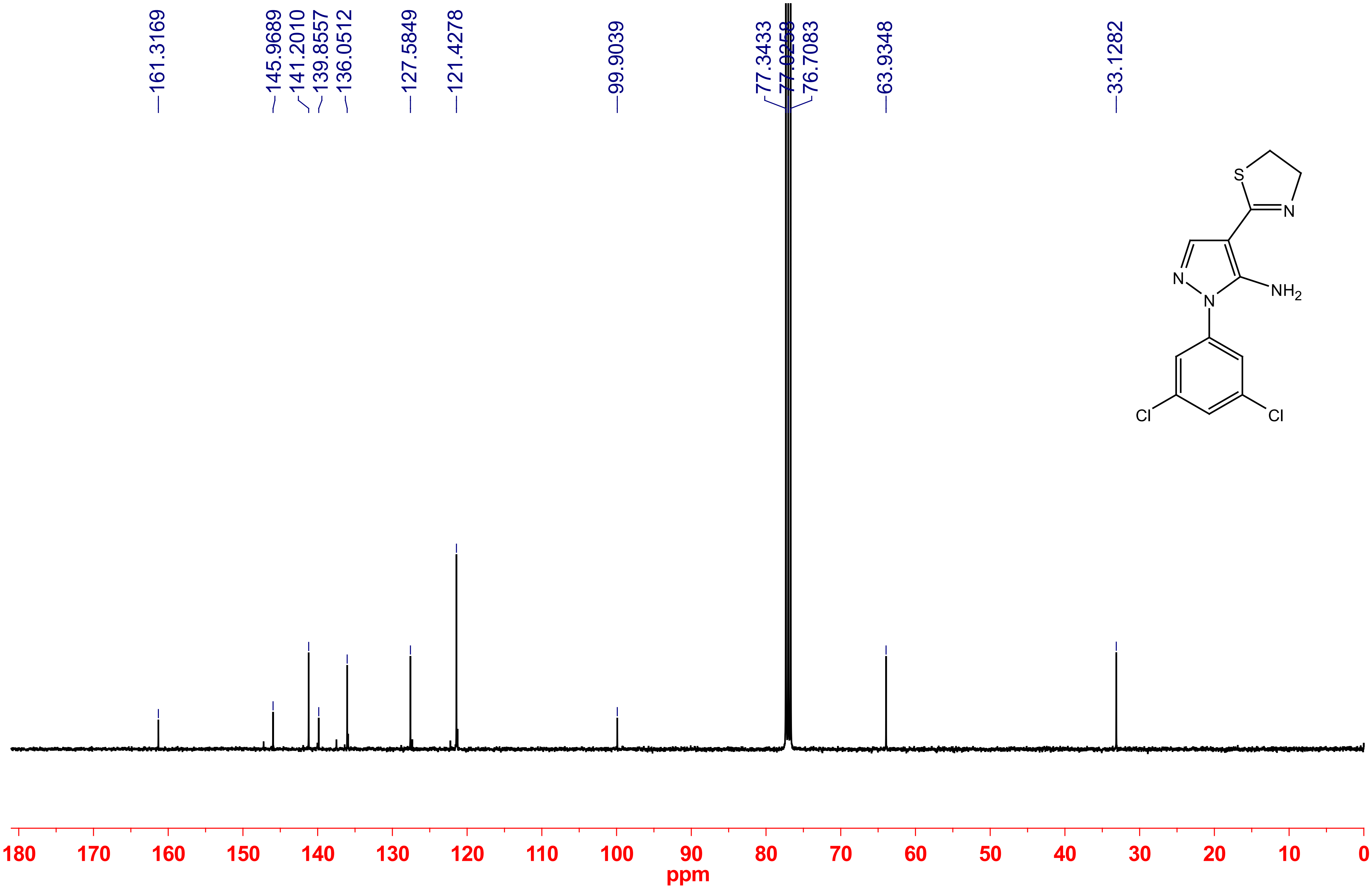

^{1}H NMR of compound **1e**

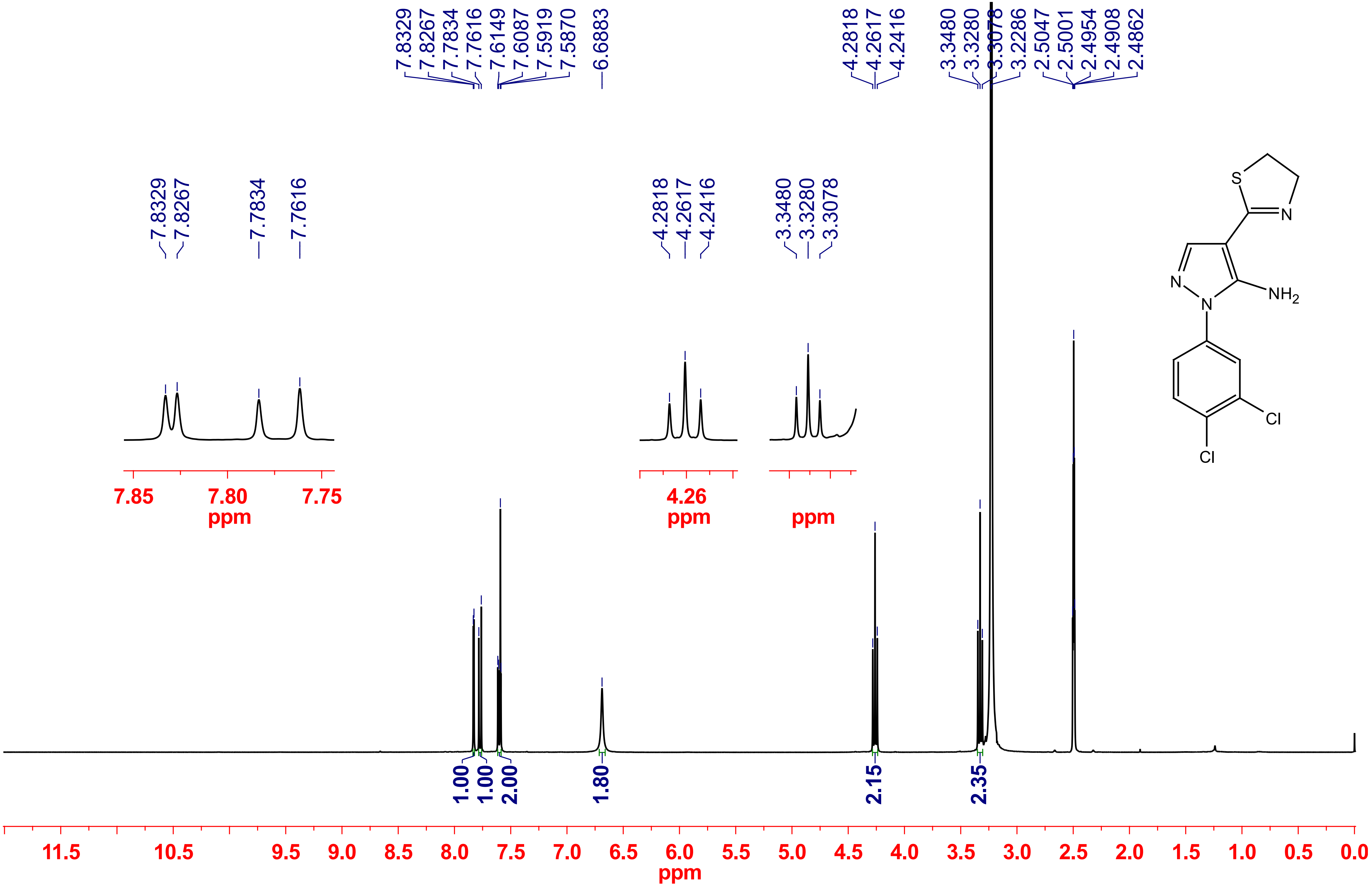

^{13}C NMR of compound **1e**

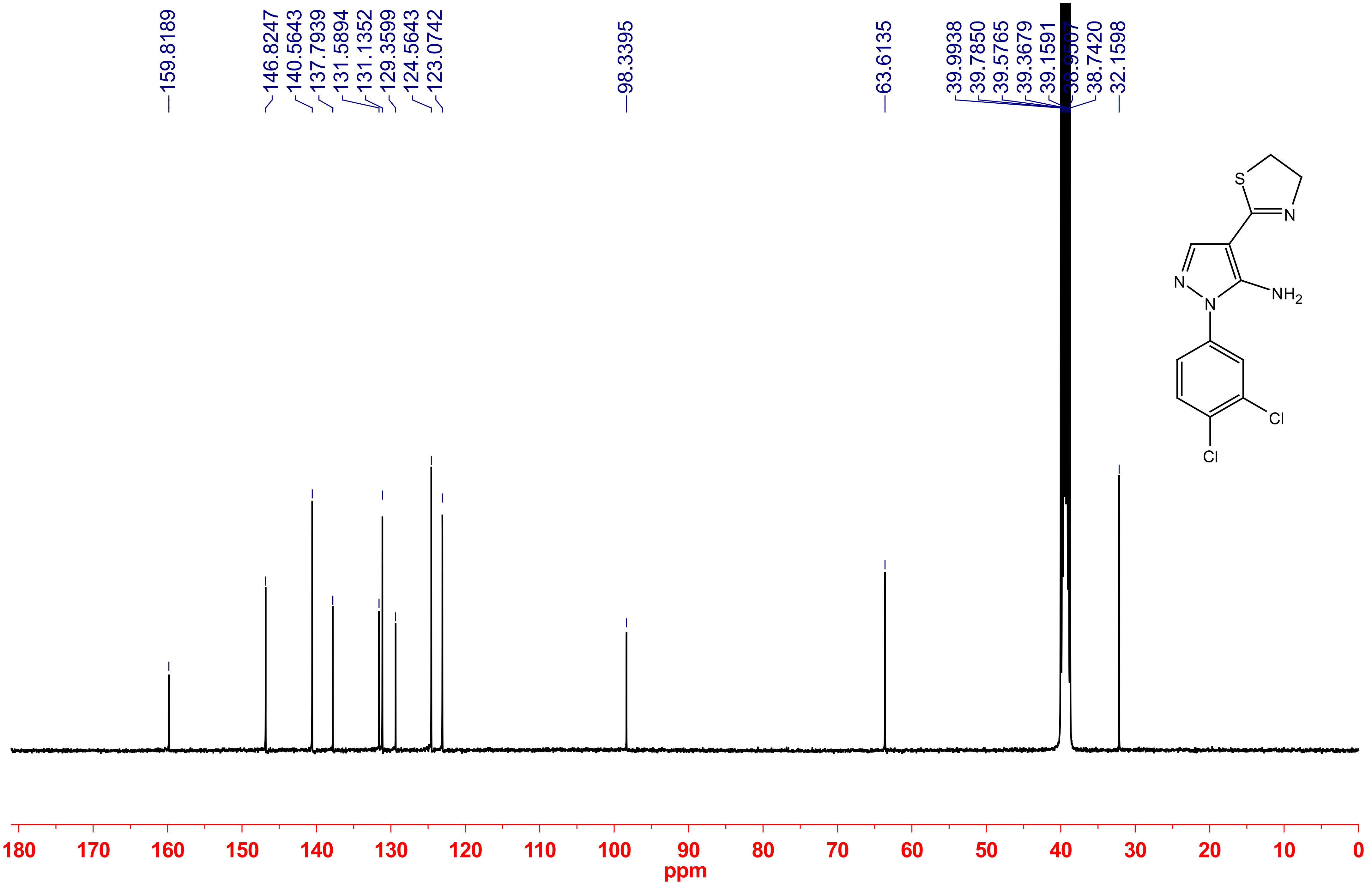

^{1}H NMR of compound **1f**

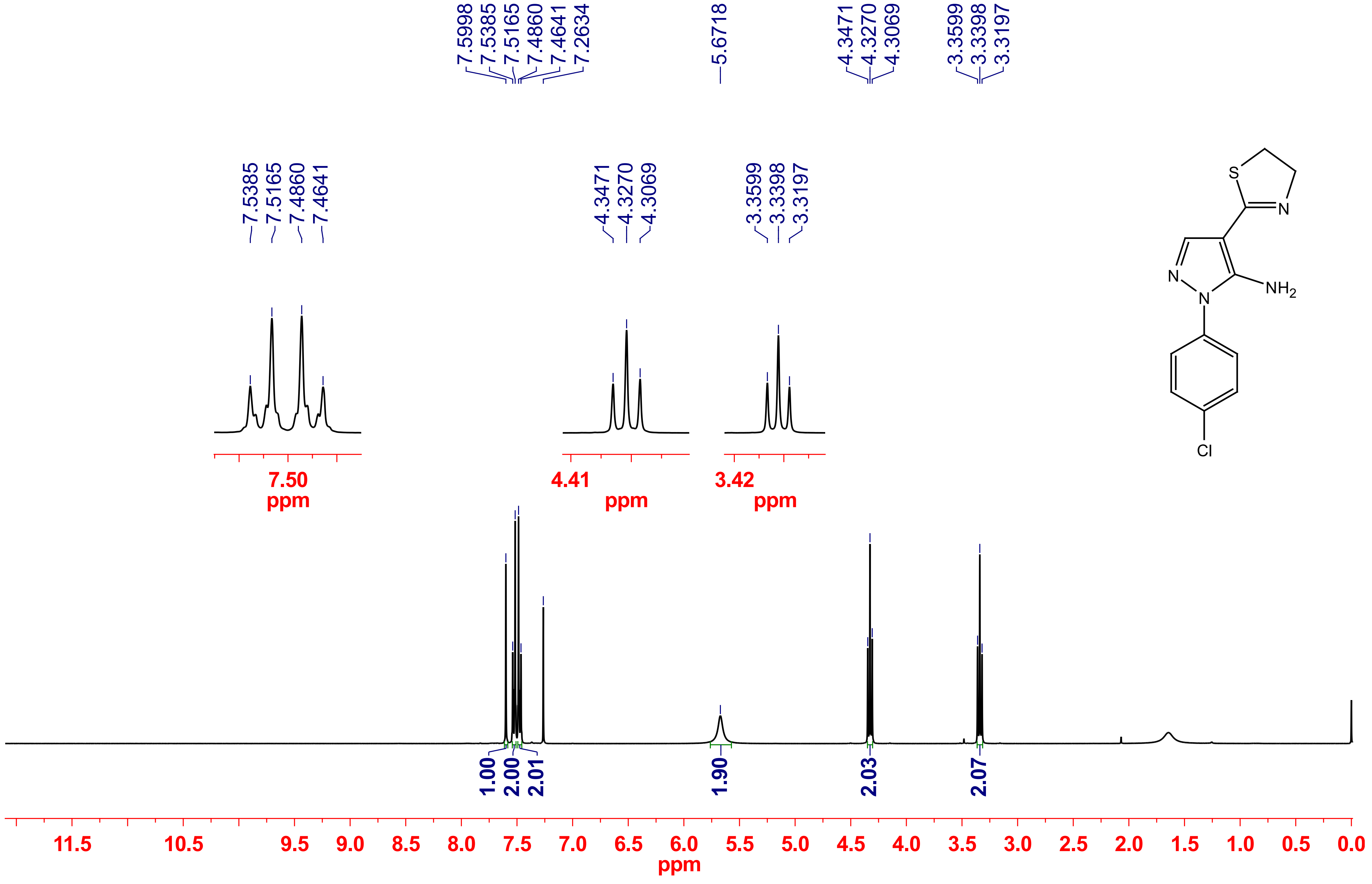

^{13}C NMR of compound **1f**

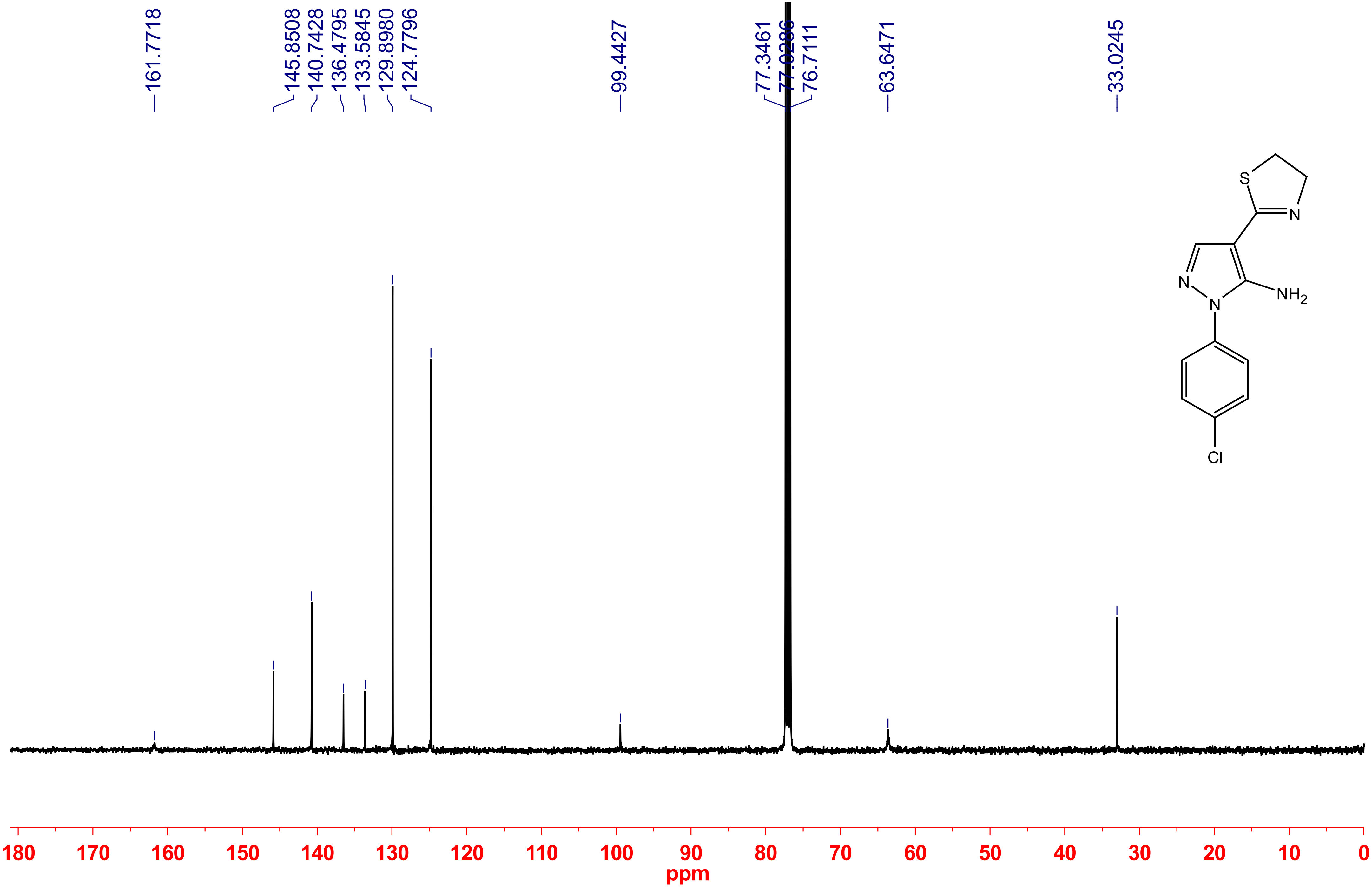

^{1}H NMR of compound **1g**

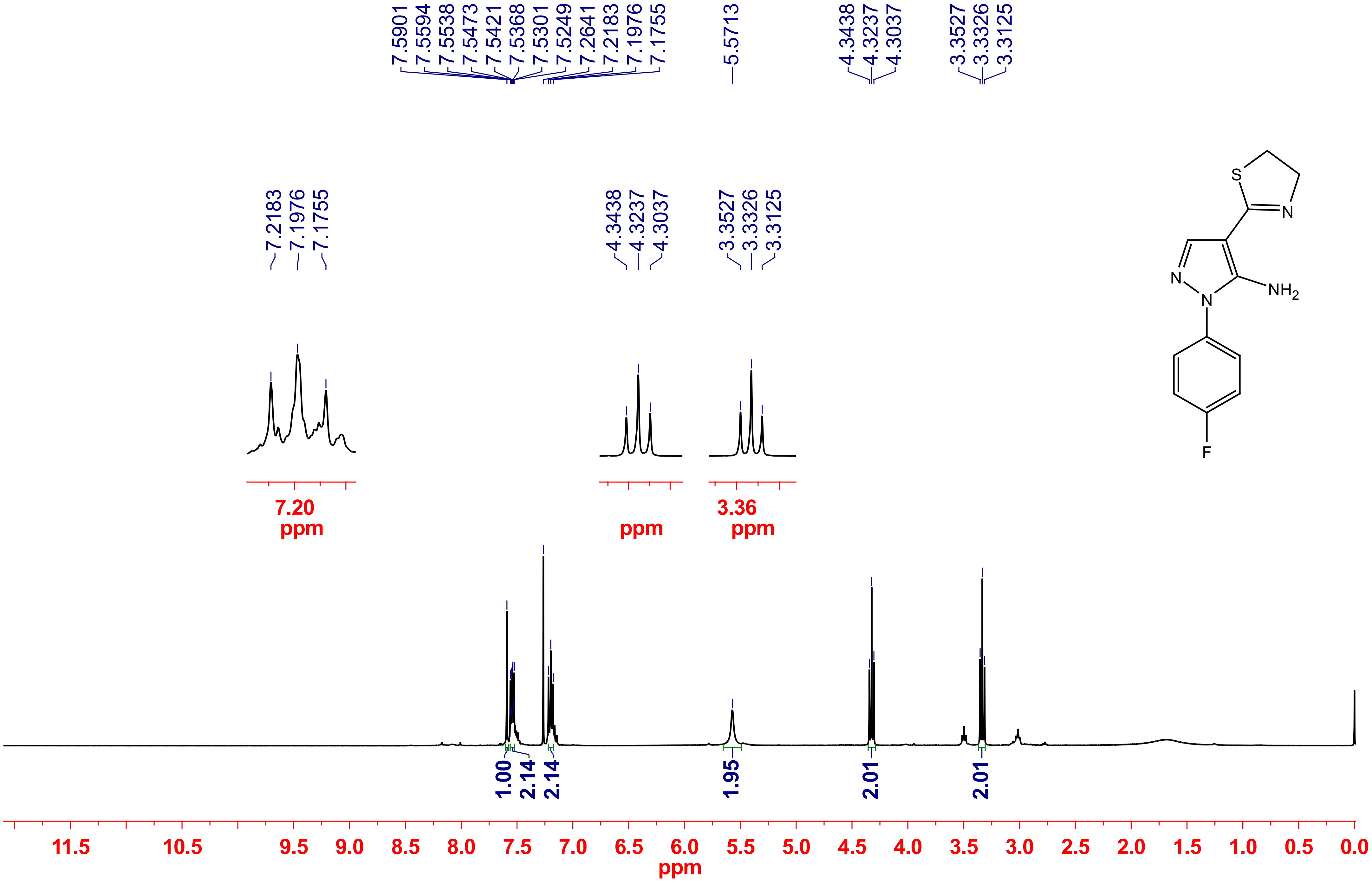

^{13}C NMR of compound **1g**

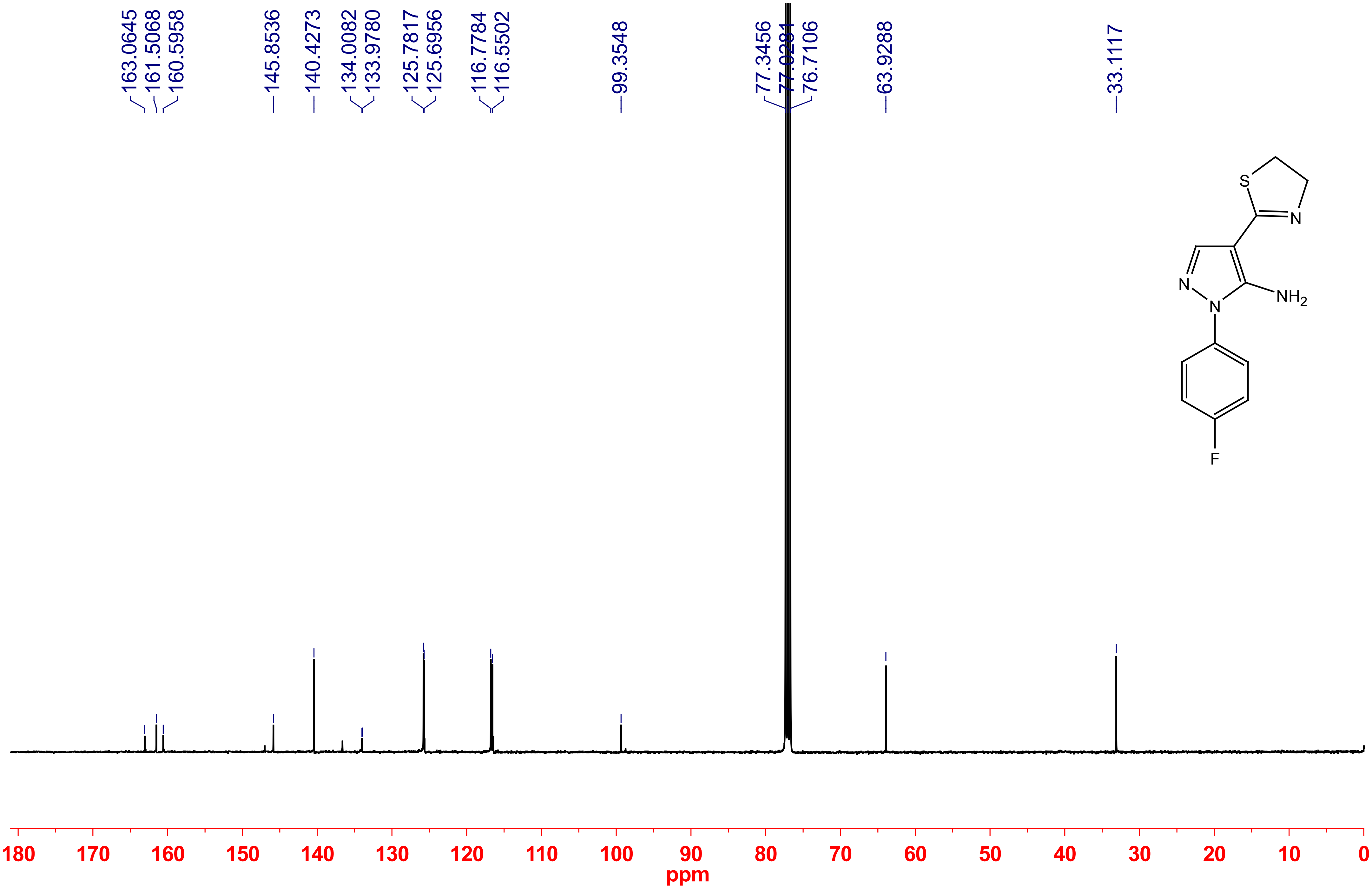

[1] H NMR of compound **1h**

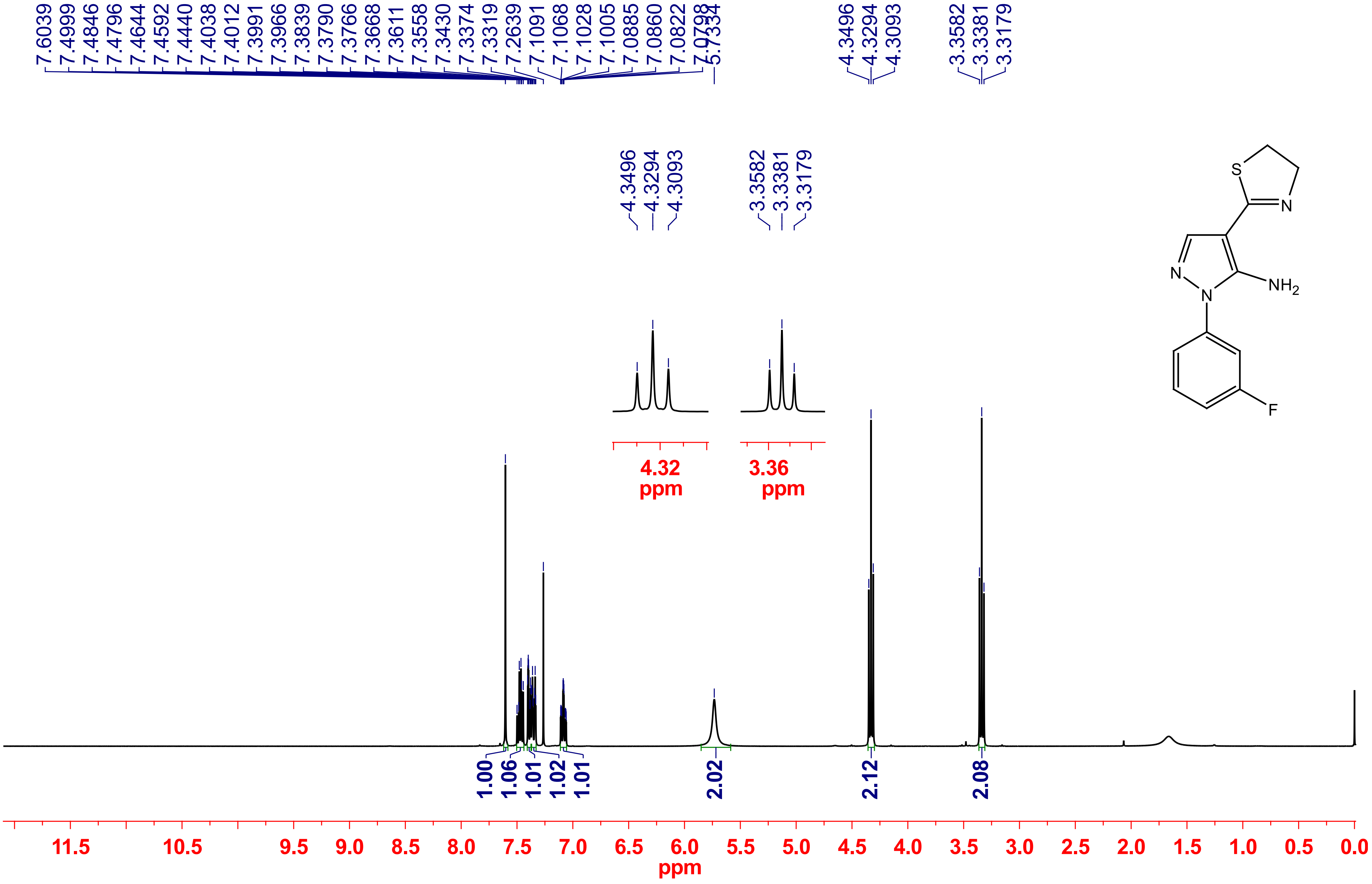

^{13}C NMR of compound **1h**

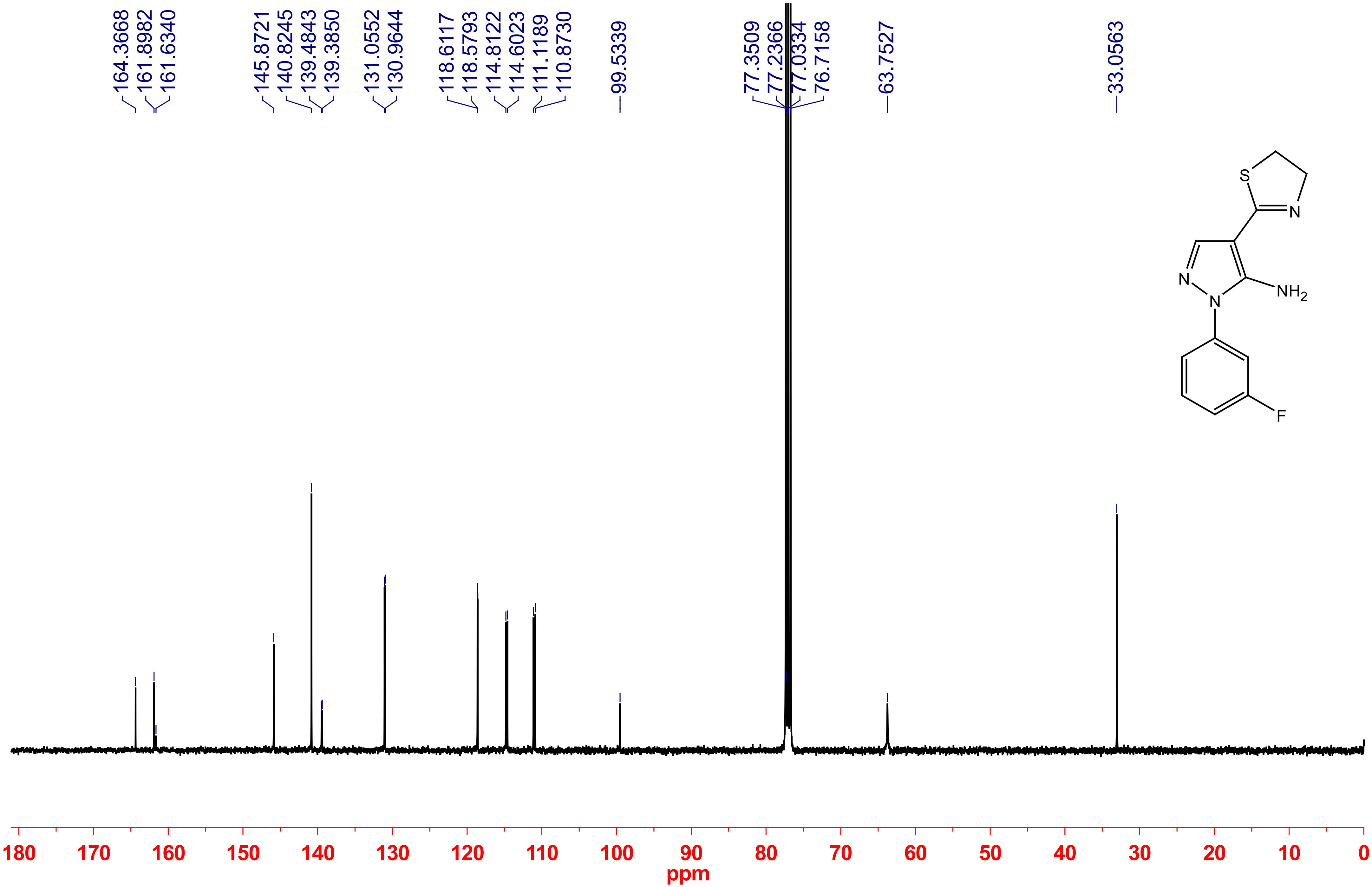

^{1}H NMR of compound **1i**

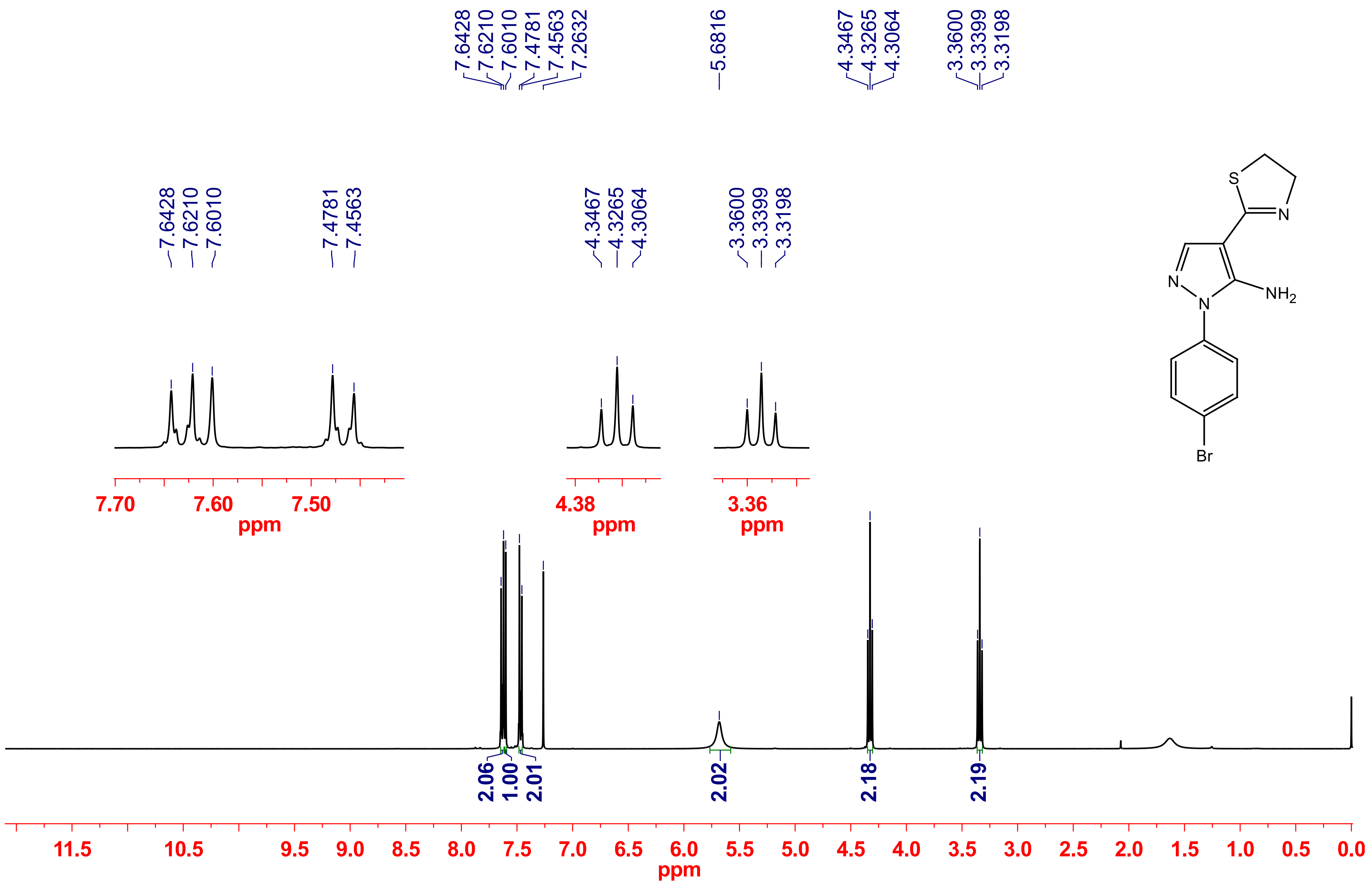

^{13}C NMR of compound **1i**

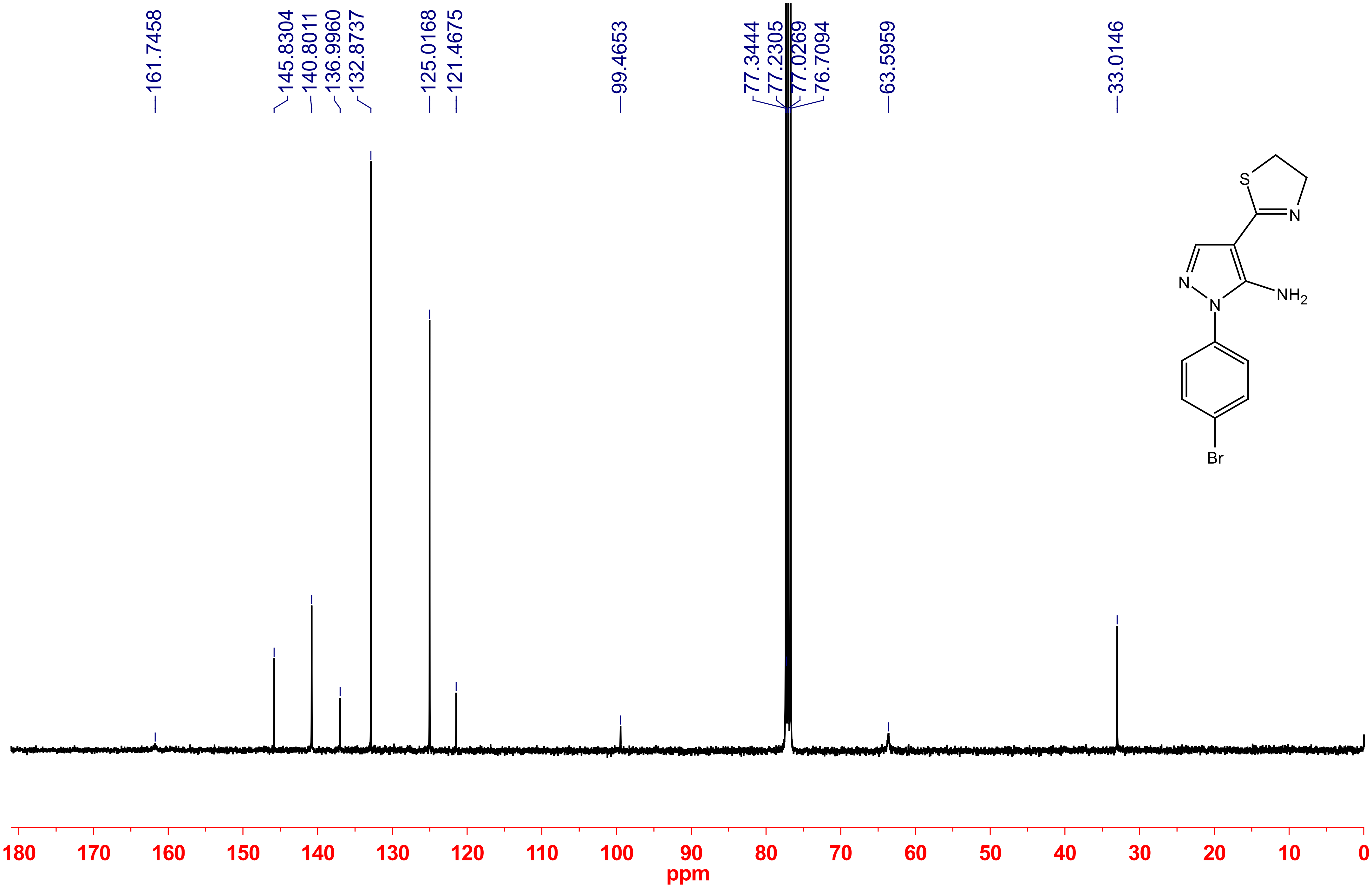

^{1}H NMR of compound **1j**

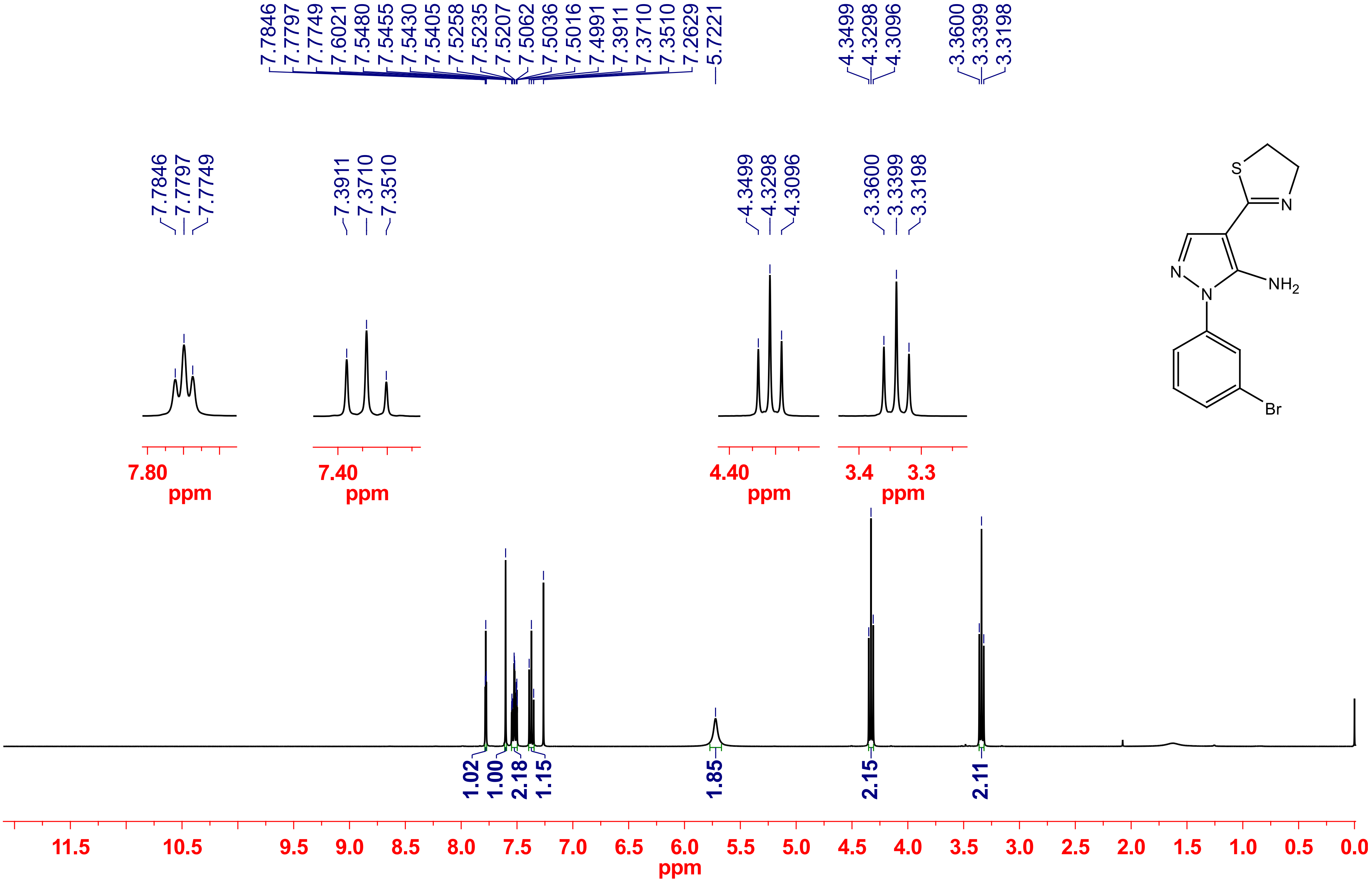

^{13}C NMR of compound **1j**

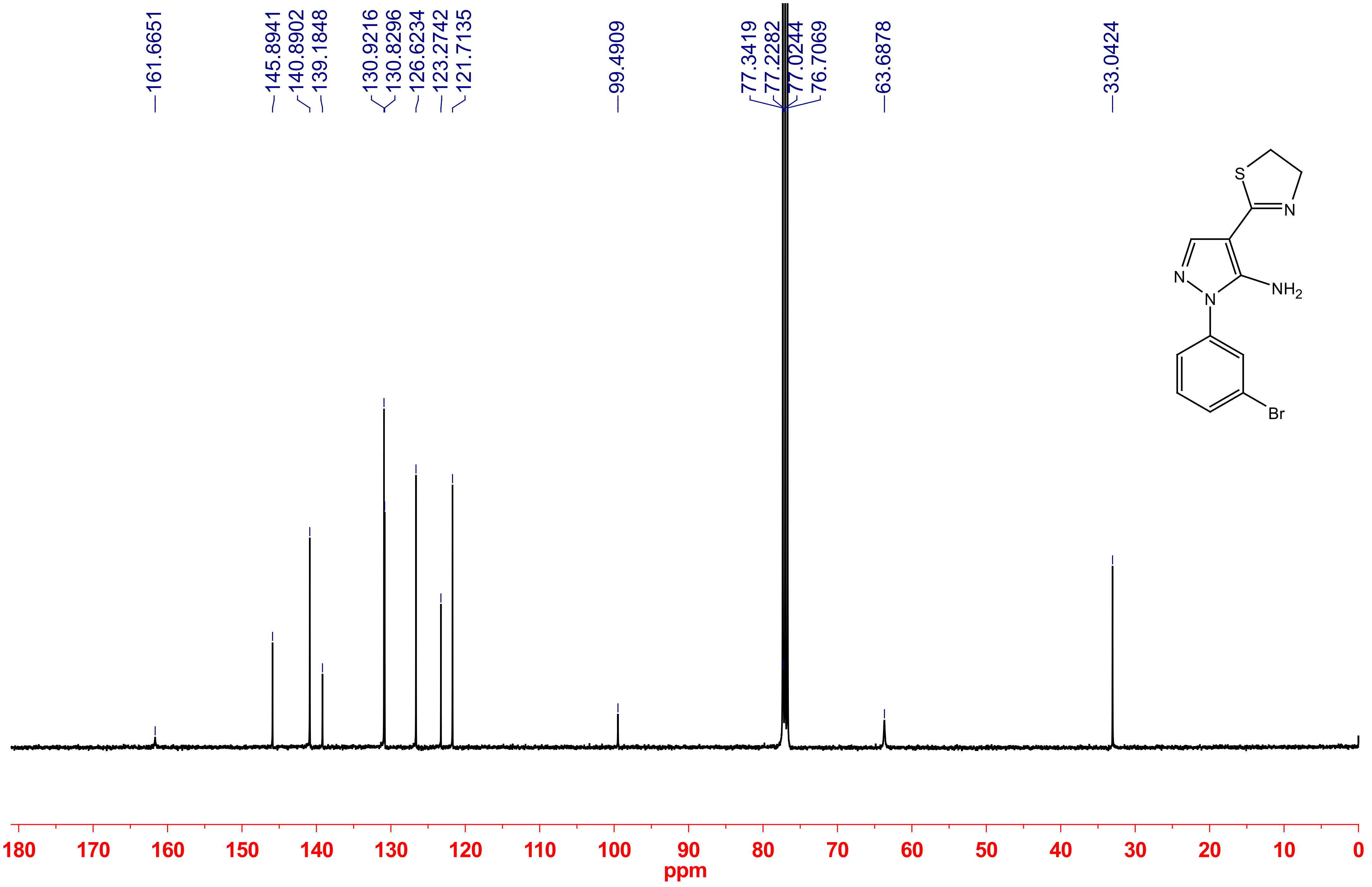

^{1}H NMR of compound **1k**

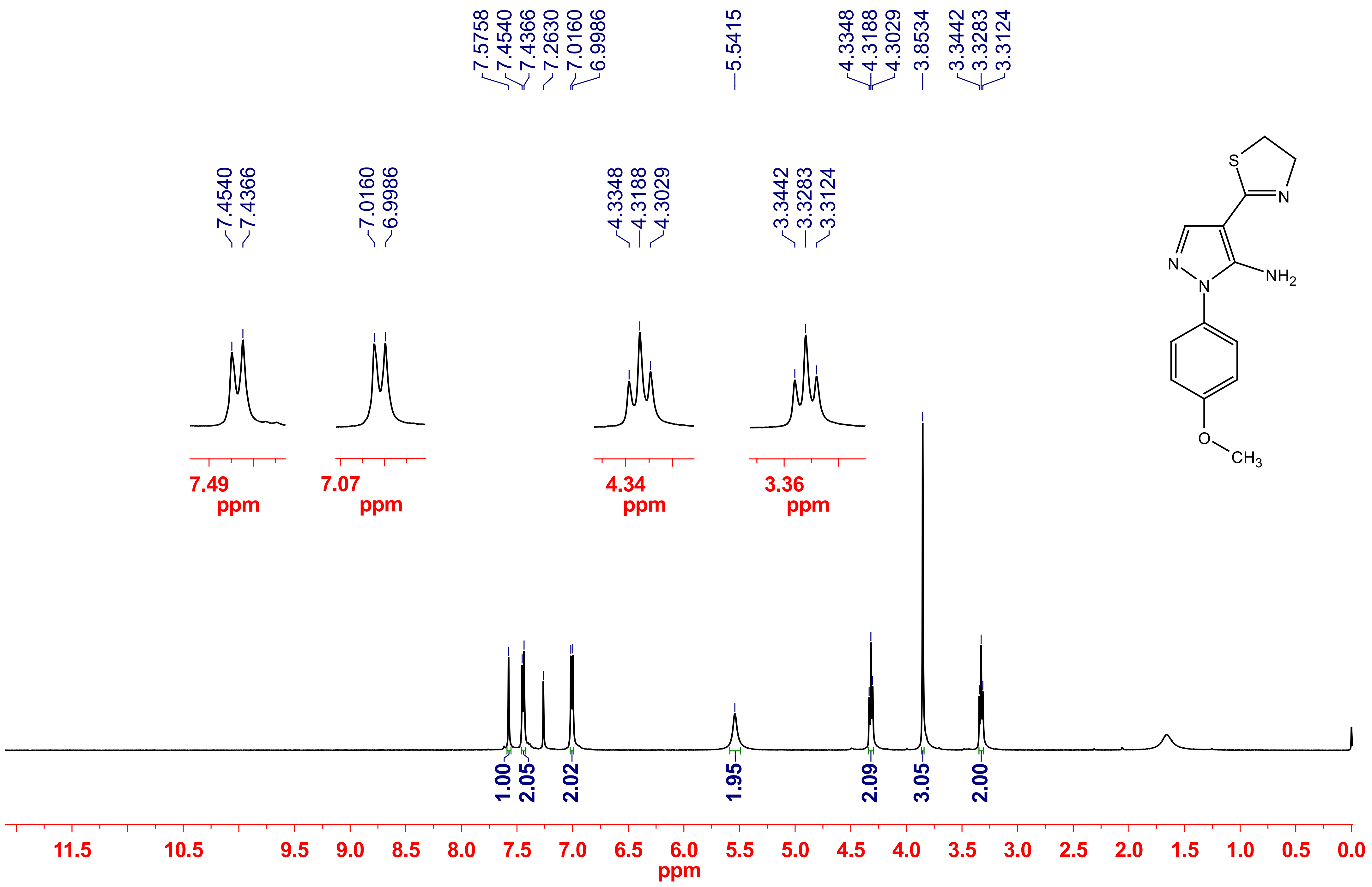

^{13}C NMR of compound **1k**

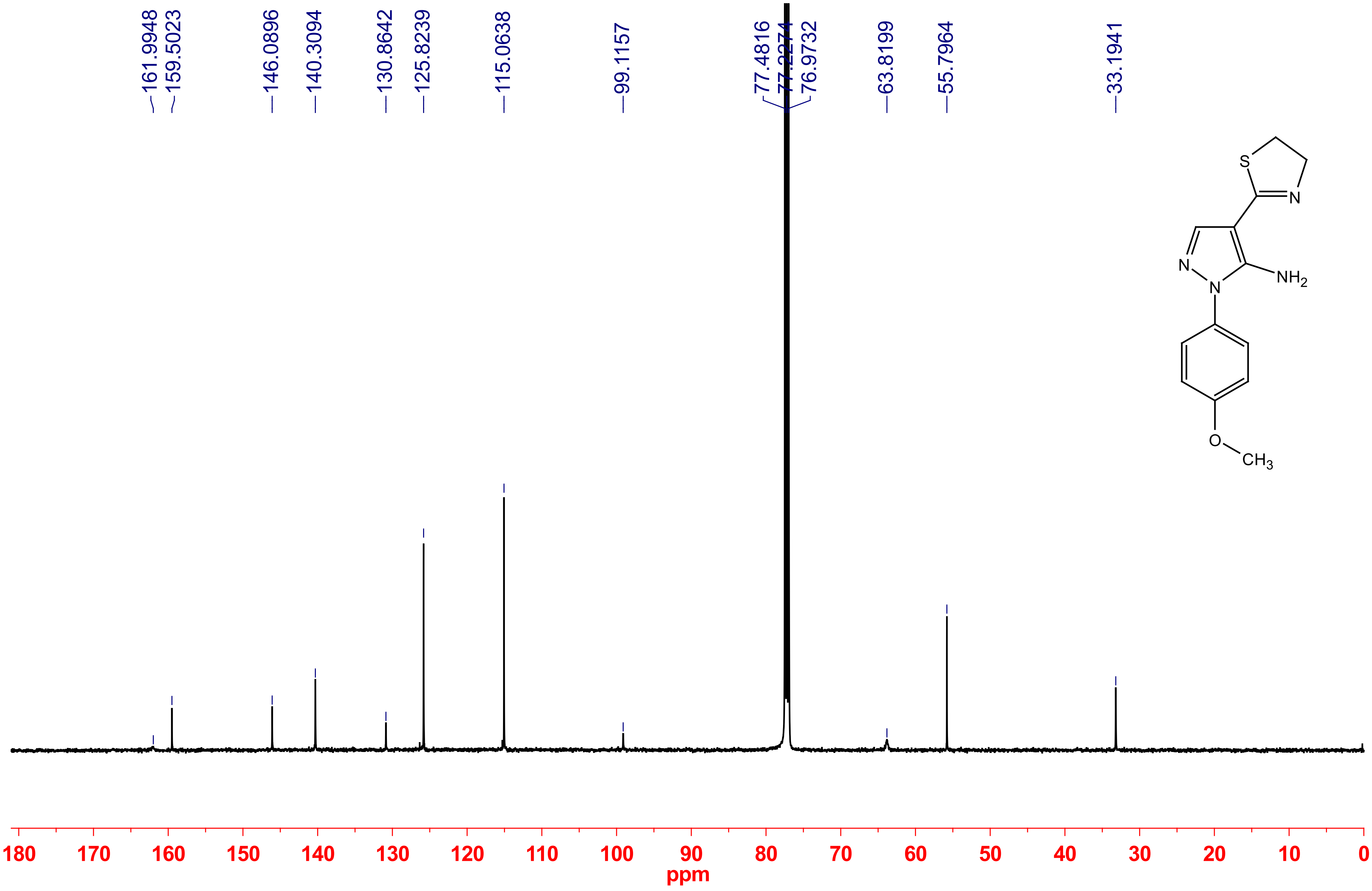

^{1}H NMR of compound **1l**

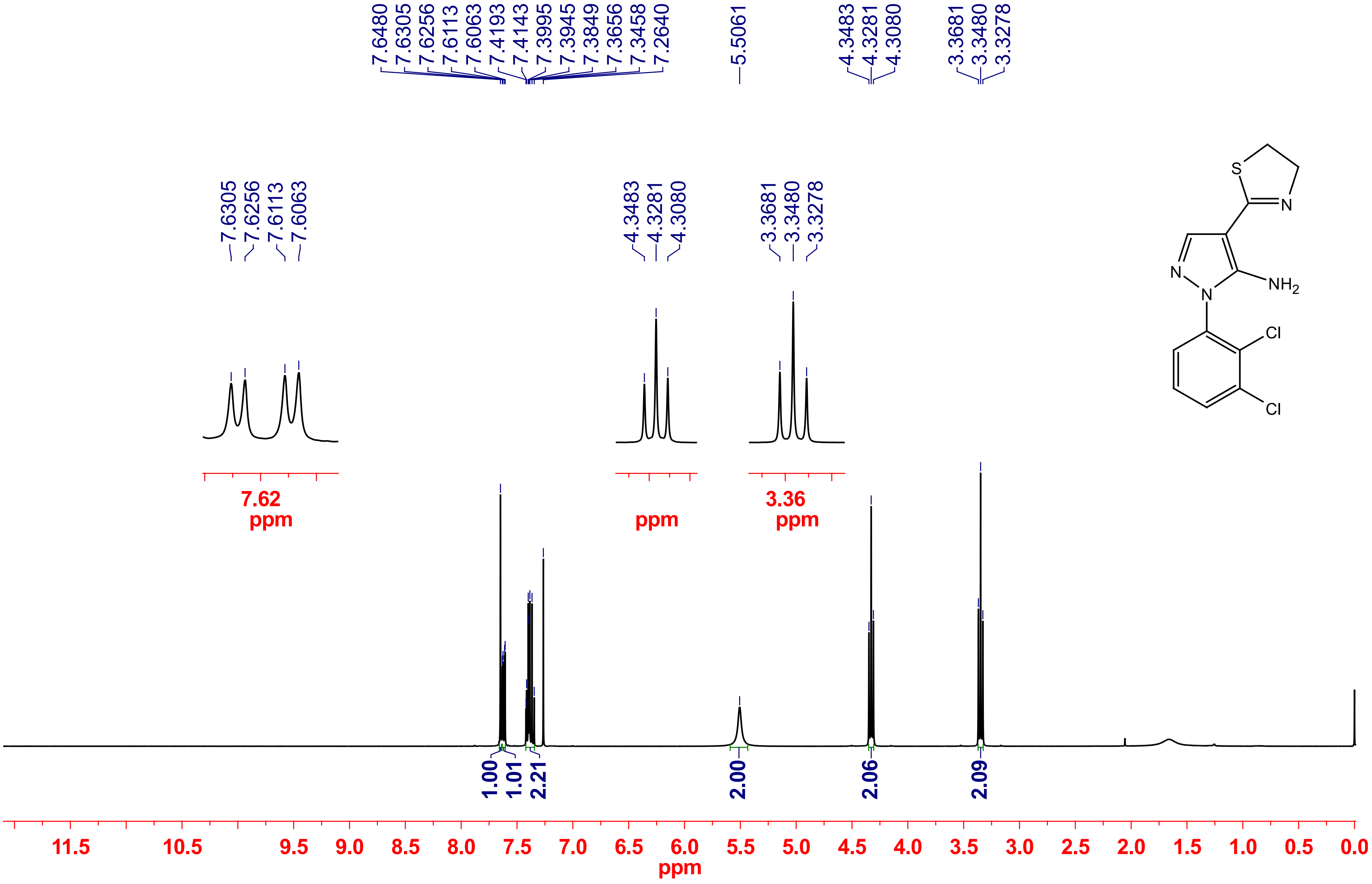

^{13}C NMR of compound **1l**

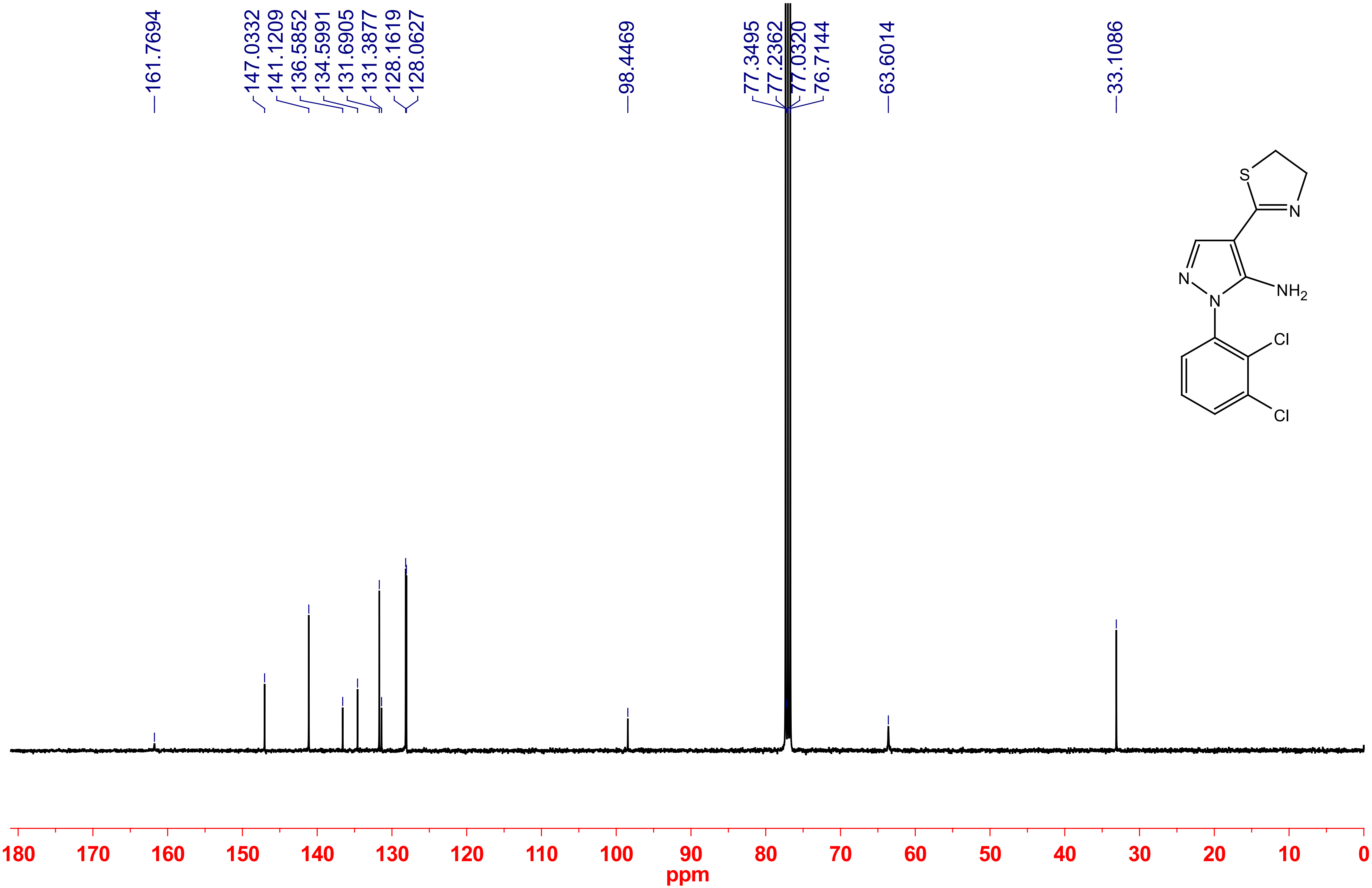

^{1}H NMR of compound **2a**

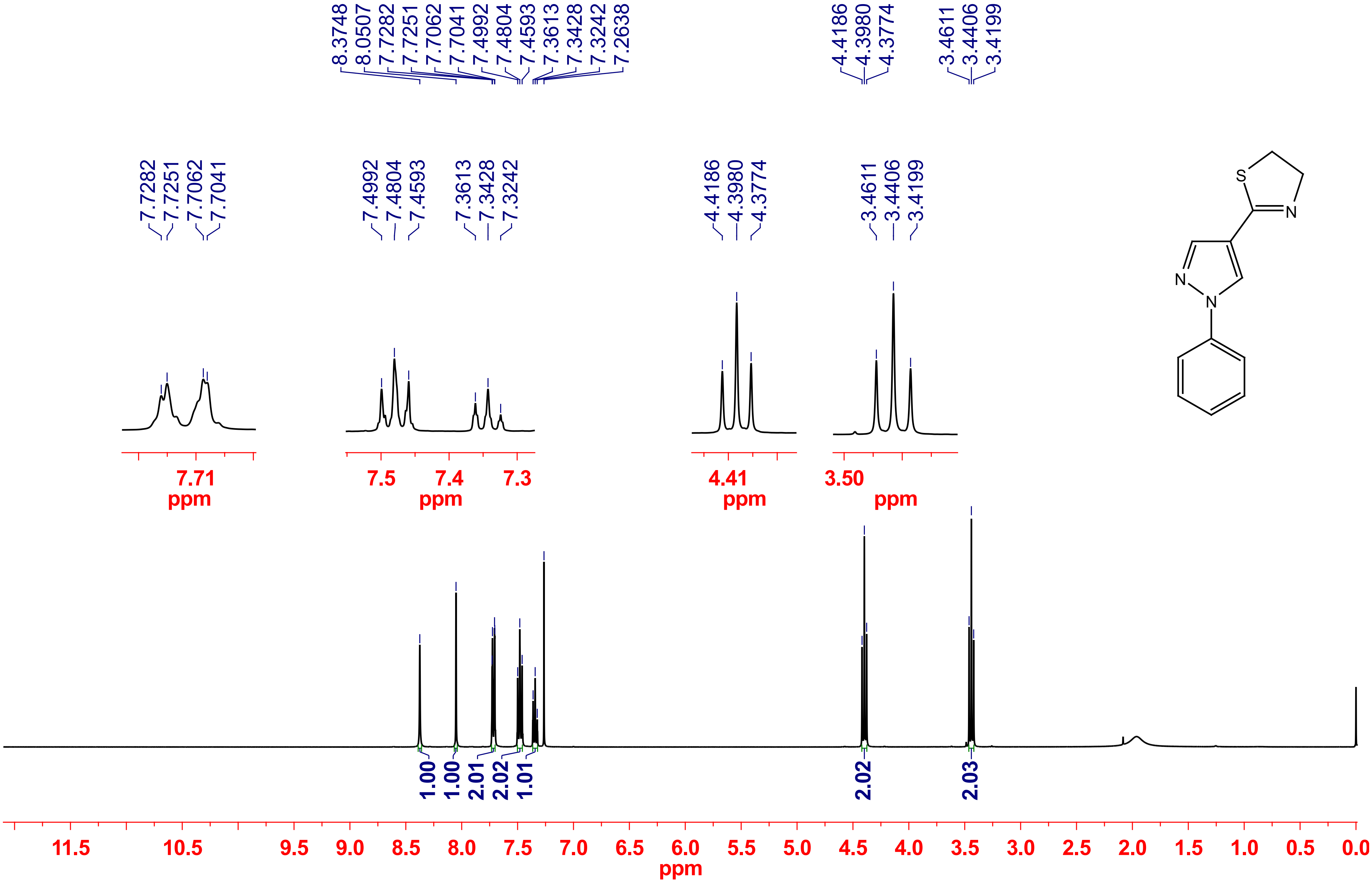

^{13}C NMR of compound **2a**

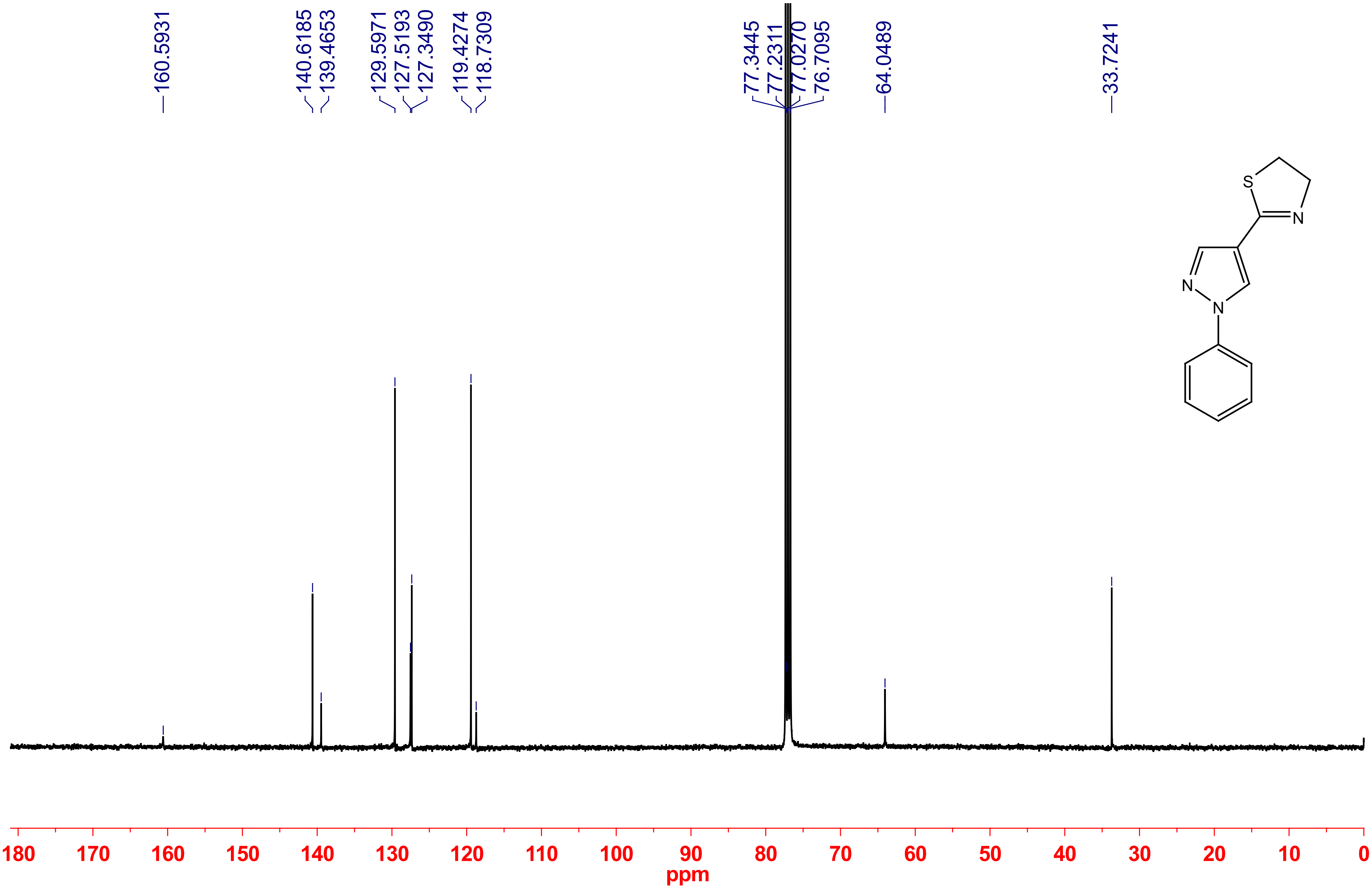

^{1}H NMR of compound **2b**

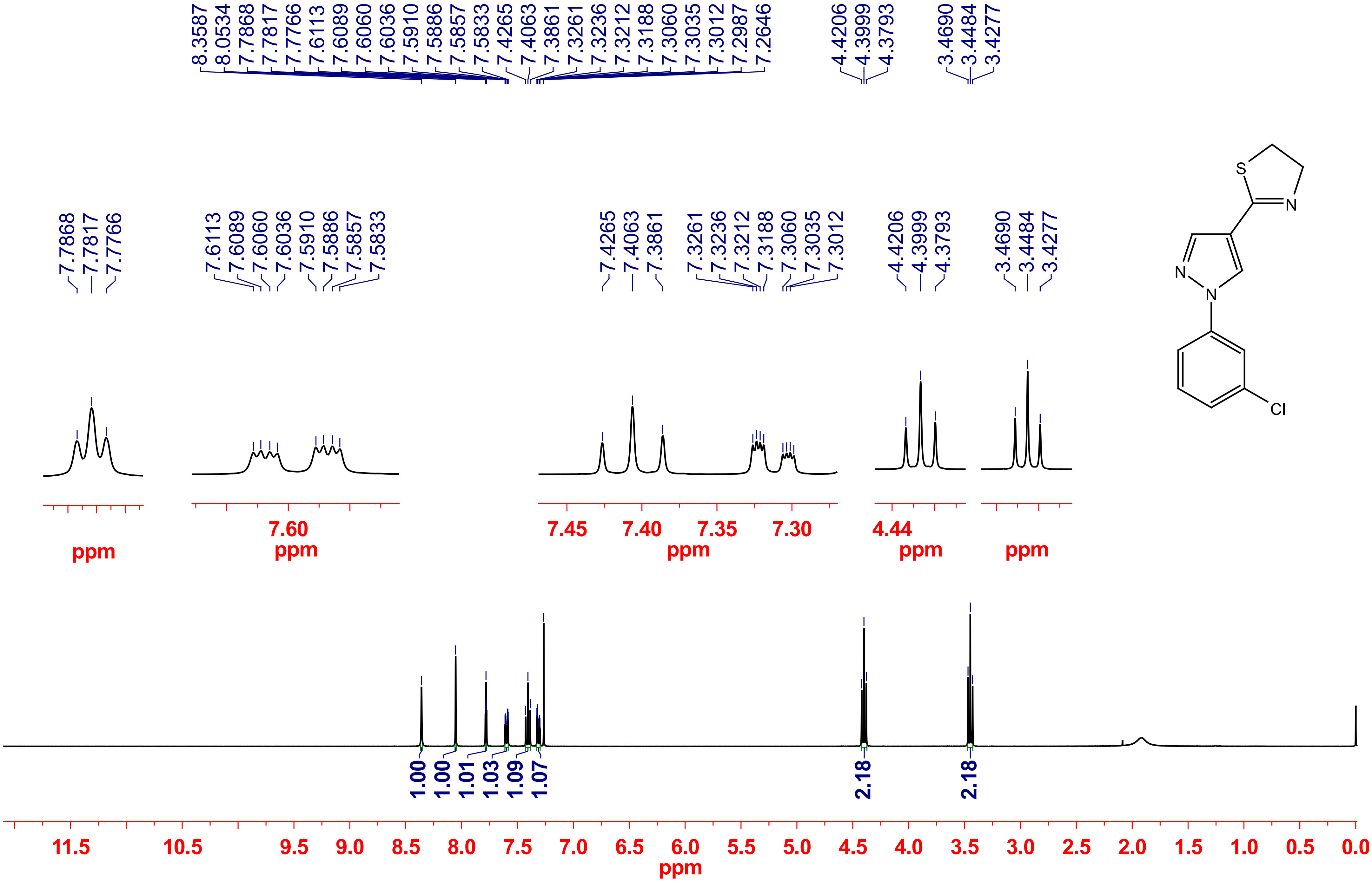

^{13}C NMR of compound **2b**

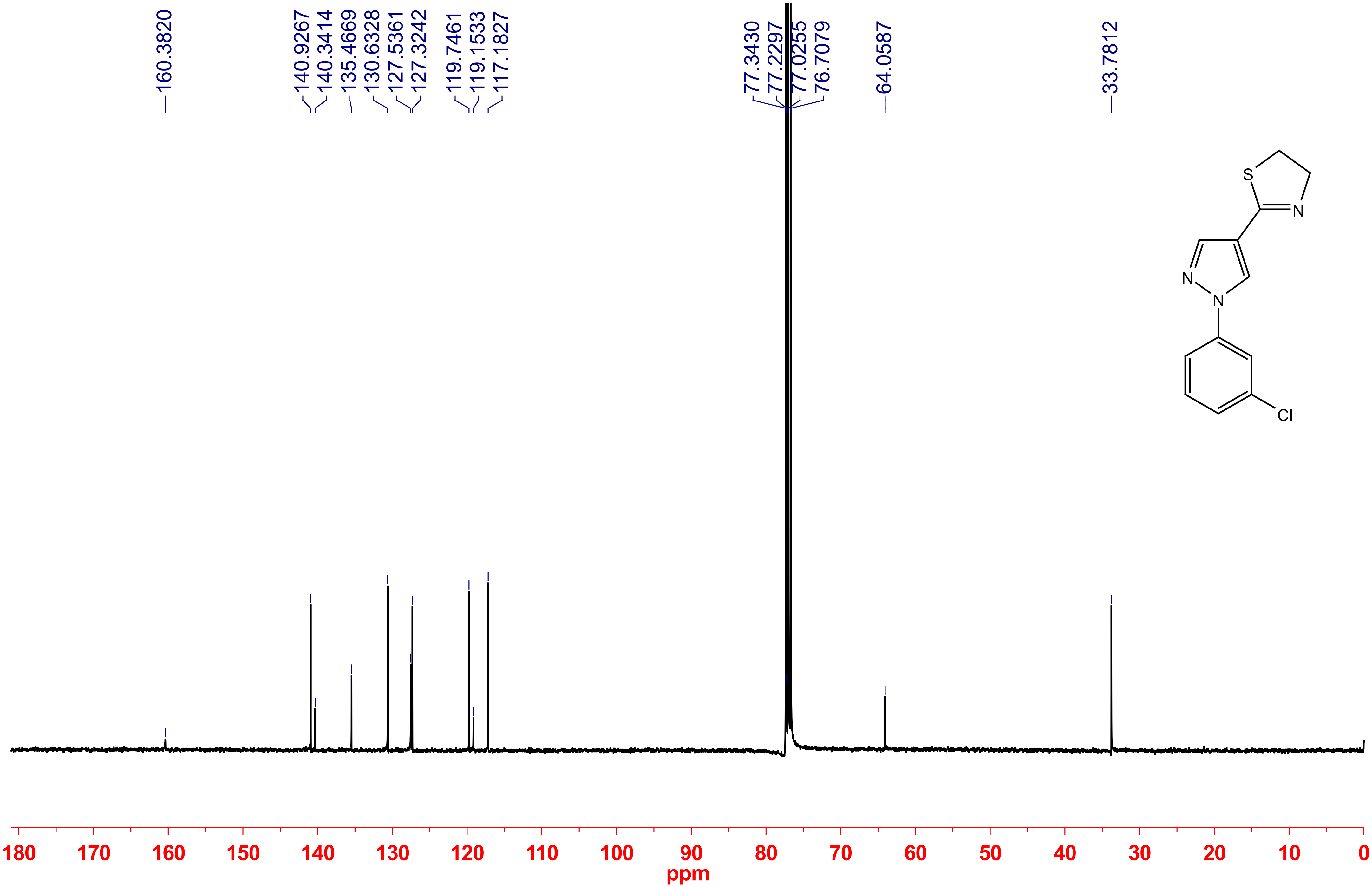

^{1}H NMR of compound **2c**

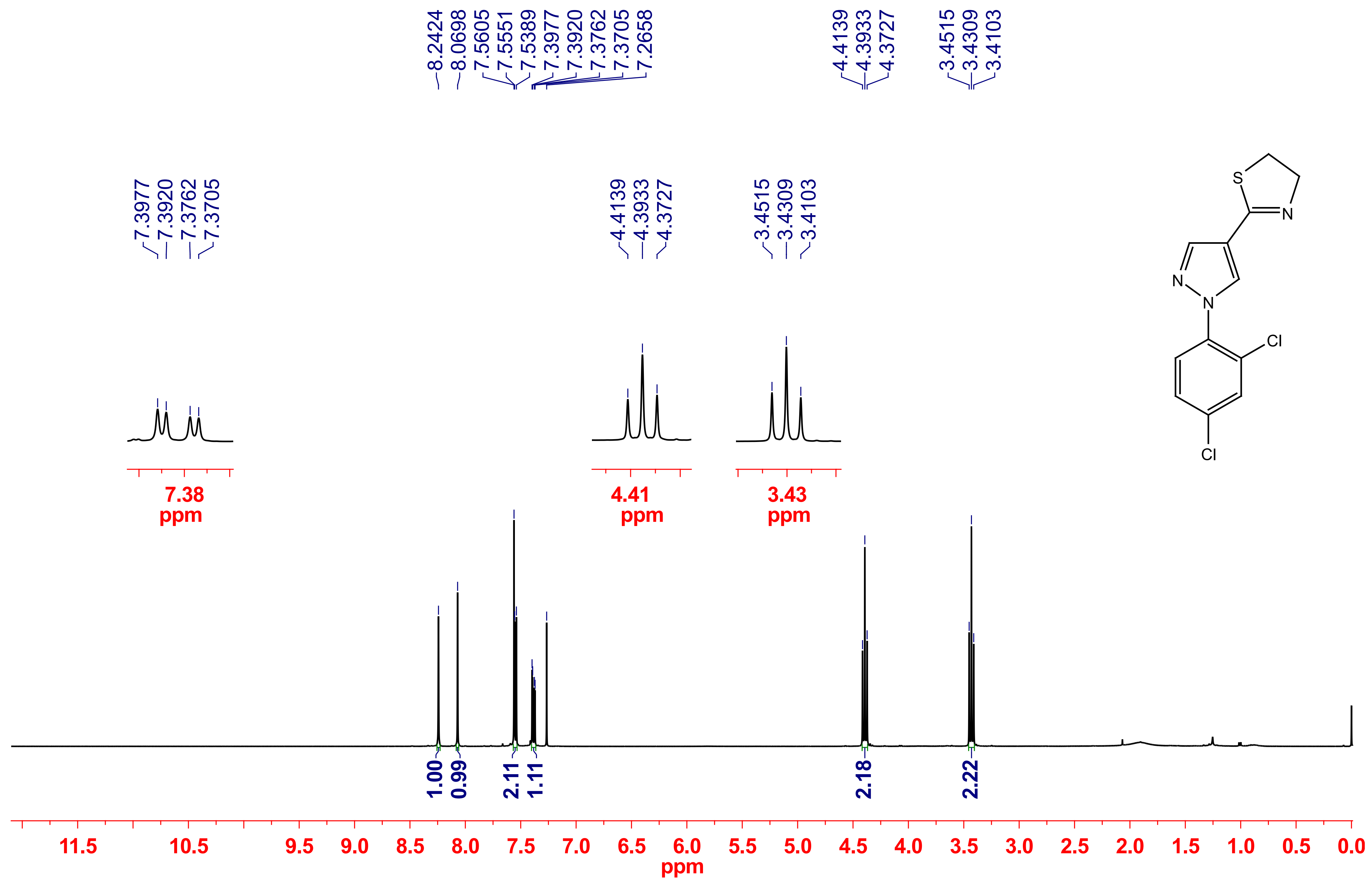

^{13}C NMR of compound **2c**

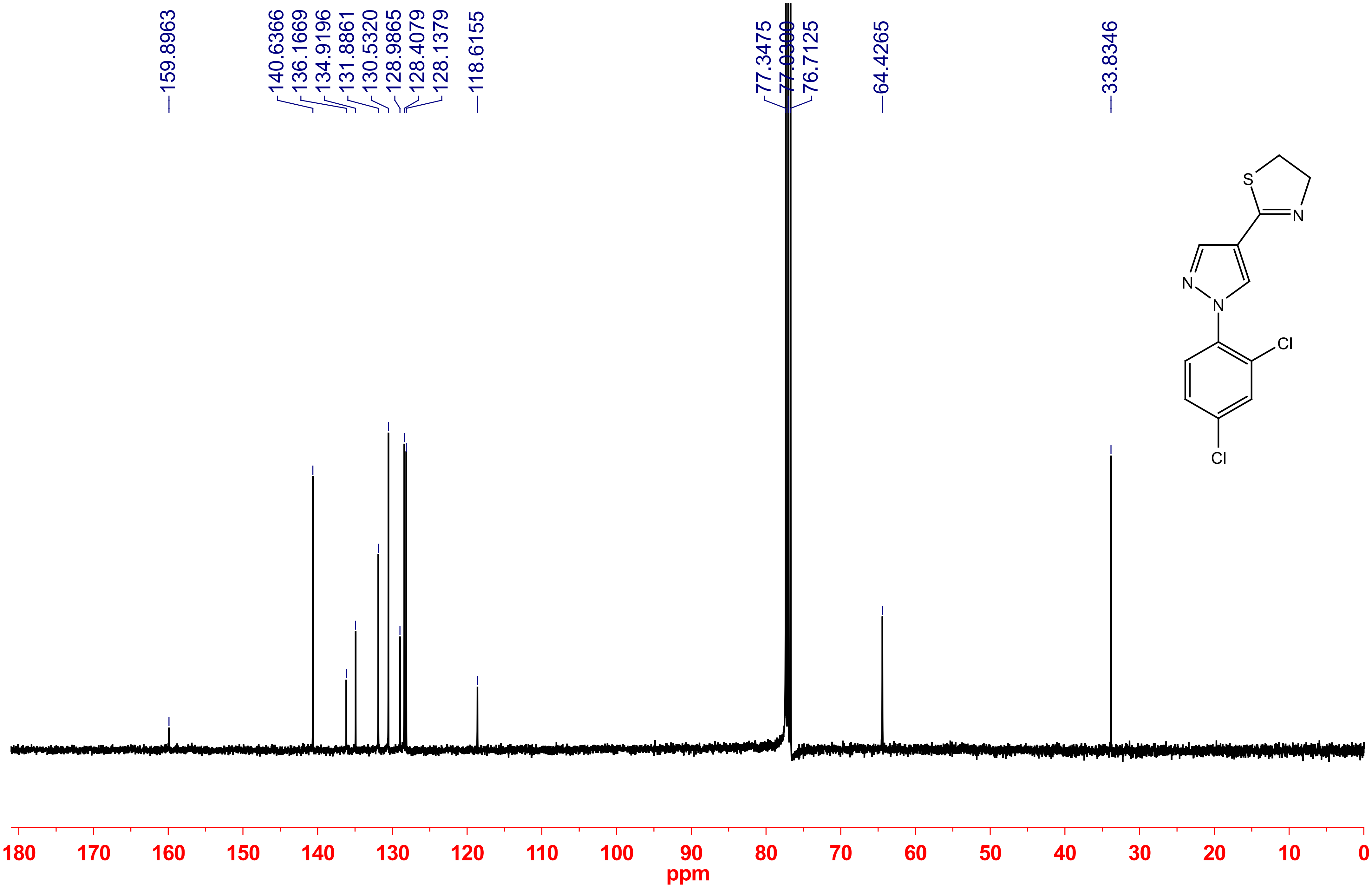

^{1}H NMR of compound **2d**

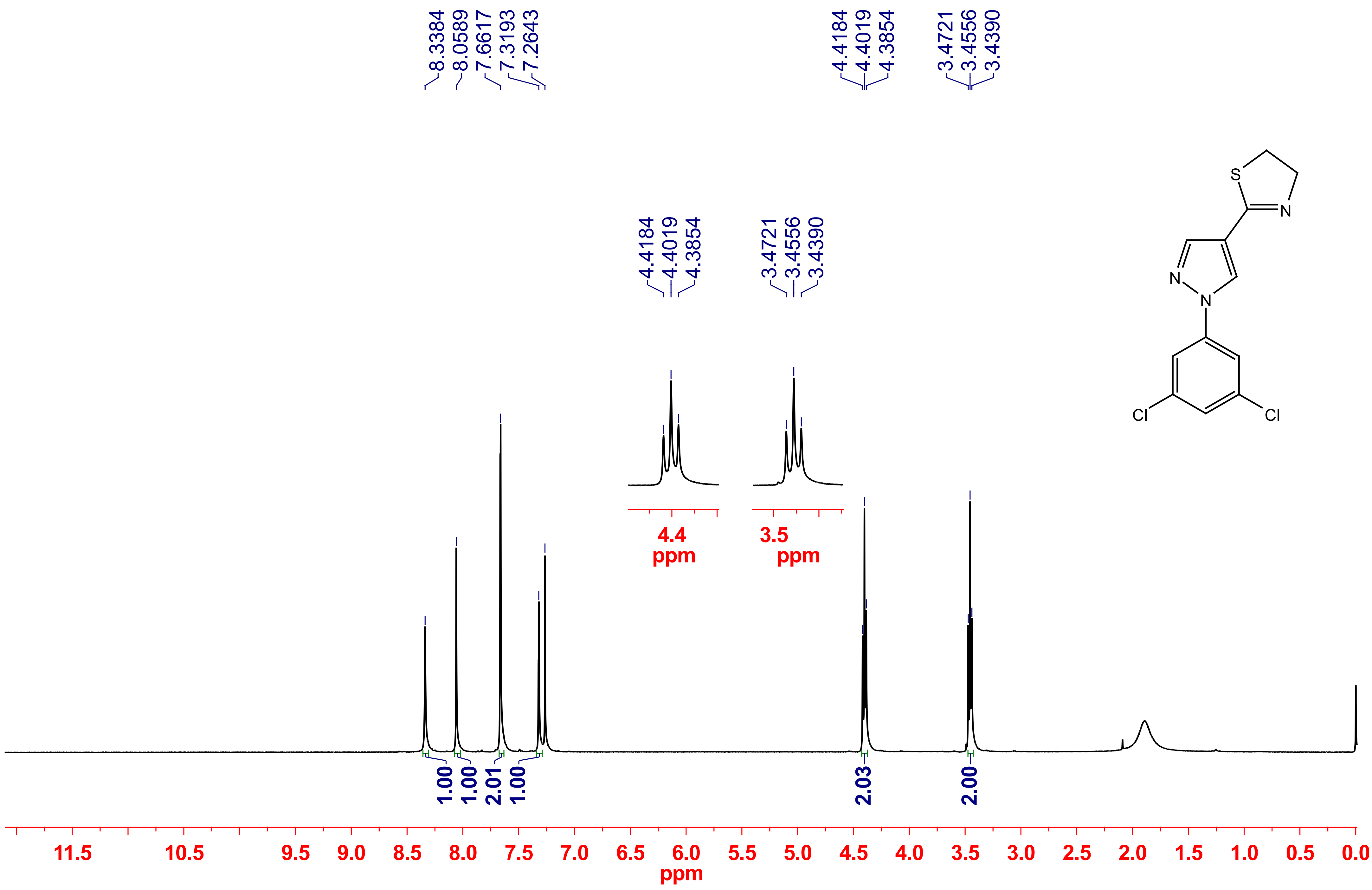

^{13}C NMR of compound **2d**

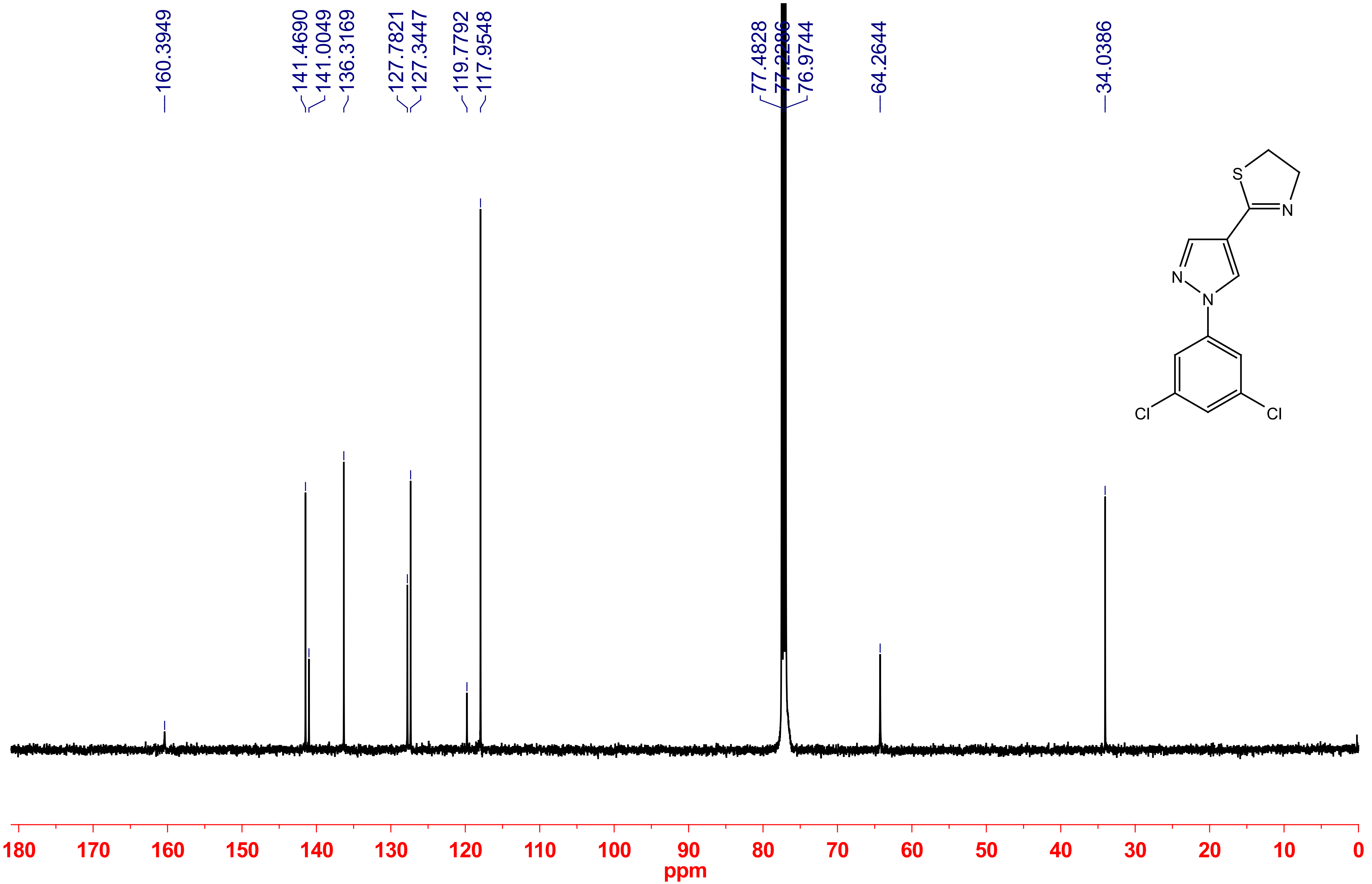

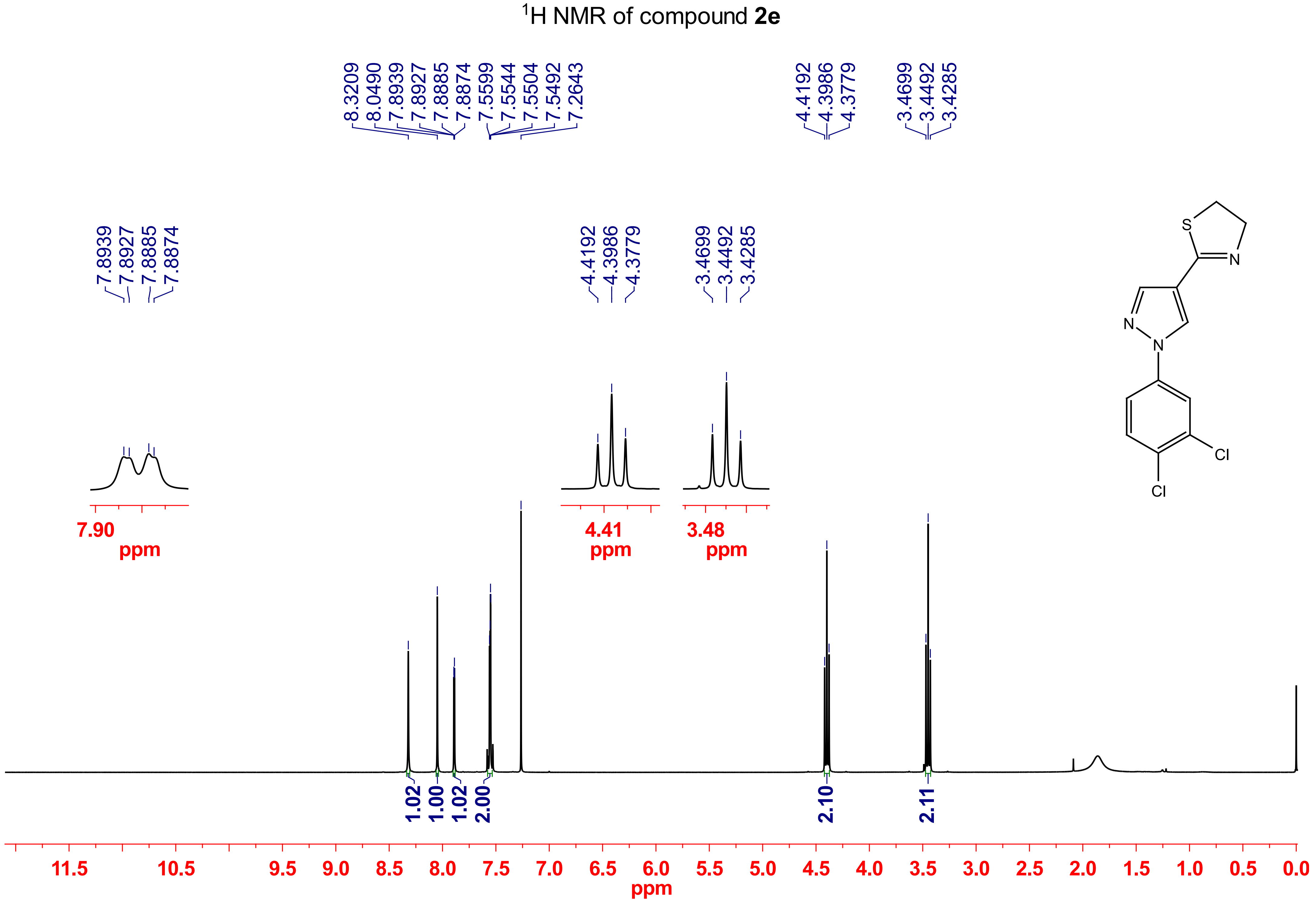

^{1}H NMR of compound **2e**
8.3209
8.0490
7.8939
7.8927
7.8885
7.8874
7.5599
7.5544
7.5504
7.5492
7.2643
4.4192
4.3986
4.3779
3.4699
3.4492
3.4285
7.90
ppm
4.41
ppm
3.48
ppm
S
N
N
N
Cl
Cl
1.02
1.00
1.02
2.00
2.10
2.11
11.5
10.5
9.5
9.0
8.5
8.0
7.5
7.0
6.5
6.0
5.5
5.0
4.5
4.0
3.5
3.0
2.5
2.0
1.5
1.0
0.5
0.0
ppm

^{13}C NMR of compound **2e**

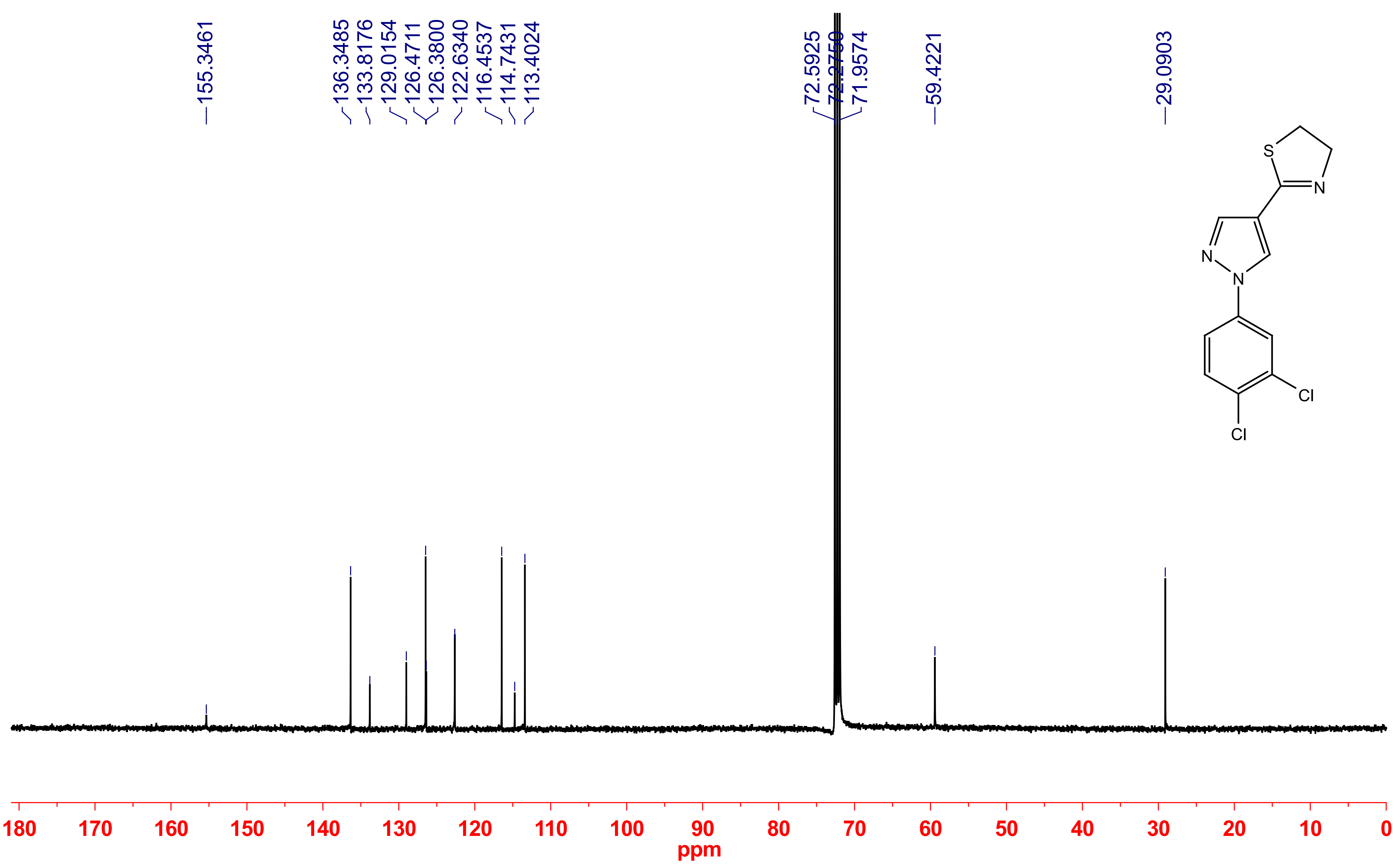

^{1}H NMR of compound **2f**

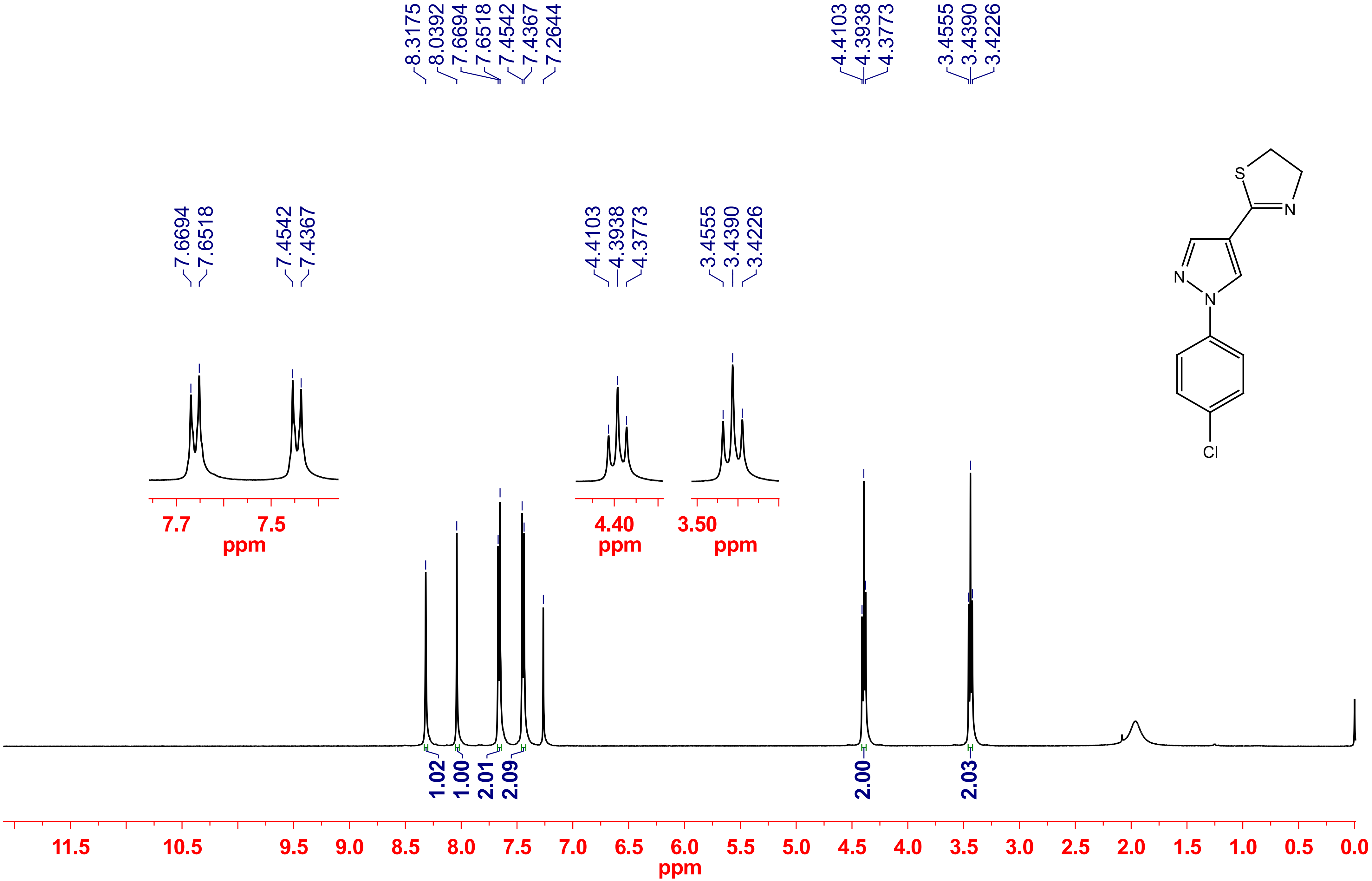

^{13}C NMR of compound **2f**

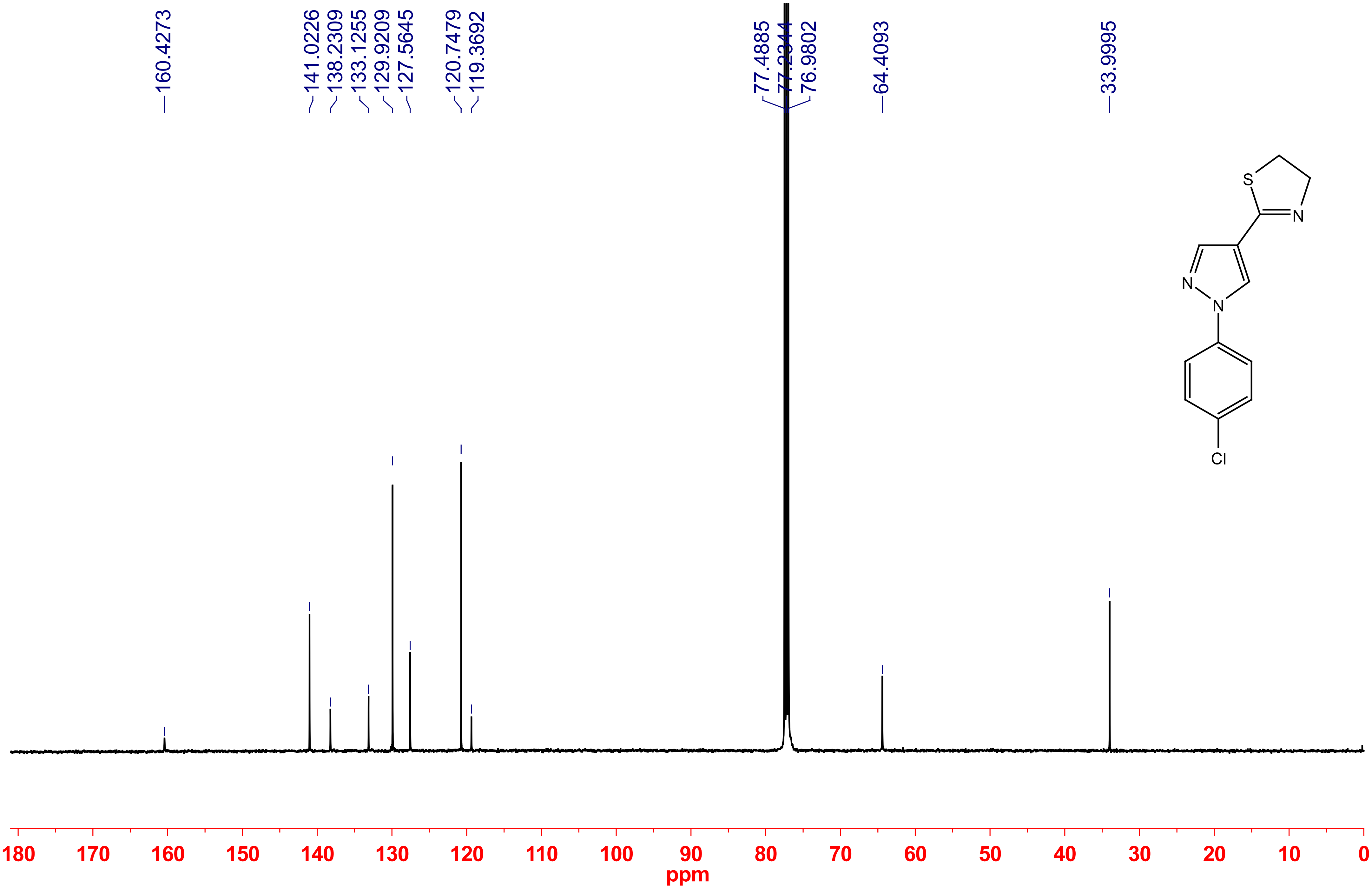

^{1}H NMR of compound **2g**

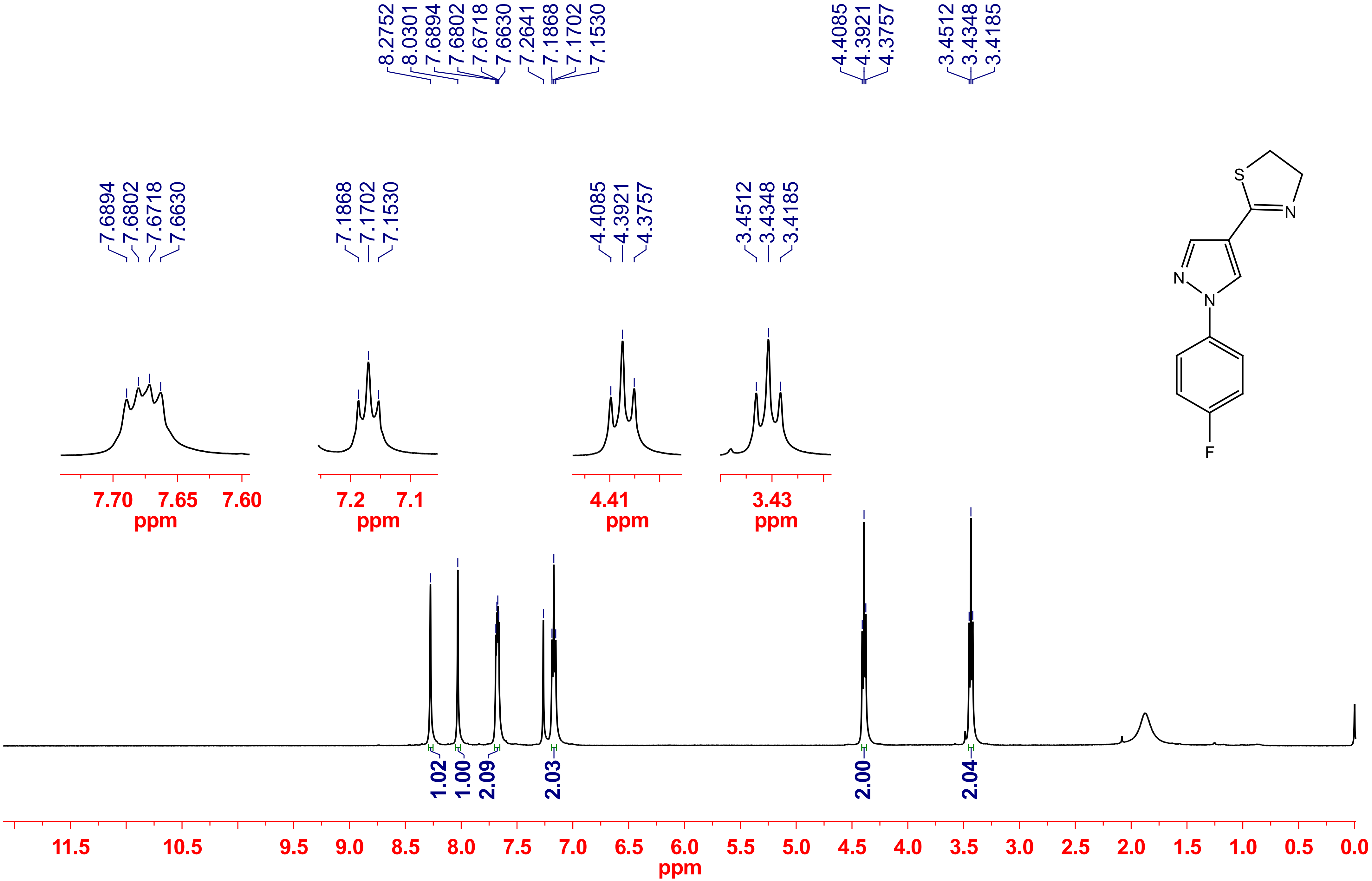

^{13}C NMR of compound **2g**

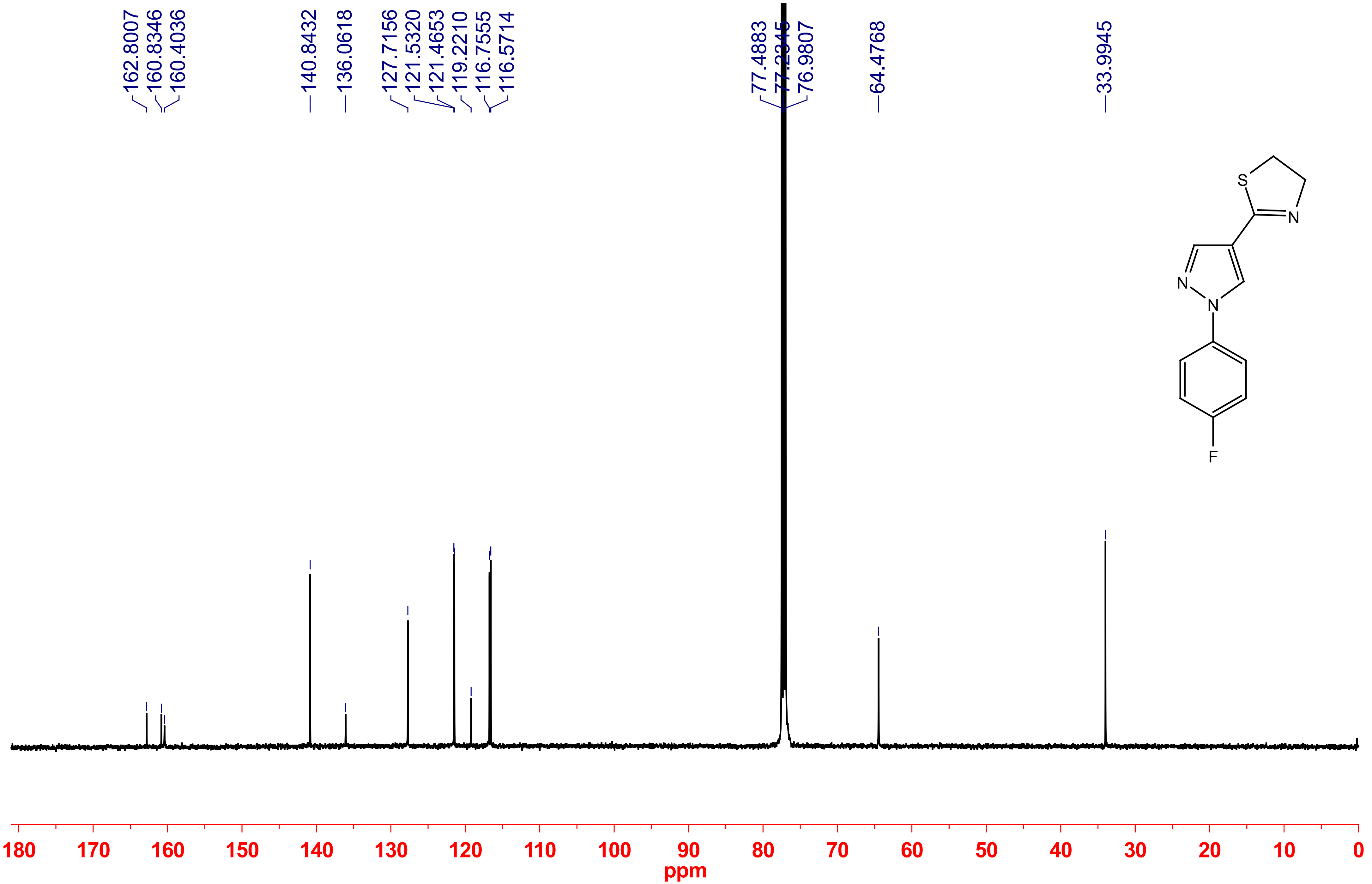

^{1}H NMR of compound **2h**

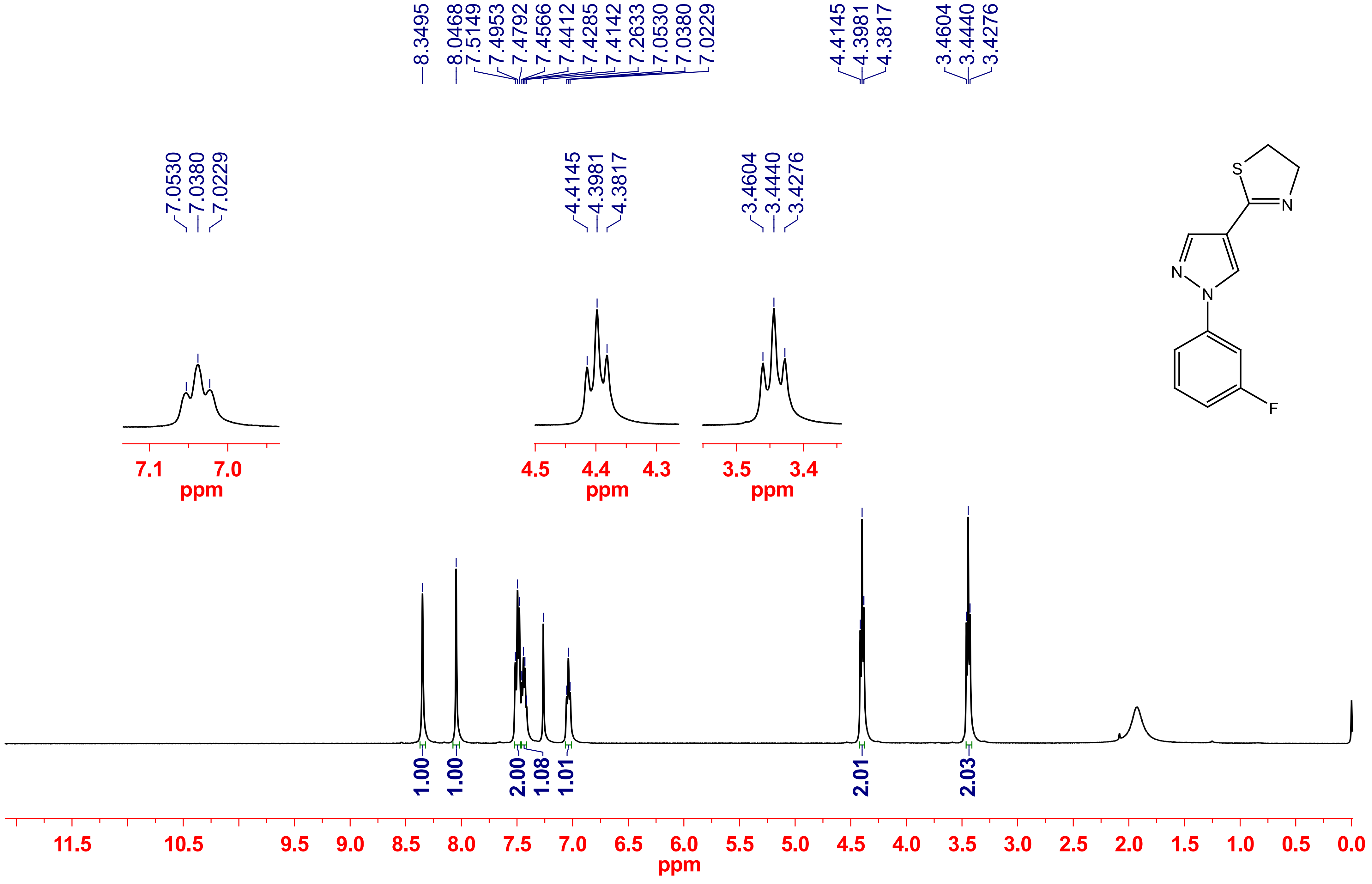

^{13}C NMR of compound **2h**

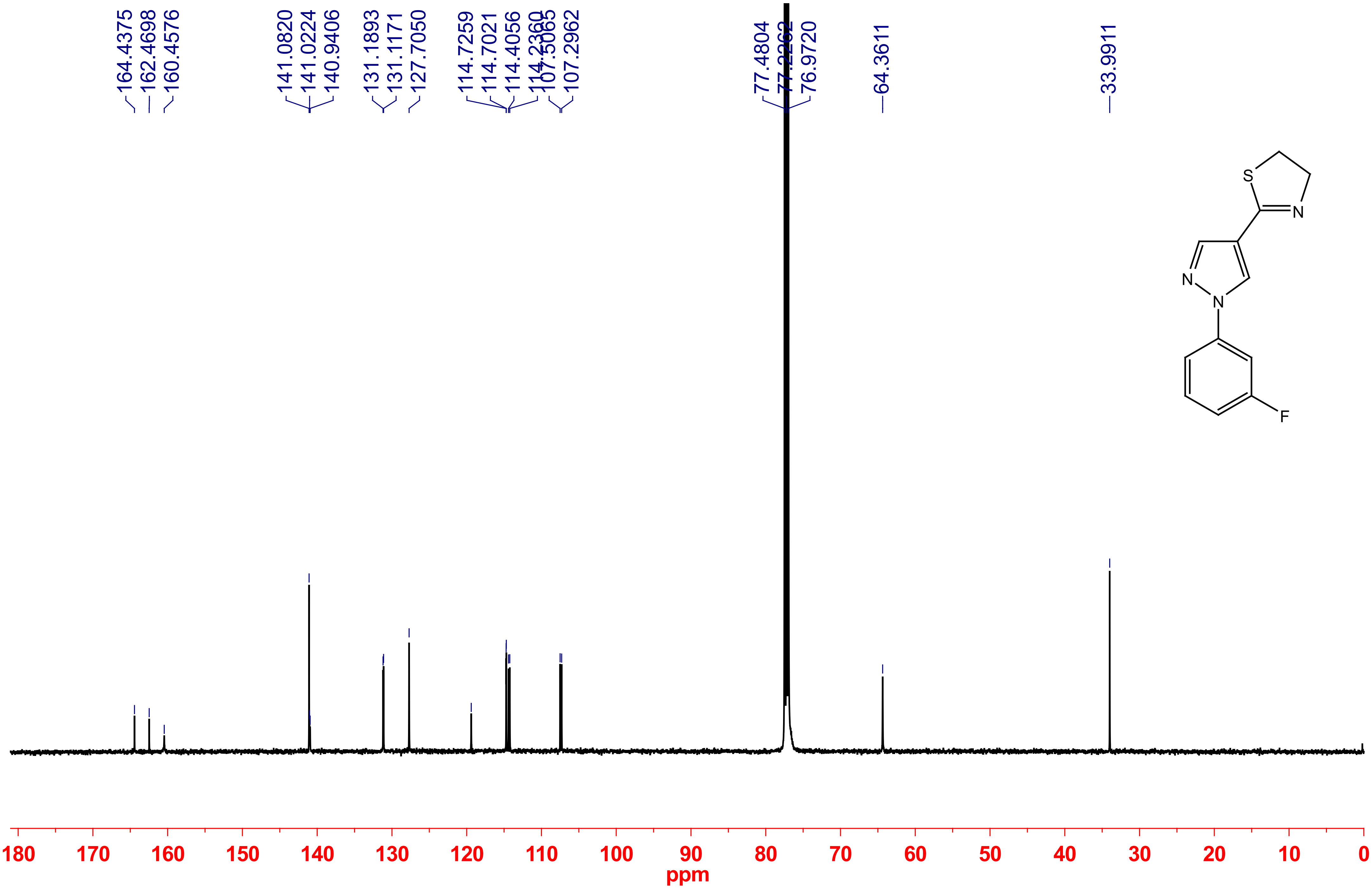

^{1}H NMR of compound **2i**

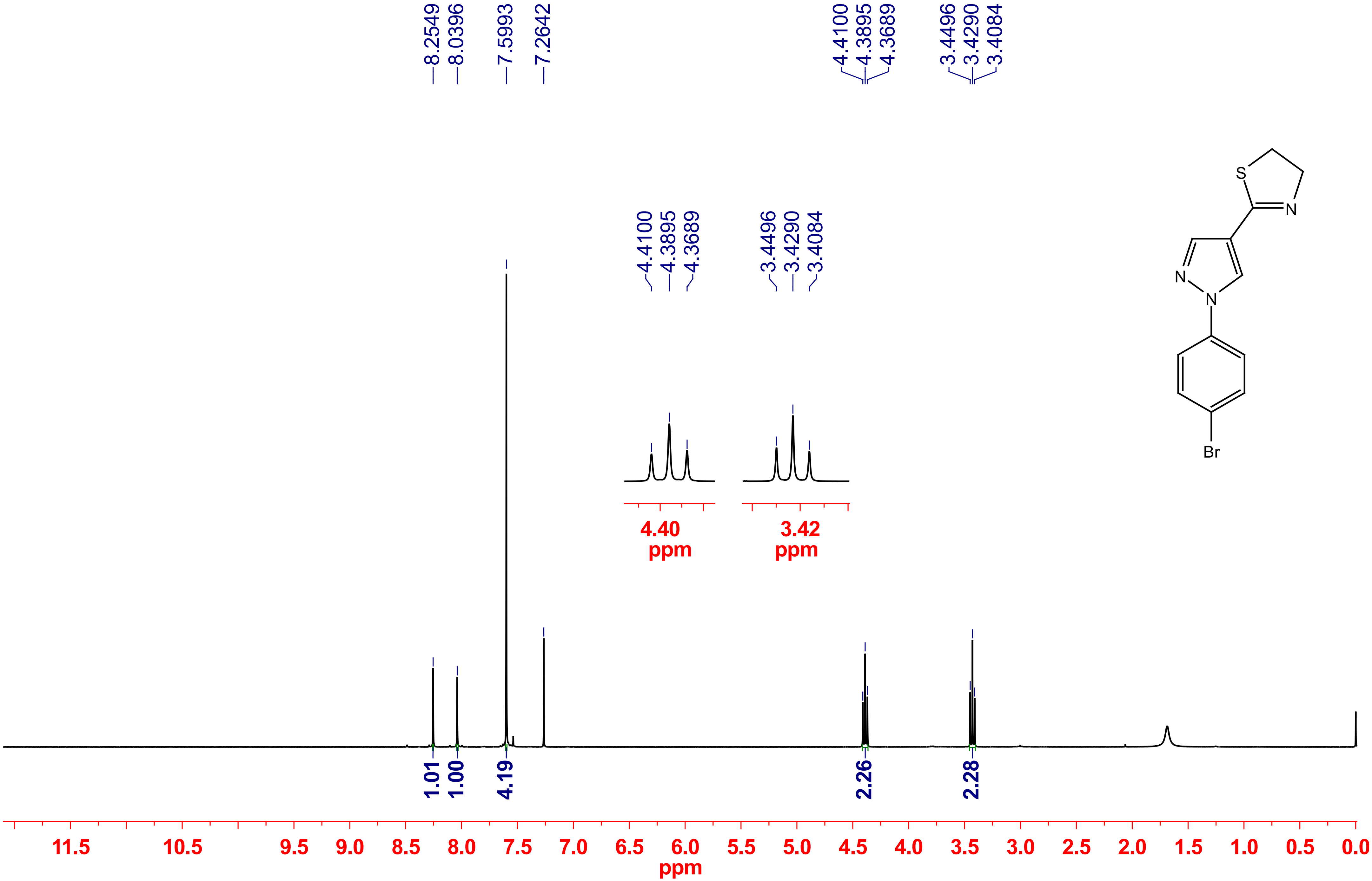

^{13}C NMR of compound **2i**

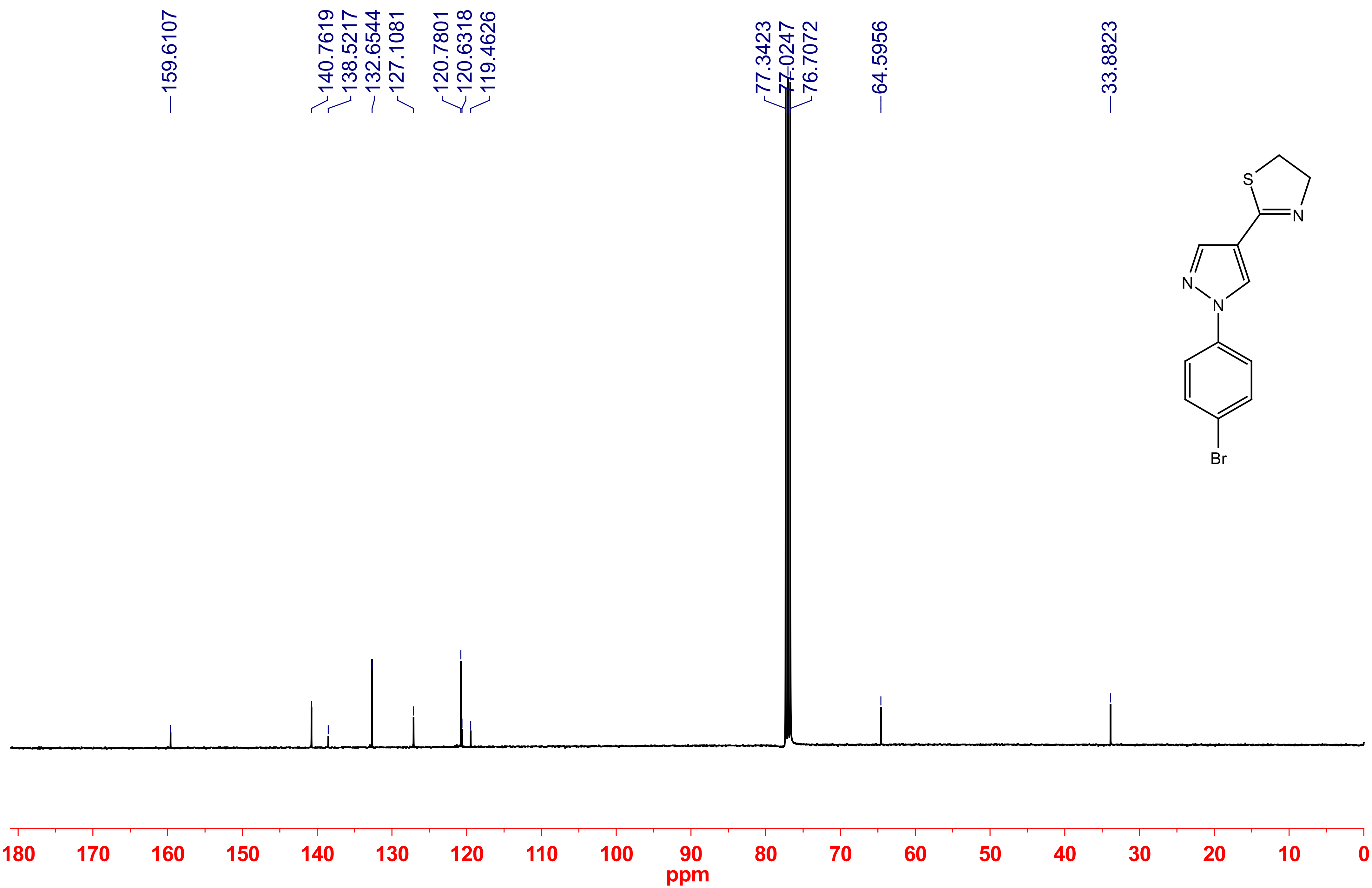

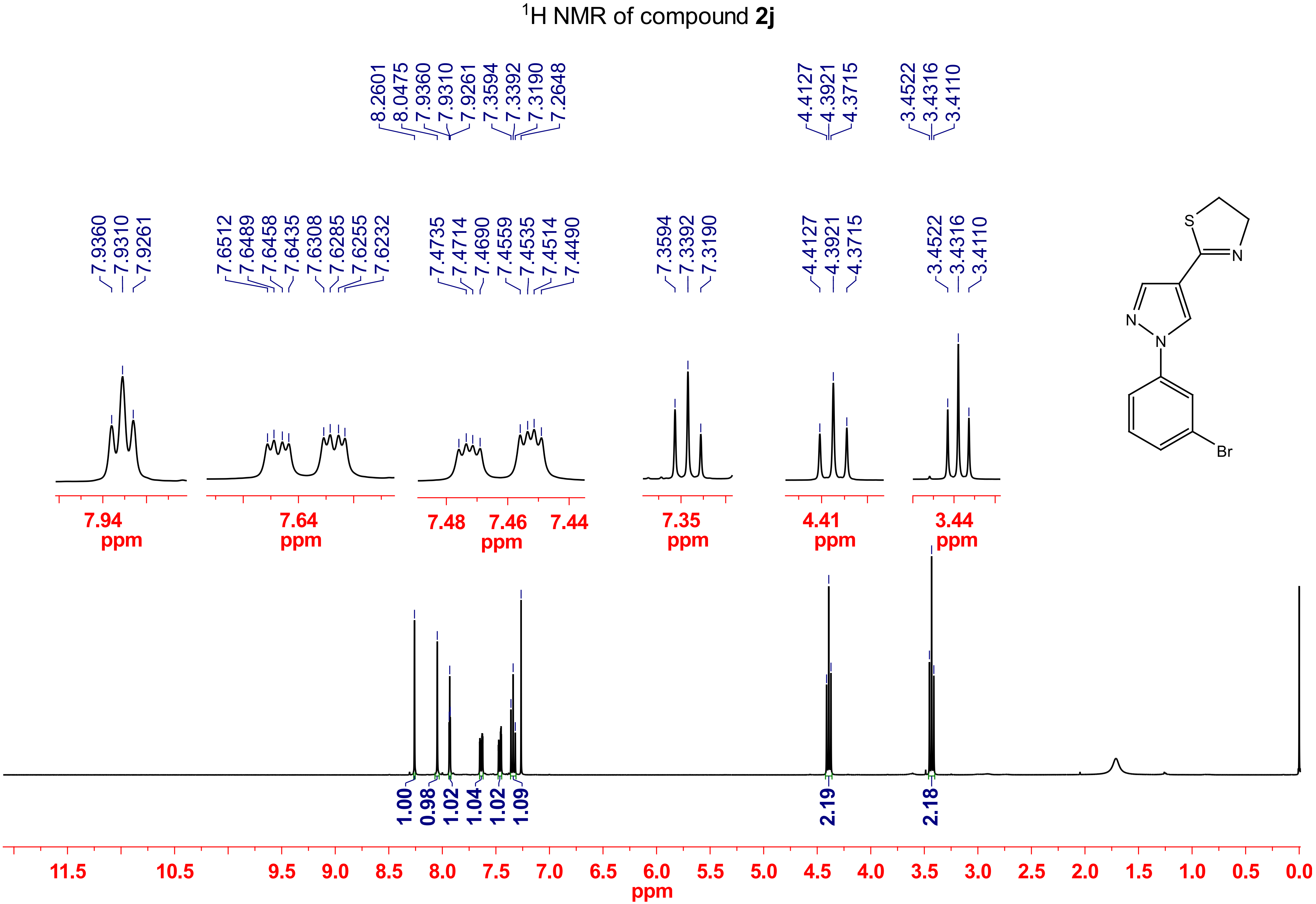

^{1}H NMR of compound **2j**
8.2601
8.0475
7.9360
7.9310
7.9261
7.6512
7.6489
7.6458
7.6435
7.6308
7.6285
7.6255
7.6232
7.4735
7.4714
7.4690
7.4559
7.4535
7.4514
7.4490
7.3594
7.3392
7.3190
7.2648
4.4127
4.3921
4.3715
3.4522
3.4316
3.4110
7.94 ppm
7.64 ppm
7.48 7.46 7.44 ppm
7.35 ppm
4.41 ppm
3.44 ppm
S
N
N
N
Br
1.00
0.98
1.02
1.04
1.02
1.09
2.19
2.18
11.5 10.5 9.5 9.0 8.5 8.0 7.5 7.0 6.5 6.0 5.5 5.0 4.5 4.0 3.5 3.0 2.5 2.0 1.5 1.0 0.5 0.0
ppm

^{13}C NMR of compound **2j**

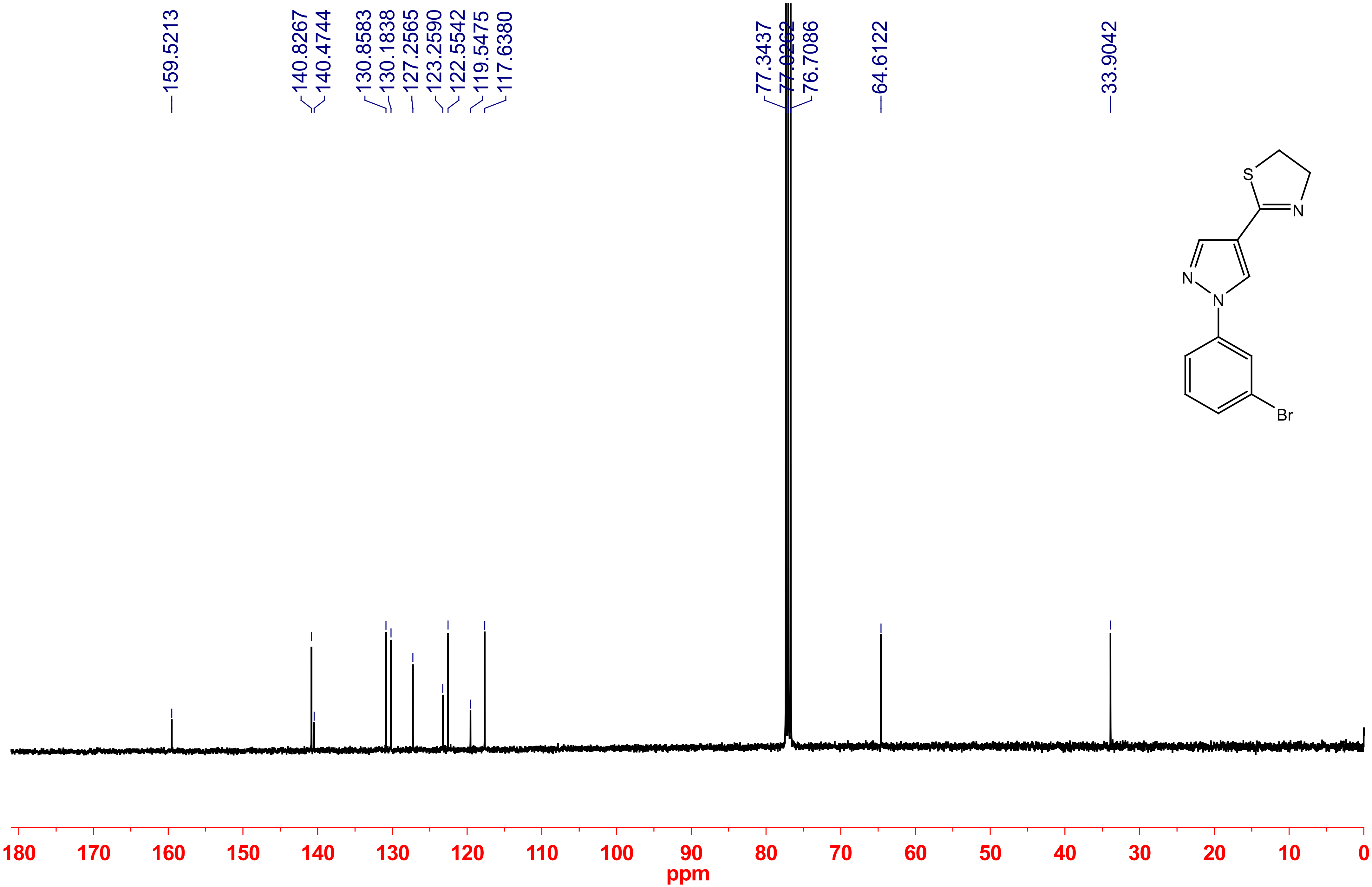

^{1}H NMR of compound **2k**

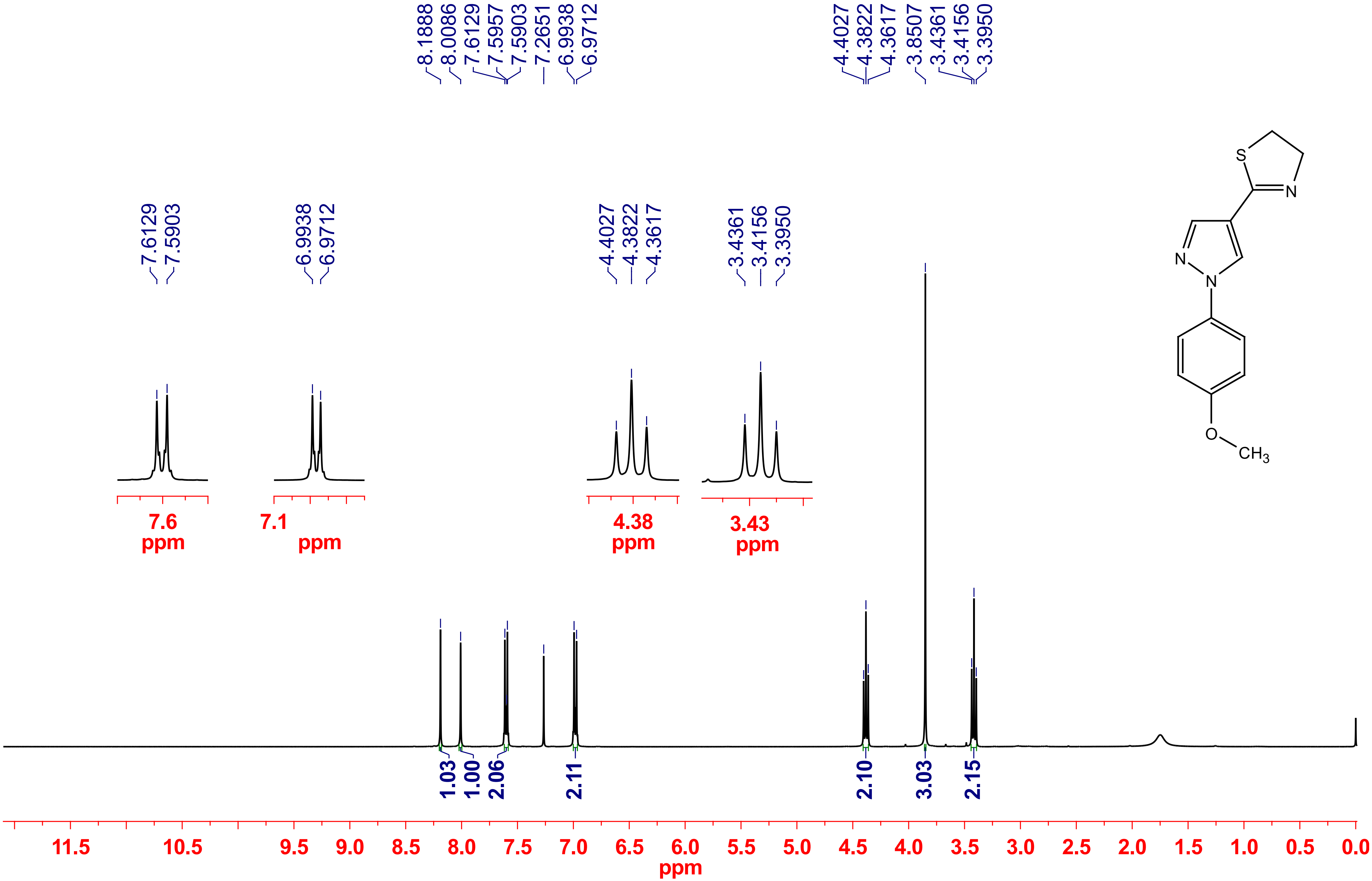

^{13}C NMR of compound **2k**

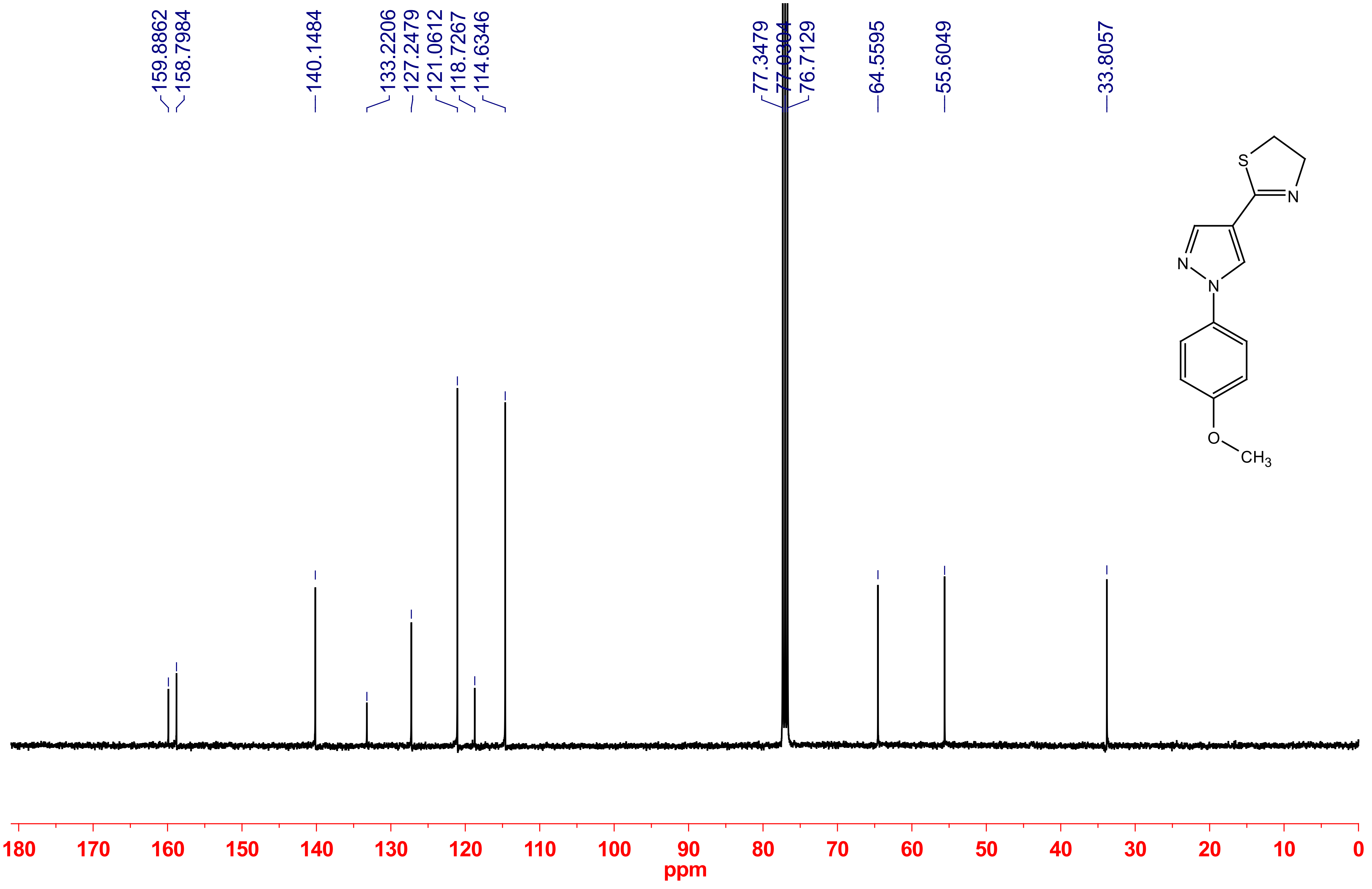

^{1}H NMR of compound **2l**

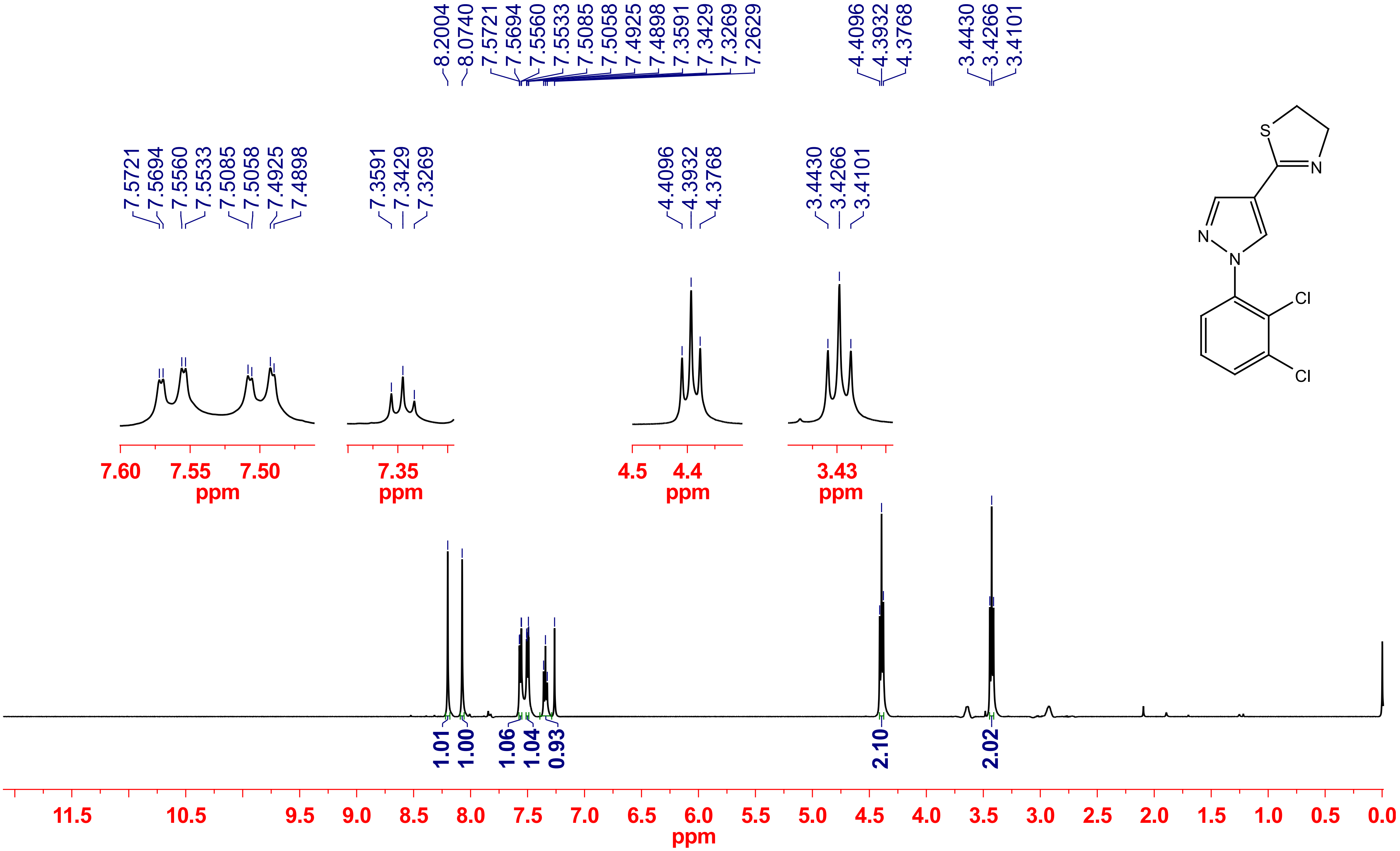

^{13}C NMR of compound **2l**

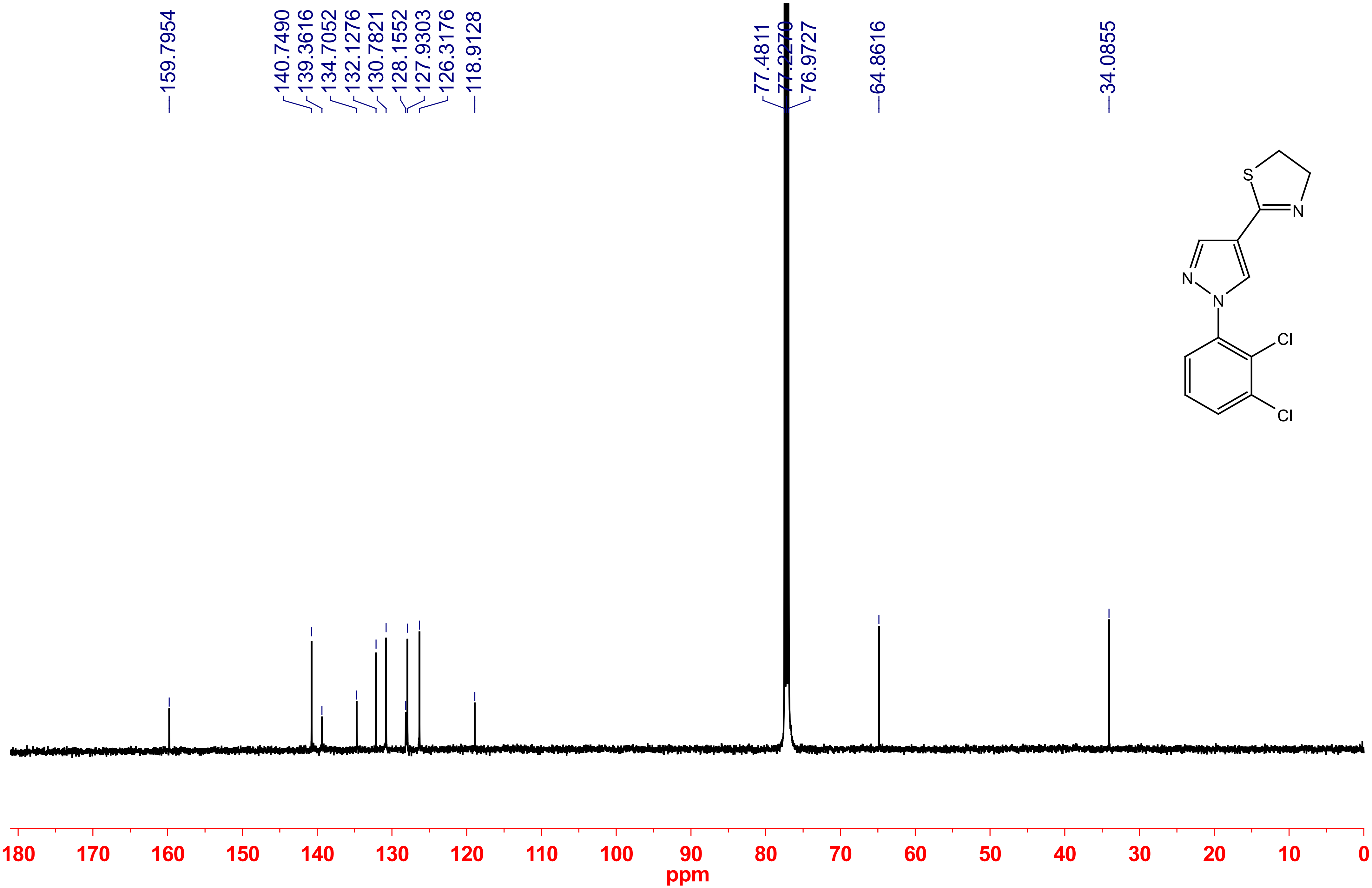

Supplement: Supplementary file 1 [file pharmaceutics-14-00995-s001.zip › Figure S1.pdf]
