# Supplementary material for: Bioactivity of Novel Pyrazole-Thiazolines Scaffolds against Trypanosoma cruzi: Computational Approaches and 3D Spheroid Model on Drug Discovery for Chagas Disease
Source: Pharmaceutics. 2022 May 5;14(5):995. doi: 10.3390/pharmaceutics14050995 (PMC9146228; doi:10.3390/pharmaceutics14050995)
Supplement: Supplementary file 1 [file pharmaceutics-14-00995-s001.zip › Table S1.pdf]

Table S1. Predicted ADMET profile

Property (unit)	Compounds<br>Predicted result			Inference/reference range
	**2c** (BSB3)	**2e** (BSB5)	**2i** (BSB13)	
	**Absorption**			
Papp (Caco-2 permeability) (cm/s)	Optimal -4.343	Optimal -4.347	Optimal -4.527	Optimal: higher than −5.15 Log unit
HIA (Human Intestinal Absorpion) (% Probability)	+ (0.791)	+ (0.791)	+ (0.717)	≥30%: HIA+; <30%: HIA-
	**Distribution**			
PPB (Plasma protein binding) (%)	83.857	84.024	80.882	90%: Significant with drugs that are highly protein-bound and have a low therapeutic index
VD (Volume Distribution) (L/kg)	0.21	0.222	0.26	Optimal: 0.04-20L/kg; Range:<br><0.07L/kg: Confined to blood, bound to plasma protein<br>0.07-0.7L/kg: Evenly distributed;<br>>0.7L/kg: Bound to tissue components (e.g., protein, lipid).
BBB (Blood–Brain Barrier)	+ (0.966)	+ (0.935)	+ (0.936)	≥ 0.1: BBB+ < 0.1: BBB-
Subcellular localzation	Mitochondria (0.4798)	Mitochondria (0.4798)	Mitochondria (0.3602)	
	**Metabolism**			
CYP1A2 Inhibitor	+ (0.891)	+ (0.88)	+ (0.716)	
CYP1A2 substrate	+ (0.67)	+ (0.554)	- (0.498)	
CYP3A4 Inhibitor	- (0.39)	- (0.297)	- (0.299)	> 0.5: An inhibitor < 0.5: Non-inhibitor<br>> 0.5: Substrate < 0.5: Non-substrate
CYP3A4 substrate	+ (0.606)	+ (0.568)	+ (0.533)	
CYP2C9 Inhibitor	- (0.491)	- (0.429)	- (0.346)	

CYP2C9 substrate	- (0.508)	- (0.497)	- (0.423)	
CYP2C19 inhibitor	+ (0.808)	+ (0.782)	+ (0.665)	
CYP2C19 substrate	+ (0.626)	+ (0.574)	- (0.462)	
CYP2D6 inhibitor	+ (0.51)	- (0.451)	- (0.451)	
CYP2D6 substrate	- (0.43)	+ (0.51)	- (0.397)	
	**Excretion**			
Clearance (mL/min/kg)	Low 0.927	Low 0.894	Low 0.978	Range: > 15 high; 5 < Cl < 15: moderate; <5: low
T1/2 (Half life) (h)	Low 1.619	Low 1.612	Low 1.637	Range: > 8h: high; 3 h<Cl<8h: moderate; < 3h: low
	**Toxicity**			
hERG (hERG blockers)	- (0.291)	- (0.292)	- (0.266)	> 0.5: A Blocker < 0.5: Non-blocker
H-HT (Human Hepatotoxicity)	+ (0.566)	+ (0.558)	+ (0.686)	> 0.5: HHT positive < 0.5: HHT negative
AMES (Ames mutagenicity)	- (0.38)	+ (0.38)	- (0.458)	> 0.5: Positive < 0.5: Negative
LD50 (LD50 of acute toxicity)	2.562 -log mol/kg (817.532 mg/kg)	2.59 -log mol/kg (766.487 mg/kg)	2.737 -log mol/kg (592.239 mg/kg)	High-toxicity: 1~50 mg/kg; Toxicity: 51~500 mg/kg; low-toxicity: 501~5000 mg/kg.
